# Spalt-related is an inhibitor of mTORC1-mediated growth activated by the integrated stress response

**DOI:** 10.1038/s44318-026-00858-1

**Published:** 2026-07-11

**Authors:** Onur Deniz, Ying Liu, Tuuli Kirkinen, Krista Kokki, Kateryna Gaertner, Pau Clavell-Revelles, Jaakko Mattila, Ville Hietakangas

**Affiliations:** 1 Faculty of Biological and Environmental Sciences, University of Helsinki, Helsinki, Finland; 2 Institute of Biotechnology, University of Helsinki, Helsinki, Finland

**Keywords:** Development, Metabolism

## Abstract

Anabolic and catabolic processes are coordinated by a conserved regulatory network, which includes the nutrient-sensing protein kinase mTOR complex 1 (mTORC1) and the insulin- and stress-responsive transcription factor FoxO. In a physiological setting, these regulators align growth, storage, reproduction, and aging with nutrient availability. Here, we identify transcription factor Spalt-related (Salr), previously implicated in organogenesis, as a negative regulator of growth and lipid storage in *Drosophila melanogaster*. Salr activates catabolic gene expression and restricts mTORC1-mediated cell growth in the *Drosophila* fat body. The genomic binding of Salr overlaps extensively with that of FoxO, and a similar convergence is observed for their mammalian homologs, SALL1 and FOXO1. Both Salr and FoxO are activated upon fasting, but respond to distinct cues: while FoxO displays transient activation and is responsive to AKT inhibition, Salr is activated in a slow and sustained manner through the integrated stress response. Once activated, Salr counters nuclear localization of FoxO. Taken together, we show that Salr and FoxO are growth-inhibitory transcription factors that act in a convergent manner to respond to nutrient stress through distinct cues.

## Synopsis

How nutrient and stress signals coordinate growth and metabolism through integrated control of gene expression remains incompletely understood. This study identifies transcription factor Spalt-related (Salr) as an inhibitor of growth and lipid storage that is activated through the integrated stress response pathway upon prolonged fasting.

- Salr inhibits growth and lipid accumulation in *Drosophila* fat body by activating catabolic genes and restricting mTORC1 activity.
- The genomic binding of Salr extensively overlaps with that of FoxO, a relationship that is conserved between their mammalian homologs, SALL1 and FOXO1.
- Upon fasting, Salr is slowly and persistently activated via the integrated stress response pathway, in contrast to FoxO, which displays transient activation and is controlled through AKT signaling.
- Activated Salr suppresses FoxO nuclear localization, contributing to the attenuation of FoxO activity during prolonged fasting.

## Abstract (web summary)

Transcription factor Salr inhibits growth and lipid storage in the fat body of *Drosophila melanogaster* and counteracts FoxO during prolonged fasting.

## Graphical abstract

**Figure:**
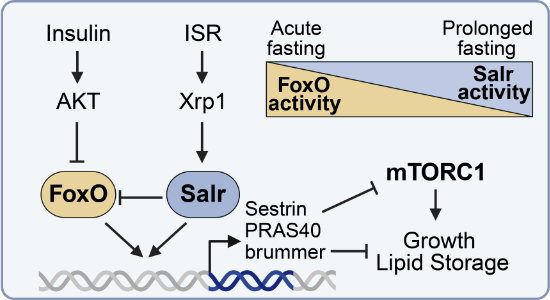

**Figure:**
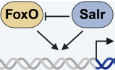

## Introduction

Metabolic pathway activities are tremendously adaptive to adjust cellular and systemic homeostasis in response to external conditions, including nutrient availability. Specific tissues, such as the liver in vertebrates and the fat body in insects, serve as metabolic hubs that coordinate systemic homeostasis. This sets high requirements for signal integration, i.e., an ability to simultaneously read and combine information from multiple inputs to generate a coordinated metabolic output. Impaired integration of nutrient-sensing signals can lead to systemic pathophysiologies, such as metabolic syndrome and type II diabetes (Klein et al, 2022; Priest and Tontonoz, 2019). Protein kinase mTOR complex 1 (mTORC1) is active when nutrients and energy are readily available, and it promotes the activity of anabolic pathways (Goul et al, 2023). mTORC1 activity is counteracted by the Forkhead box class O (FoxO) family of transcription factors, which activate catabolic genes (Xu et al, 2012) and inhibit mTORC1 activity, for example, by promoting the expression of its negative upstream regulator Sestrin (Lee et al, 2010). FoxO regulation integrates information from multiple sources: it is inhibited by insulin-like signaling through AKT-mediated phosphorylation (Brunet et al, 1999; Puig et al, 2003) and activated by several stress conditions, such as oxidative stress, heat, and genotoxic insults (Chen et al, 2010; Hopkins et al, 2025; Huang and Tindall, 2007; Rodriguez-Colman et al, 2024; Teleman et al, 2008). Despite these insights, we still lack a full understanding of how signal integration in metabolic hub organs is achieved in a physiological setting.

Spalt transcription factors are Zn-finger transcription factors with conserved regulatory roles in patterning and organogenesis (De Celis and Barrio, 2009). Mutations in two members of the human Sal-like (SALL) transcription factor family cause syndromes with impaired organogenesis, namely Townes-Brocks Syndrome (SALL1) and Okihiro Syndrome (SALL4) (Al-Baradie et al, 2002; Kohlhase et al, 1998). Their roles were originally discovered in *Drosophila*, where the two paralogs Spalt-major (Salm) and Spalt-related (Salr) regulate cell proliferation and survival downstream of Decapentaplegic (Dpp) signaling in the developing wing (De Celis et al, 1996; Sturtevant et al, 1997). Beyond development and organogenesis, however, the physiological roles of Spalt-like transcription factors are poorly understood.

Here, we report an unexpected role of *Drosophila* Salr as a fasting-activated inhibitor of growth and lipid storage in fat body cells. Salr is deeply integrated into the core nutrient-sensing regulatory network, displaying striking and conserved similarities with FoxO in target gene binding profiles. Salr targets include components of the mTORC1 pathway, such as *Sestrin* and *PRAS40*, and Salr is sufficient and necessary to restrict the growth-promoting activity of mTORC1 (Parmigiani et al, 2014; Wang et al, 2007). Salr and FoxO possess many similar functional properties, but they respond to distinct upstream cues. While FoxO is regulated by insulin-like signaling and displays rapid and transient activation upon fasting, Salr is activated through the integrated stress response (ISR) pathway during prolonged nutrient deprivation. Thus, our results imply that two converging pathways restrict growth, and they respond to fasting differentially depending on its duration and the upstream signaling cues involved.

## Results

### Inhibition of growth and lipid storage by Salr

**Figure 1:**
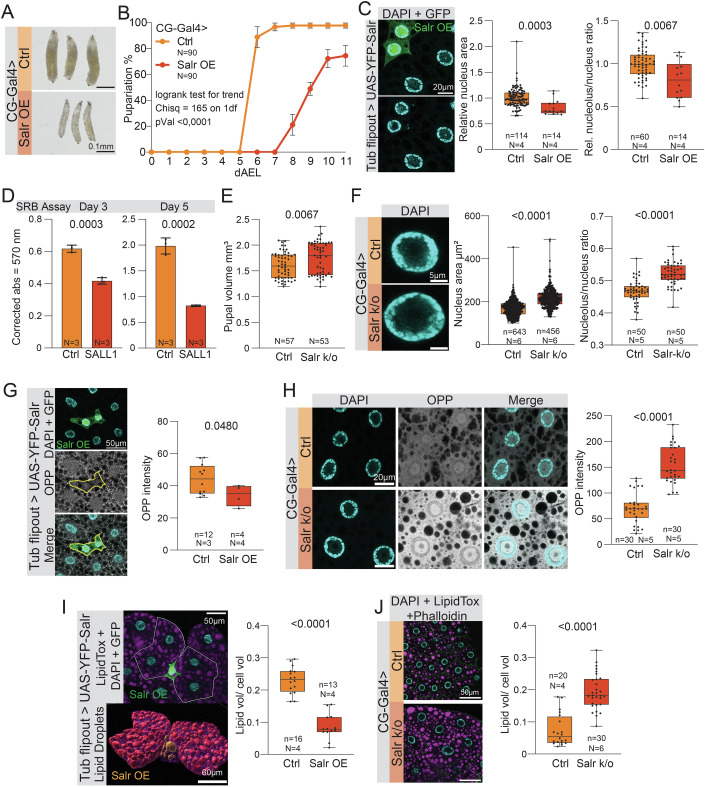
Salr inhibits cell growth and lipid storage cell autonomously in the fat body. (**A**) Representative image of early third instar larvae overexpressing Salr in the fat body. (**B**) Fat body-specific Salr overexpression leads to a delayed pupariation, dAEL: days after egg laying. (**C**) Salr inhibits cell growth autonomously. Representative image of Salr overexpressing clones (Tubulin flipout) marked with GFP and YFP. Quantification of relative nuclear and nucleolar sizes. (**D**) SALL1 overexpression in HEPG2 cells reduces cell proliferation. Quantification of sulforhodamine B (SRB) assay on days 3 and 5 after seeding. (**E**) Fat body-specific CRISPR/Cas12a knockout of Salr leads to increased pupal volume. (**F**) Fat body-specific CRISPR/Cas12a knockout of Salr leads to increased nuclear and nucleolar sizes. Representative image of Salr knockout and control nuclei stained with DAPI. Quantification of nuclear and nucleolar sizes. (**G**) Salr overexpression decreases protein synthesis. Representative image of GFP and YFP marked Salr overexpressing clones (Tubulin flipout) stained with DAPI and OPP. Quantification of OPP intensity in Salr overexpressing and control cells. (**H**) Salr CRISPR/Cas12a knockout in the fat body increases protein synthesis. Representative image of Salr knockout cells stained with DAPI and OPP. Quantification of OPP intensity of Salr knockout and control cells. (**I**) Salr overexpression decreases lipid droplet volume in fat body cells. Representative image of GFP and YFP marked Salr overexpressing clone (Tubulin flipout) stained with DAPI and LipidTox (above). 3D model of lipid droplets inside the white line (below). Lipid droplets of Salr overexpressing cell highlighted (orange). Quantification of the total lipid volume to cell volume ratio in Salr overexpressing cells. (**J**) Salr CRISPR/Cas12a knockout increases lipid droplet volume in fat body cells. Representative image of Salr knockout cells stained with DAPI, LipidTox, and Phalloidin. Quantification of lipid droplet volume to cell volume ratio. All experiments were replicated at least twice. *P* value and chi-square in (**B**) were calculated by log-rank test for trend. *P* values in (**C**–**J**) were calculated with unpaired *t* test with Welch correction. *N* = biological replicates, *n* = technical replicates. In box plots, the center line represents the median, the box boundaries represent the 25th and 75th percentiles, and the whiskers represent the minimum and maximum observed values. Error bars display standard deviations (SD).

**Figure EV1:**
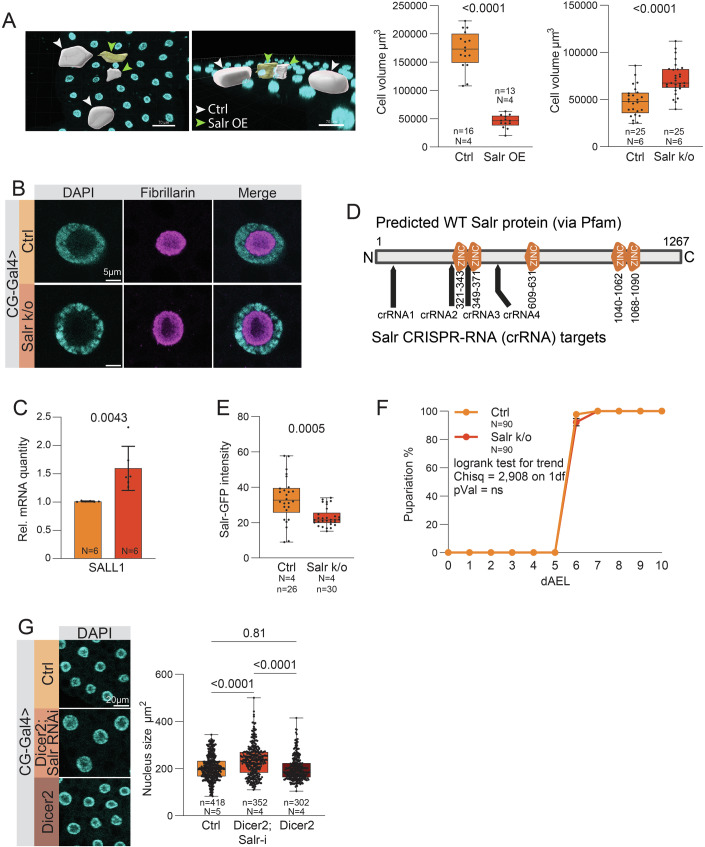
Salr inhibits cell growth in the fat body, related to Fig. 1. (**A**) Salr regulates cell size in the fat body. Representative images of volume quantification of Salr overexpressing clones (Tubulin flipout) and control cells. Quantification of cell volume upon gain- and loss-of-function of Salr in the fat body. (**B**) Representative images of anti-fibrillarin staining of Salr CRISPR/Cas12a knockout and control fat body cells. Fibrillarin localizes to the nucleolus and colocalizes with the DAPI-deficient area. (**C**) qPCR analysis of *SALL1* mRNA levels from SALL1 stably transfected HepG2 cells. (**D**) Salr CRISPR/Cas12a target sites on wild-type Salr protein. (**E**) Confirmation of Salr CRISPR/Cas12a knockout using GFP-tagged Salr reporter. (**F**) Fat body-specific Salr CRISPR/Cas12a knockout does not lead to accelerated pupariation. (**G**) Salr knockdown with Dicer2 increases nucleus size. Representative image of fat body cells that are Salr knockdown with Dicer2, control, and Dicer2, stained with DAPI. Quantification of nucleus size. *P* values in (**A**–**C**, **G**) were calculated with unpaired *t* test with Welch correction. *P* values in (**E**) were obtained by one-way ANOVA followed by Tukey’s test with multiple comparison correction. *P* value and chi-square in (**F**) were calculated by the log-rank test for trend. *N* = biological replicates, *n* = technical replicates. In box plots, the center line represents the median, the box boundaries represent the 25th and 75th percentiles, and the whiskers represent the minimum and maximum observed values. Error bars display standard deviations (SD).

We discovered a novel growth-inhibitory role for Spalt-related (Salr) in the *Drosophila* larvae. When overexpressed in the fat body (CG-Gal4), Salr was sufficient to delay larval growth (Fig. 1A) and pupariation (Fig. 1B). In addition to systemic growth regulation, Salr overexpression in fat body clones (Tubulin [flipout] Gal4) strongly reduced cell size, demonstrating cell autonomous function (Figs. 1C and EV1A). The growth inhibition was accompanied by smaller nuclear size, reflecting inhibition of endoreplication, as well as reduced relative size of the nucleolus (Figs. 1C and EV1B). To test for possible functional conservation, we generated HepG2 hepatocellular carcinoma cells, moderately overexpressing Salr ortholog SALL1 (Fig. EV1C). Consistent with the observations in *Drosophila*, the SALL1 overexpressing cells displayed impaired proliferation, compared to the control (Fig. 1D).

To study the Loss-Of-Function (LOF) phenotype of Salr and to overcome the early lethality of Salr full body mutants (Barrio et al, 1999) we generated Salr CRISPR/Cas12a transgenic flies, simultaneously targeting multiple regions of Salr (Fig. EV1D,E). Fat body-specific LOF of Salr did not affect pupariation rate but led to significantly increased pupal volume (Fig. 1E and EV1F). Consistent with the role as a growth inhibitor, nuclei in tissue-specific knockout fat body cells were significantly larger than in control animals (Fig. 1F), and they displayed significantly increased relative nucleolar size (Fig. 1F). These data were further confirmed by using fat body-specific knockdown by Salr RNAi (Fig. EV1G). The observed changes in nucleolar size suggested that Salr regulates ribosome biogenesis and consequently protein biosynthetic capacity. This was indeed the case as evidenced by O-propargyl-puromycin (OPP) labeling, reflecting reduced and elevated levels of newly synthesized proteins in Salr overexpressing and knockout cells, respectively (Fig. 1G,H).

As Salr overexpression in the fat body leads to smaller and thinner animals (Fig. 1A), we explored the effects of Salr on lipid storage by staining fat bodies using the neutral lipid stain LipidTox. 3D analysis of confocal images revealed that Salr overexpressing clones have strongly decreased total lipid droplet volume compared to neighboring control cells (Fig. 1I). Conversely, fat body-specific Salr knock-out increased total lipid droplet volume (Fig. 1J). In conclusion, Salr is necessary and sufficient to inhibit cell growth and lipid storage in the larval fat body.

### Salr activates a fasting-like transcriptional response

**Figure 2:**
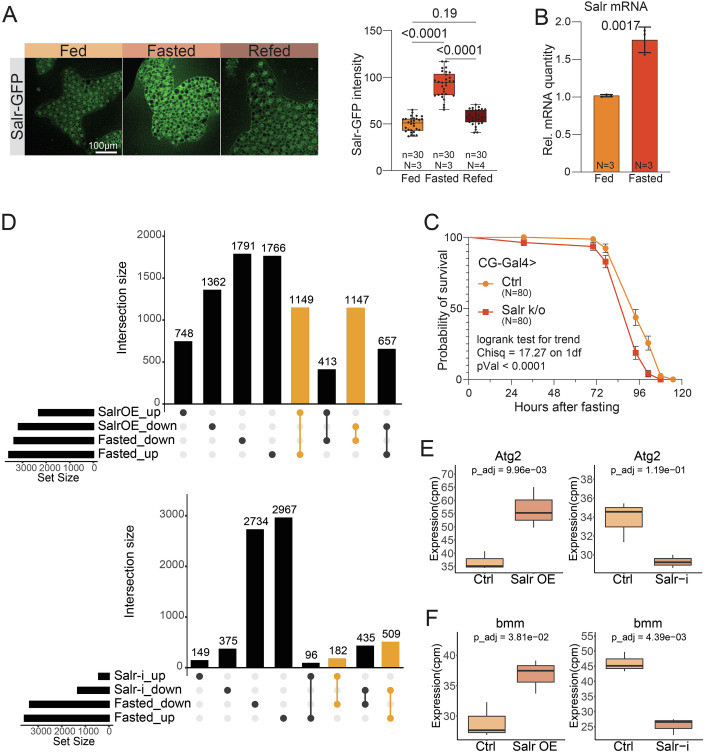
Salr is activated by fasting and activates catabolic gene expression. (**A**) GFP-tagged Salr reporter levels increase upon fasting and return to control levels after refeeding. Representative image and quantification of nuclear Salr-GFP under fed, 5 h fasting, and 6 h refeeding. (**B**) qPCR of *Salr* mRNA from fat bodies of fed and fasted animals. (**C**) Salr CRISPR/Cas12a knockout in the fat body leads to impaired survival under nutrient deprivation. (**D**) UpSet plots of fasting-induced differentially expressed genes (DEGs) and Salr gain- and loss-of-function DEGs. Highlighted intersections show fasting-mimicking gene regulation upon Salr gain- and loss-of-function. (**E**, **F**) Salr activates catabolic genes, including those involved in autophagy and lipid catabolism. *Atg2*, and *bmm* expression from RNA-Seq datasets upon fat body-specific Salr overexpression and knockdown. All experiments except RNA-Seq were replicated at least twice. *P* values in (A) were obtained by one-way ANOVA followed by Tukey’s test with multiple comparison correction. *P* value in (**B**) was calculated by unpaired *t* test with Welch correction. *P* value and chi-square in (**C**) were calculated by the log-rank test for trend. Adjusted *P* values in (**E**, **F**) were obtained in differential expression analysis of RNA-Seq with Benjamini–Hochberg correction. *N* = biological replicates, *n* = technical replicates. In box plots, the center line represents the median, the box boundaries represent the 25th and 75th percentiles, and the whiskers represent the minimum and maximum observed values. Error bars display standard deviations (SD).

**Figure EV2:**
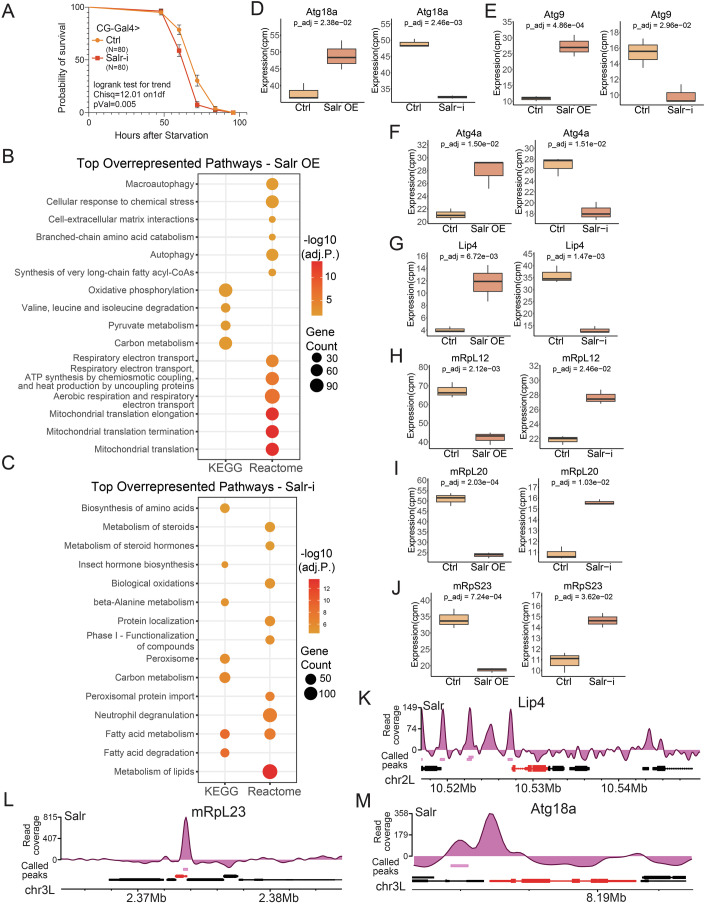
Salr regulates catabolic gene expression, related to Fig. 2. (**A**) Salr knockdown in the fat body impairs survival upon fasting. (**B**, **C**) Unselected top overrepresented pathways from fat body-specific Salr overexpression and knockdown RNA-Seq datasets, ranked based on their adjusted *P* values. (**D**–**M**) Salr overexpression and knockdown in the fat body significantly regulate genes involved in lipid metabolism, autophagy, mitochondrial ribosome biogenesis, some of which are direct targets of Salr. (**D**–**J**) *Atg18a*, *Atg9*, *Atg4a*, *Lip4*, *mRpL12*, *mRpL20*, *mRpL23* expression from RNA-Seq datasets upon fat body-specific Salr overexpression and knockdown. (**K**–**M**) Called peaks and track coverage of Salr ChIP-Seq on *Lip4, Atg18a*, and *mRpL23* genes highlighted in red. *P* value and chi-square in (**A**) were calculated by the log-rank test for trend. Adjusted *P* values in (**B**, **C**) were calculated by a one-sided hypergeometric test for over-representation analysis and adjusted using the Benjamini–Hochberg method. Adjusted *P* values in (**D**–**J**) were obtained in differential expression analysis of RNA-Seq with Benjamini–Hochberg correction. *N* = biological replicates. In box plots, the center line represents the median, the box boundaries represent the 25th and 75th percentiles, and the whiskers represent the minimum and maximum observed values. Error bars display standard deviations (SD).

As growth and lipid storage are closely controlled by nutrient availability, we wanted to test the possible nutrient regulation of Salr (Texada et al, 2020). As a reporter, we used ectopic Salr with a C-terminal GFP inserted into the genomic locus (Kudron et al, 2018). Indeed, Salr displayed significantly elevated expression in the fat body of fully nutrient-deprived larvae, and Salr expression was returned to control levels after refeeding (Fig. 2A). The elevated Salr expression upon fasting was confirmed by qPCR analysis of endogenous Salr mRNA levels (Fig. 2B). To explore the physiological role of Salr, we analyzed larval survival during starvation. Fat body-specific Salr knockout displayed modest but significant impaired resistance towards starvation (Fig. 2C). Similar starvation sensitivity was observed upon RNAi-mediated knockdown of Salr in the fat body (Fig. EV2A).

To obtain a global view of Salr downstream processes, we analyzed gene expression changes in the Salr gain- and loss-of-function fat bodies of early 3rd instar non-wandering larvae. Comparison with published datasets on starvation-regulated genes revealed that Salr activation in the fed state resembled the gene expression response observed in starved animals (Fig. 2D) (Hasygar et al, 2021). Overrepresentation analysis revealed that Salr activates catabolic responses, including genes involved in autophagy and lipid catabolism (Figs. 2E,F and EV2B–G). Conversely, Salr displayed a prominent inhibition of genes involved in mitochondrial ribosome biogenesis (Fig. EV2B,C,H–J). In conclusion, Salr is sufficient to activate a starvation-like transcriptional response in the fat bodies of fed larvae.

### Salr inhibits mTORC1 signaling

**Figure 3:**
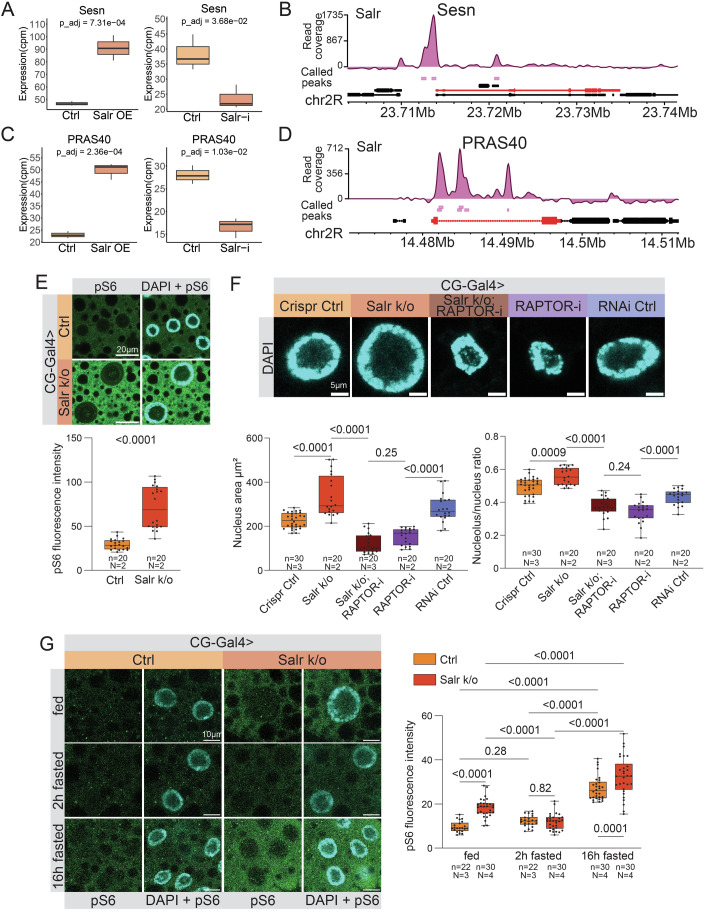
Salr inhibits growth by restricting mTORC1 activity. (**A**–**D**) Salr targets and upregulates negative upstream regulators of mTORC1, *Sesn*, and *PRAS40*. (**A**–**C**) *Sesn* and *PRAS40* expression from RNA-Seq datasets showing up- and downregulation upon fat body-specific Salr overexpression and knockdown, respectively. (**B**–**D**) Called peaks and track coverage of Salr ChIP-Seq on *Sesn* and *PRAS40* loci (highlighted with red). (**E**) Fat body-specific Salr CRISPR/Cas12a knockout leads to elevated mTORC1 activity. Representative images and quantification of Salr knockout cells stained with DAPI and phospho-S6. (**F**) Salr negatively regulates growth through mTORC1 signaling. Representative images of fat body nuclei of Salr CRISPR/Cas12a knockout in combination with Raptor RNAi. Quantification of nuclear and relative nucleolar sizes. (**G**) Fat body-specific Salr CRISPR/Cas12a knockout leads to elevated mTORC1 activity during feeding and prolonged fasting but not upon short-term fasting. Representative images and quantification of Salr knockout cells stained with DAPI and phospho-S6 on fed, 2 h fasted, and 16 h fasted conditions. All experiments except RNA-Seq were replicated at least twice. Adjusted *P* values in (**A**, **C**) were obtained by differential expression analysis of RNA-Seq with Benjamini–Hochberg correction. *P* value in (**E**) was calculated by unpaired *t* test with Welch correction. *P* values in (**F**, **G**) were obtained by one-way ANOVA followed by Tukey’s test with multiple comparison correction. *N* = biological replicates, *n* = technical replicates. In box plots, the center line represents the median, the box boundaries represent the 25th and 75th percentiles, and the whiskers represent the minimum and maximum observed values. Error bars display standard deviations (SD).

**Figure EV3:**
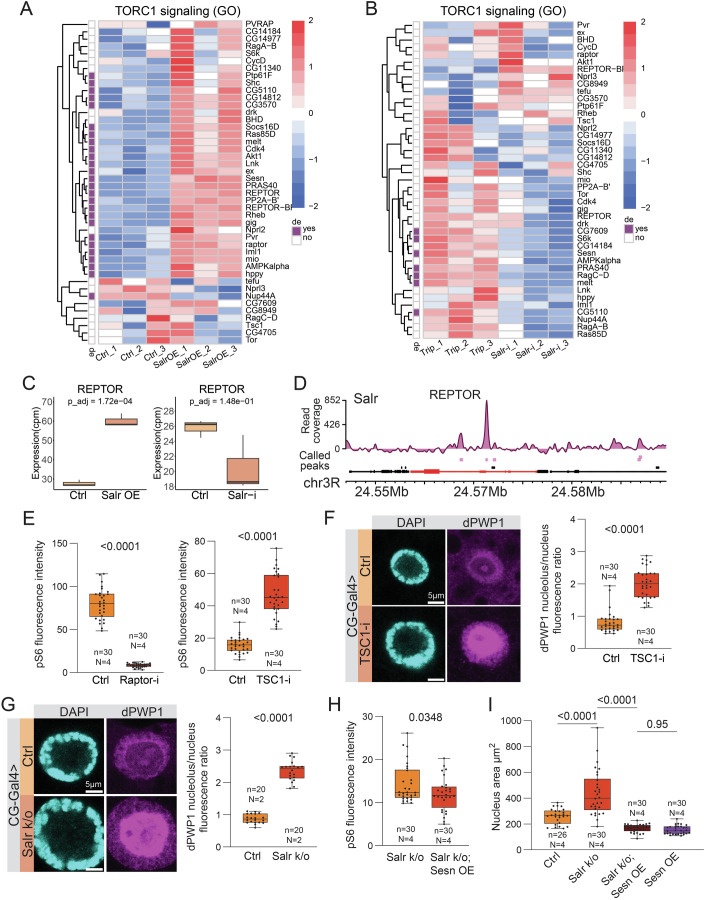
Salr regulates components of the mTORC1 pathway, related to Fig. 3. (**A**, **B**) Salr regulates the mTORC1 signaling pathway. Heatmaps of the GO database-defined mTORC1 signaling pathway from Salr overexpression and knockdown RNA-Seq datasets. (**C**, **D**) Salr targets and regulates *REPTOR* expression. (**C**) *REPTOR* expression from RNA-Seq showing up- and downregulation upon fat body-specific Salr overexpression and knockdown, respectively. (**D**) Called peaks and track coverage of Salr ChIP-Seq on *REPTOR* gene highlighted in red. (**E**) phospho-S6 (p-S6) antibody responds to mTORC1 genetic manipulation. Quantification of p-S6 signal of fat body-specific Raptor and TSC1 knockdown for inhibition and promotion of mTORC1, respectively. (**F**) PWP1 localizes to the nucleolus upon constitutive mTORC1 activation by fat body-specific TSC1 knockdown. Representative images of fat body-specific TSC1 knockdown and control fat body cells stained with PWP1 antibody. Quantification of nucleolar versus nucleoplasmic PWP1 ratio. (**G**) Fat body-specific Salr CRISPR/Cas12a knockout leads to relocalization of PWP1 into the nucleolus. Representative images of fat body-specific Salr CRISPR/Cas12a knockout and control fat body cells stained with PWP1 antibody. Quantification of nucleolar versus nucleoplasmic PWP1 ratio. (**H**, **I**) Fat body-specific overexpression of Sesn rescues Salr. (**H**) Quantification of phospho-S6 of fat body-specific Salr CRISPR/Cas12a knockout and in combination with Sesn overexpression. (**I**) Quantification of the nucleus size of fat body-specific Salr CRISPR/Cas12a knockout and in combination with Sesn overexpression. Adjusted *P* values in (**C**) were obtained in differential expression analysis of RNA-Seq with Benjamini–Hochberg correction. *P* values in (**E**–**H**) were calculated with unpaired *t* test with Welch correction. *P* values in (**I**) were obtained by one-way ANOVA followed by Tukey’s test with multiple comparison correction. *N* = biological replicates, *n*  = technical replicates. In box plots, the center line represents the median, the box boundaries represent the 25th and 75th percentiles, and the whiskers represent the minimum and maximum observed values. Error bars display standard deviations (SD).

To identify direct targets of Salr, we used the ModENCODE chromatin immunoprecipitation sequencing (ChIP-Seq) data from *Drosophila* prepupae (The modENCODE Consortium et al, 2010). Many of the genes in the Salr-regulated pathways in the fat body, including autophagy, lipid catabolism, and mitochondrial ribosome biogenesis, were indeed direct targets in the full body ChIP-Seq analysis (Fig EV2K–M). Interestingly, among the Salr direct target genes were several negative regulators of the mTOR complex 1 (mTORC1) pathway. These include *sestrin*, *pras40*, and *reptor*, which were upregulated upon Salr overexpression and downregulated by Salr knockdown in the fat body (Figs. 3A–D and EV3A–D).

The gene expression data suggest that Salr may promote a gene expression program to suppress mTORC1 signaling. To test this hypothesis, we used fat body-specific Salr CRISPR and monitored mTORC1 activity by antibody staining against phosphorylated ribosomal protein S6 (pS6), a well-established reporter of mTORC1 pathway activity (Isotani et al, 1999). To verify the responsiveness of the pS6 readout to the mTORC1 pathway in the larval fat body, we knocked down Raptor or TSC1 and observed an expected decrease and increase in the pS6 signal, respectively (Fig EV3E). Indeed, loss of Salr led to a prominent increase in S6 phosphorylation consistent with increased mTORC1 activity (Fig. 3E). To further confirm that Salr regulates mTORC1, we stained fat bodies with antibodies against PWP1, a known regulator of ribosome biogenesis that localizes to the nucleolus in an mTORC1-dependent manner (Liu et al, 2017) (Fig. EV3F). Consistent with mTORC1 activation, we observed that fat body-specific Salr knockout led to highly elevated nucleolar PWP1 levels (Fig. EV3G). To test if Salr-mediated growth regulation is mTORC1-dependent, we performed a genetic epistasis experiment by simultaneous inhibition of Salr and mTORC1, using either Raptor knockdown or Sesn overexpression (Kim et al, 2002; Wolfson et al, 2016). Our results show that inhibiting mTORC1 fully suppressed the Salr LOF-induced increase in nucleus and nucleolus size (Figs. 3F and EV3H,I), further supporting the conclusion that Salr inhibits growth by restricting mTORC1 activity in the *Drosophila* fat body.

Finally, we wanted to analyze the contribution of Salr to the dynamics of mTORC1 activity during fasting. While Salr knockout elevated mTORC1 activity in the fed state, this was overridden by acute (2 h) fasting (Fig. 3G). Surprisingly, however, mTORC1 signaling was reactivated during prolonged (16 h) fasting, and in the absence of Salr this reactivation was strongly pronounced. While the mTORC1 reactivation during prolonged starvation appears counterintuitive, it is notable that similar observations have been made earlier in mouse liver (Kaade et al, 2024). In conclusion, Salr modulates mTORC1 signaling during the fed state and prolonged fasting, but its regulatory role is overridden by other inhibitory mechanisms during acute fasting.

### Salr and FoxO share common direct targets

**Figure 4:**
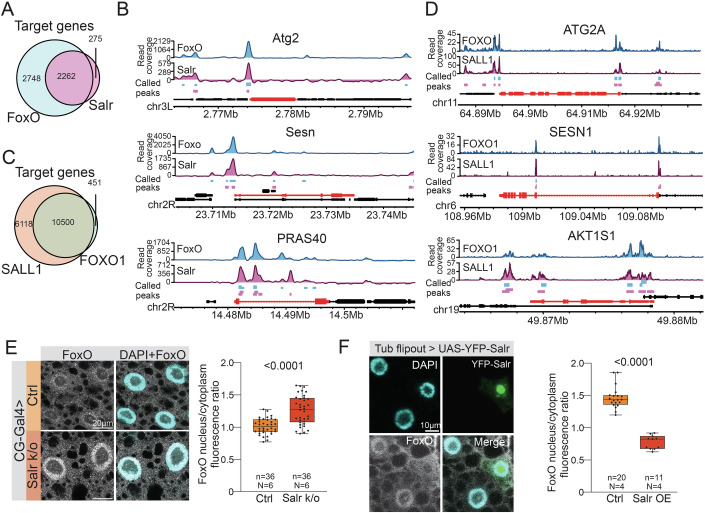
Salr shares common targets with and antagonizes FoxO. (**A**–**D**) Salr and FoxO share common direct targets in *Drosophila* and humans. (**A**) Venn diagram of direct targets of FoxO and Salr. (**B**) Called peaks and coverage tracks of Salr and FoxO ChIP-Seq on *Atg2*, *Sesn*, and *PRAS40* loci (highlighted in red). (**C**) Venn diagram of direct targets of human SALL1 and FOXO1. (**D**) Called peaks and coverage tracks of human *SALL1* and *FOXO1* on *ATG2A*, *SESN1*, and *AKT1S1* (*PRAS40* ortholog) loci (highlighted in red). (**E**, **F**) Salr antagonizes the nuclear localization of FoxO. (**E**) Representative image of fat body-specific Salr CRISPR/Cas12a knockout and control fat body cells stained with anti-FoxO antibodies. Quantification of the nuclear versus cytoplasmic FoxO ratio. (**F**) Representative image of Salr overexpressing clone stained with anti-FoxO antibodies. Quantification of the nuclear versus cytoplasmic FoxO ratio. All experiments were replicated at least twice. *P* values in (**E**, **F**) were calculated with unpaired *t* test with Welch correction. *N* = biological replicates, *n* = technical replicates. In box plots, the center line represents the median, the box boundaries represent the 25th and 75th percentiles, and the whiskers represent the minimum and maximum observed values. Error bars display standard deviations (SD).

**Figure EV4:**
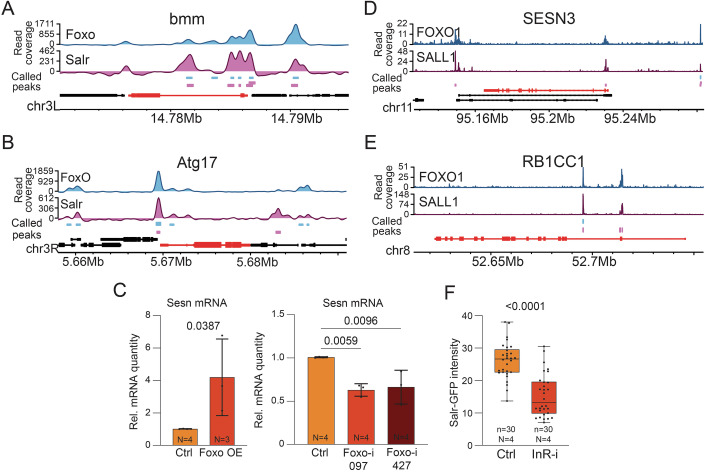
Salr shares common targets with FoxO, related to Fig. 4. (**A**) Called peaks and track coverage of Salr and FoxO ChIP-Seq on *bmm*. (**B**) Called peaks and track coverage of Salr and FoxO ChIP-Seq on *Atg17*. (**C**) FoxO positively regulates Sesn. qPCR of Sesn mRNA in FoxO gain- and loss-of-function fat bodies. Foxo-i 097: VDRC 106097, Foxo-i 427: BDSC 32427. (**D**) Called peaks and track coverage of SALL1 and FOXO1 ChIP-Seq on *SESN3* (one of the human homologs of *Drosophila* Sesn). (**E**) Called peaks and track coverage of SALL1 and FOXO1 ChIP-Seq on *RB1CC1* (human ortholog of Atg17). (**F**) InR knockdown does not activate Salr. Quantification of Salr-GFP intensity of fat body-specific InR knockdown and control. *P* values in (**C**) (left panel) and (**F**) were calculated with unpaired *t* test with Welch correction. *P* values in (**C**) (right panel) were obtained by one-way ANOVA followed by Tukey’s test with multiple comparison correction. *N* = biological replicates, *n* = technical replicates. In box plots, the center line represents the median, the box boundaries represent the 25th and 75th percentiles, and the whiskers represent the minimum and maximum observed values. Error bars display standard deviations (SD).

The nutrient responsiveness and target genes of Salr prompted us to explore its relationship with known regulators of nutrient-responsive transcription. FoxO transcription factor is a well-known downstream target of insulin-like signaling and regulator of catabolic processes (Puig and Mattila, 2011). A genome-wide comparison of chromatin binding revealed a striking overlap between FoxO and Salr targets: 89% of Salr target genes were shared with FoxO (Fig. 4A). Common targets include *atg2* and *atg17*, *brummer* (*bmm*), *PRAS40* and *Sestrin (Sesn)*, encoding components of autophagy, lipid catabolism, and mTORC1 signaling pathway, respectively (Figs. 4B and EV4A,B). In addition to targeting the same genes, the local chromatin binding profiles of Salr and FoxO to the *atg2*, *PRAS40*, *bmm*, *Sesn*, *atg17* gene regions were nearly identical, implying close functional cooperation (Figs. 4B and EV4A,B). To confirm the role of FoxO in fat body gene regulation, we tested its impact on *sestrin* gene expression (Fig. EV4C), which verified FoxO as an activator of this growth-inhibitory gene, similar to Salr.

To analyze the possible conservation of Salr and FoxO target gene regulation, we analyzed ChIP-Seq data derived from human HepG2 hepatocellular carcinoma cells (The ENCODE Project Consortium, 2012). A noticeable overlap between binding profiles of Salr homolog SALL1 and FoxO homolog FOXO1 was observed: 63% of SALL1 targets were shared with FOXO1, while 95% of FOXO1 targets were common with SALL1 (Fig. 4C). Moreover, SALL1 and FOXO1 shared many conserved targets with their *Drosophila* orthologs, including *atg2* (ATG2A), *PRAS40* (AKT1S1), *atg17* (RB1CC1), *Sesn* (SESN3, SESN1) (Figs. 4D and EV4D,E). These data suggest that the regulation of common target genes by Salr and FoxO is conserved in mammals.

To investigate the interplay between Salr and FoxO functionally in *Drosophila*, we monitored intracellular localization of endogenous FoxO in Salr loss- and gain-of-function fat body cells. Surprisingly, FoxO nuclear localization correlated negatively with Salr expression. Upon fat body-specific Salr loss-of-function, FoxO showed increased nuclear/cytoplasmic ratio (Fig. 4E). In contrast, Salr gain-of-function led to reduced nuclear/cytoplasmic ratio of FoxO (Fig. 4F). In sum, Salr binds to genomic regions that overlap with those of FoxO and functionally antagonizes its nuclear localization.

### Salr is activated by prolonged fasting through the integrated stress response pathway

**Figure 5:**
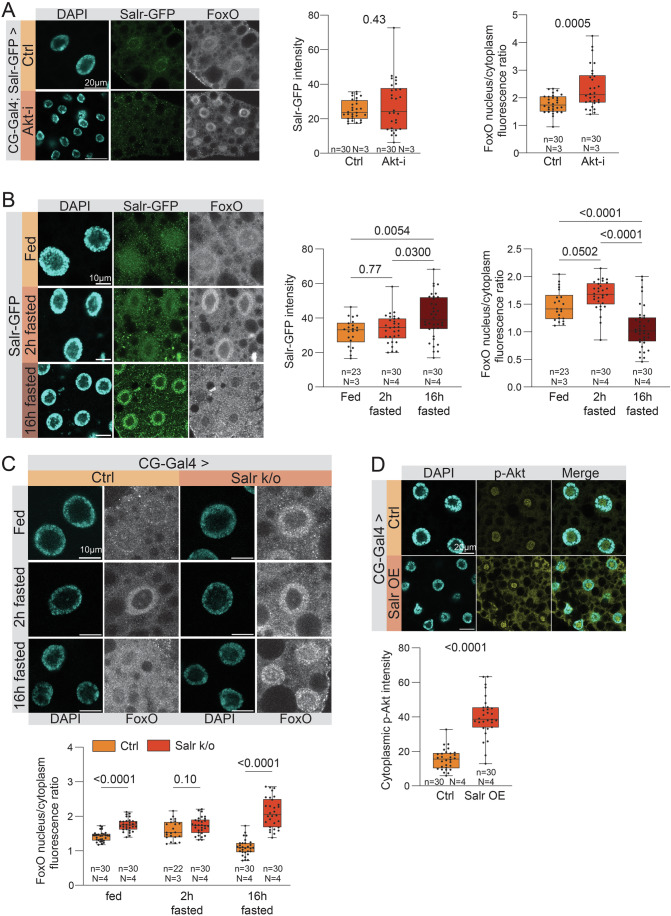
Salr and FoxO show different activation dynamics upon fasting. (**A**) Akt knockdown does not increase Salr protein levels, while promoting nuclear localization of FoxO. Representative images of fat body-specific Akt knockdown stained with anti-FoxO antibodies and anti-GFP antibodies for Salr-GFP. Quantification of Salr-GFP and nuclear versus cytoplasmic FoxO ratio. (**B**) Differential activation dynamics of FoxO and Salr upon fasting. Representative images of fat body cells stained with anti-FoxO antibodies and anti-GFP antibodies for Salr-GFP after 2 h and 16 h fasting. Quantification of nuclear Salr-GFP and nuclear versus cytoplasmic FoxO ratio. (**C**) Salr regulates FoxO activity. Representative images of fat body-specific Salr knockout and control fat body cells stained with anti-FoxO antibody. Quantification of nuclear versus cytoplasmic FoxO ratio. (**D**) Salr overexpression in the fat body elevates phospho-Akt (p-Akt) levels. Representative image of fat body-specific Salr overexpression and control fat body cells stained with p-Akt antibody. Quantification of cytoplasmic p-Akt signals. All experiments were replicated at least twice. *P* values in (**A**, **D**) were calculated with unpaired *t* test with Welch correction. *P* values in (**B**, **C**) were obtained by one-way ANOVA followed by Tukey’s test with multiple comparison correction. *N* = biological replicates, *n* = technical replicates. In box plots, the center line represents the median, the box boundaries represent the 25th and 75th percentiles, and the whiskers represent the minimum and maximum observed values. Error bars display standard deviations (SD).

The antagonistic role of Salr on nuclear FoxO led us to postulate that while these factors target a common set of genes, they might be activated by distinct physiological stimuli. Consistent with this, knockdown of Akt, the transducer of insulin-like signaling and a negative regulator of FoxO, along with knockdown of InR, did not activate Salr expression (Figs. 5A and EV4F). Moreover, the activation dynamics of Salr and FoxO during fasting differed: while FoxO nuclear localization was increased by acute (2 h) fasting, Salr expression was highly activated only after prolonged fasting (16 h) (Fig. 5B). Interestingly, the nuclear FoxO localization was significantly attenuated upon prolonged fasting, consistent with the observed inhibition of nuclear FoxO by Salr expression (Fig. 5B).

**Figure EV5:**
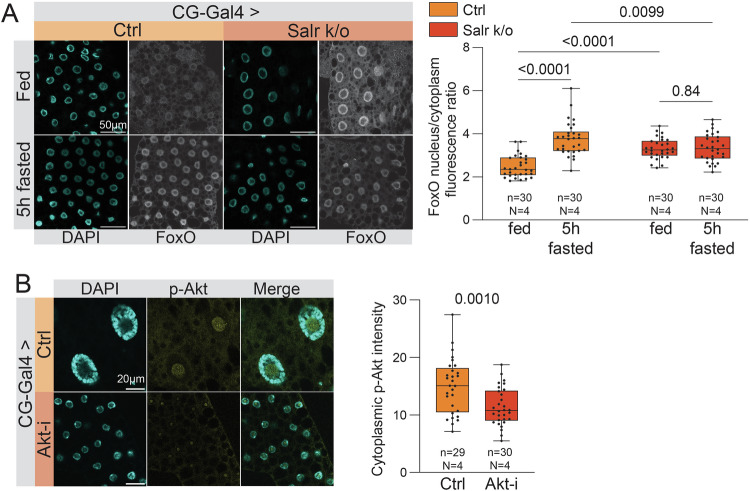
Salr and FoxO show different activation dynamics upon fasting, related to Fig. 5. (**A**) Salr CRISPR/Cas12a knockout does not activate FoxO compared to control at 5 h fasting. Representative images of Salr CRISPR/Cas12a knockout and control stained with FoxO in fat body-specific cells. Quantification of nuclear versus cytoplasmic FoxO signal. (**B**) Cytoplasmic p-Akt signal is reduced, unlike the nucleolar signal, upon Akt knockdown in the fat body. Representative images of fat body-specific Akt knockdown and control stained with p-Akt. Quantification of cytoplasmic p-Akt signal. *P* values in (**A**) were calculated with unpaired *t* test with Welch correction. *P* values in (**B**) were obtained by one-way ANOVA followed by Tukey’s test with multiple comparison correction. *N* = biological replicates, *n* =  technical replicates. In box plots, the center line represents the median, the box boundaries represent the 25th and 75th percentiles, and the whiskers represent the minimum and maximum observed values. Error bars display standard deviations (SD).

To directly test the causal role of Salr on FoxO regulation during fasting, we kinetically analyzed the nuclear/cytoplasmic ratio of FoxO in the presence and absence of Salr. In contrast to the observed FoxO attenuation in control animals, nuclear FoxO localization was sustained upon prolonged fasting in the Salr LOF, providing direct evidence for the role of Salr in the attenuation of FoxO (Figs. 5C and EV5A). Because FoxO localization is controlled by Akt-mediated phosphorylation (Hietakangas and Cohen, 2007; Mattila et al, 2008), we wondered whether Salr regulates Akt activity. Indeed, overexpression of Salr led to increased Akt S505 phosphorylation (Figs. 5D and EV5B), consistent with the observed Salr-mediated inhibition of nuclear FoxO.

**Figure 6:**
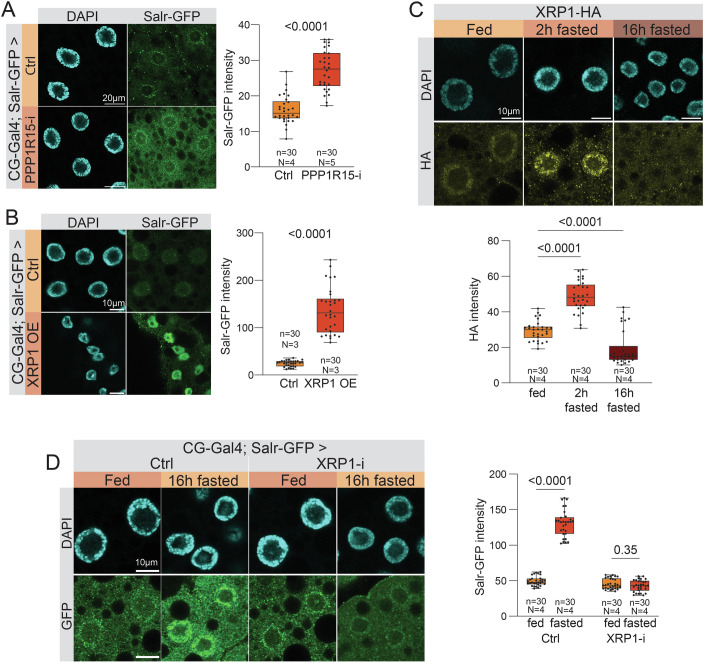
Prolonged fasting activates Salr through the integrated stress response pathway. (**A**) Activation of the integrated stress response (ISR) pathway by PPP1R15 knockdown in the fat body activates Salr. Representative image of fat body-specific knockdown of PPP1R15 and control fat body cells with anti-GFP for Salr-GFP. Quantification of nuclear Salr-GFP. (**B**) Overexpression of XRP1 in the fat body increases Salr expression. Representative image of cells overexpressing XRP1 and control fat body cells, stained with anti-GFP for Salr-GFP. Quantification of nuclear Salr-GFP. (**C**) XRP1 is dynamically regulated by fasting. Representative image and quantification of the nuclear XRP1-HA under fed, 2 h, and 16 h fasting. (**D**) XRP1 is necessary for fasting-induced Salr expression. Representative image of fat body-specific knockdown of XRP1 (BDSC 34521) and control fat body cells with anti-GFP for Salr-GFP. Quantification of nuclear Salr-GFP. All experiments were replicated at least twice. *P* values in (**A**, **B**) were calculated with an unpaired *t* test with Welch correction. *P* values in (**C**, **D**) were obtained by one-way ANOVA followed by Tukey’s test with multiple comparison correction. *N* =  biological replicates, *n* = technical replicates. In box plots, the center line represents the median, the box boundaries represent the 25th and 75th percentiles, and the whiskers represent the minimum and maximum observed values. Error bars display standard deviations (SD).

**Figure EV6:**
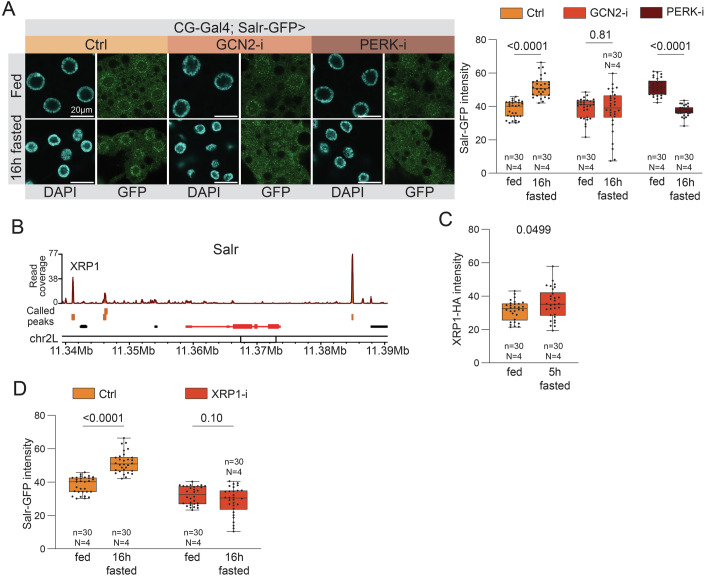
Prolonged fasting activates Salr through the integrated stress response pathway, related to Fig. 6. (**A**) Fat body-specific knockdown of GCN2 and PERK prevents Salr activation upon 16 h fasting. Representative images of GCN2 and PERK RNAi, and control fat bodies stained with GFP for Salr-GFP. Quantification of nuclear Salr-GFP. (**B**) XRP1 targets Salr. Called peaks and track coverage of XRP1 ChIP-Seq on *Salr* gene which is highlighted in red. (**C**) XRP1 level is moderately elevated upon 5 h fasting. Quantification of nuclear XRP1-HA. (**D**) XRP1 is necessary for Salr activation upon prolonged fasting. Validation of the data presented in Fig. 6D by an independent RNAi line (BDSC 51054). Quantification of Salr-GFP. P values in (**A**–**D**) were calculated with an unpaired *t* test with Welch correction. In box plots, the center line represents the median, the box boundaries represent the 25th and 75th percentiles, and the whiskers represent the minimum and maximum observed values. Error bars display standard deviations (SD).

How is Salr activated upon prolonged fasting? Chronic nutrient shortage is known to activate the integrated stress response (ISR), leading to elevated phosphorylation of eIF2α (Ryoo, 2024). The ISR is counteracted by protein phosphatase PPP1R15 (GADD34), which dephosphorylates eIF2α (Malzer et al, 2013). Knockdown of PPP1R15 led to significantly elevated Salr expression, supporting the idea of ISR-mediated activation of Salr (Fig. 6A). Consistently, knockdown of ISR kinases GCN2 and PERK prevented Salr activation during fasting (Fig. EV6A).

One of the downstream effectors of ISR is the transcription factor Xrp1 (Brown et al, 2021), which we found to display chromatin binding close to the *salr* locus (Fig. EV6B). Overexpression of Xrp1 in the fat body led to substantial fat body hypotrophy (Fig. 6B), possibly due to its known effects in cell survival (Blanco et al, 2020). In line with the model of Xrp1 being an upstream activator of Salr, overexpression of Xrp1 led to markedly increased Salr expression (Fig. 6B). We further tested the possibility of Xrp1 nutrient regulation and observed transiently elevated expression during fasting, which attenuated during prolonged fasting (Figs. 6C and EV6C). Consistent with the hypothesis of Salr regulation by fasting-induced Xrp1 activation, knockdown of Xrp1 abolished the elevated Salr expression by prolonged fasting (Figs. 6D and EV6D). Collectively, these data are consistent with a model that Salr and FoxO are activated by nutrient deprivation with differential kinetics and controlled by distinct upstream cues. Salr displays slow and sustained activation downstream of the ISR effector Xrp1, which is transiently activated during fasting, similar to FoxO.

## Discussion

This study provides evidence for two functionally converging stress response pathways that activate catabolic gene expression and restrict the activity of anabolic mTORC1 signaling. These pathways, controlled by Salr and FoxO transcription factors, respond to distinct upstream signals and are activated upon fasting with differential kinetics, FoxO displaying fast and transient activation and Salr being activated upon prolonged nutrient deprivation. Their functions appear mutually exclusive, as high expression of Salr during prolonged fasting promotes the attenuation of nuclear FoxO. ChIP-Seq data revealed high degree of overlap between the binding profiles, but future studies employing high-resolution binding site analysis are needed for more detailed insight for the molecular interplay between Salr and FoxO in the target promoters. The observed role for Salr as a regulator of metabolism and cell growth may appear surprising, regarding its well-established role in organogenesis (De Celis and Barrio, 2009). However, similar examples exist, including Cabut, a regulator of Dpp signaling in wing patterning, which also controls metabolic target genes as a downstream effector of intracellular sugar sensing and the circadian clock (Bartok et al, 2015; Bejarano et al, 2008).

Our data show that Salr functions downstream of ISR and Xrp1, and it restricts mTORC1 activity in fat body cells. We observed that Xrp1 undergoes rapid and transient activation upon fasting and is necessary and sufficient to activate Salr, which displays a gradual and sustained activation pattern. Future work is needed to comprehensively address the role of fat body Xrp1 nutrient-responsive regulation of growth and metabolism, beyond its reported role as an upstream regulator of Salr. Previous work has shown that Xrp1 acts as a regulator of cell competition in developing *Drosophila* wing, being activated by proteotoxic stress and promoting the ‘loser’ status of cells destined to be eliminated (Baillon et al, 2018; Lee et al, 2018; Recasens-Alvarez et al, 2021). Interestingly, inhibiting mTORC1 activity can alleviate the elimination of cells with proteotoxic stress through stimulation of autophagy and allowing clearance of misfolded proteins (Recasens-Alvarez et al, 2021). Considering the observed role of Salr as a downstream effector of Xrp1 as well as a negative regulator of mTORC1 and activator of autophagy genes, it will be interesting to learn whether Salr contributes to proteotoxicity-induced cell competition in the wing imaginal disc.

ChIPseq data derived from human hepatocellular carcinoma (HCC) cells revealed pervasive overlap in genome-wide binding profiles of SALL1 and FOXO1, including the binding to promoters of mTORC1 upstream regulators, which suggests conservation of the observed functions in humans. FOXO1 is a well-known tumor suppressor, and its expression is often reduced in HCC, which is associated with poor prognosis (Shi et al, 2018). Interestingly, SALL1 was also recently shown to function as a tumor suppressor in HCC and is sufficient to inhibit anchorage-dependent colony formation when highly overexpressed (Saito et al, 2025). Our findings in HepG2 cells confirmed these findings and showed that even moderately elevated expression of SALL1 is sufficient to potently inhibit the proliferation of HCC cells. Considering that mTORC1 is a major driver in HCC, being deregulated in about 50% of cases (Yu et al, 2019), it will be interesting to explore whether SALL1 and FOXO1 functionally converge to restrict mTORC1 activity in HCC, thereby suppressing tumorigenesis.

In conclusion, our findings establish Salr, previously linked to organogenesis, as a core component of the regulatory network governing nutrient-responsive growth control. Through its role as an ISR-activated inhibitor of mTORC1 and FoxO, Salr function reveals previously unrecognized complexity in the interconnections between the core regulators of cellular homeostasis and their activation/attenuation dynamics during fasting. Future studies should address the role of Salr in modulating stress responses beyond nutrient deprivation.

## Methods

**Table:** Reagents and tools table

Reagent/resource	Reference or source	Identifier or catalog number
**Experimental models**		
HEPG2	Leibniz Institute DSMZ; Gift from Maria Lindahl	ACC 180
HEPG2-pCMV3-untagged (HEPG2-Contr)	This study	N/A
HEPG2-SALL1	This study	N/A
UAS-Salr-crRNA (in ZH-51C: 2 R 51C1)	This study	N/A
UAS-Cas12a (in attP40: 2 L 25C7)	This study	N/A
UAS-pCFD8 (in ZH-51C: 2 R 51C1)	This study	N/A
w1118	BDSC	6326
UAS-YFP-Salr	Gift from Rosa Barrio (Sánchez et al, 2010)	N/A
Salr-GFP	BDSC	66443
UAS-Salr-RNAi	BDSC	29549
UAS-Dicer2	BDSC	24650
UAS-XRP1	FLYORF (Bischof et al, 2013)	F003459
XRP1-HA	Gift from Nicholas Baker (Blanco et al, 2020)	N/A
UAS-XRP1-RNAi	BDSC	34521
UAS-XRP1-RNAi	BDSC	51054
UAS-Raptor-RNAi	BDSC	31529
UAS-Sesn-FLAG	BDSC	91235
UAS-TSC1-RNAi	VDRC	110811
UAS-FoxO-RNAi	BDSC	32427
UAS-FoxO-RNAi	VDRC	106097
UAS-FoxO-GFP	Hietakangas and Cohen, 2007	N/A
UAS-Akt-RNAi	BDSC	33615
UAS-InR-RNAi	BDSC	51518
UAS-PPP1R15-RNAi	VDRC	107545
UAS-GCN2-RNAi	BDSC	35355
UAS-PERK-RNAi	BDSC	42499
UAS-Luciferase-RNAi (control for BDSC RNAi lines)	BDSC	31603
KK-line Control (Control for VDRC RNAi lines)	VDRC	60100
UAS-EGFP	BDSC	6874
CG-Gal4	BDSC	7011
**Recombinant DNA**		
pCMV3-untagged	Sino Biological	CV011
pCMV3-SALL1	Sino Biological	HG18646-UT
pCFD8	Addgene	140619
UAS-Cas12a	Addgene	140621
**Antibodies**		
anti-FoxO (1:500)	Gift from O. Puig (Puig et al, 2003)	N/A
anti-GFP (1:500)	Abcam	ab13970
anti-phospho-S6 (1:500)	Cell Signaling Technology	2211
anti-Fibrillarin (1:500)	Abcam	ab4566
anti-phospho-Akt (1:500)	Cell Signaling Technology	4054
anti-PWP1 (1:500)	Gift from Mark Van Doren (Casper et al, 2011)	N/A
anti-HA (1:500)	Abcam	ab9110
**Oligonucleotides and other sequence-based reagents**		
Salr qPCR primer 1	CAAGATCTTTGGCAGCTACTCGGC	N/A
Salr qPCR primer 2	CCTTCAGATTGCCTTTCGTGGTG	N/A
CDK7 qPCR primer 1	GGGTCAGTTTGCCACAGTTT	N/A
CDK7 qPCR primer 2	GATCACCTCCAGATCCGTG	N/A
Sesn qPCR primer 1	TTCACCAGATACGGACACT	N/A
Sesn qPCR primer 2	TCCGCTGCCTAACGATTAC	N/A
GAPDH qPCR primer 1	TCGGAGTCAACGGATTTGGT	N/A
GAPDH qPCR primer 2	TTGCCATGGGTGGAATCATA	N/A
SALL1 qPCR primer 1	GAACTTGACGGGATTGCCTCCT	N/A
SALL1 qPCR primer 2	GCTTGCACTATTTGTGGAAGAGC	N/A
Salr gRNA1	CCGATCTCGATCACGATCCCTGT	N/A
Salr gRNA2	GTCCGGCGGTCGGGAATCACTGC	N/A
Salr gRNA3	CTTCCGCAAACATTACAAACGAA	N/A
Salr gRNA4	CTTAACCATTGGCTTGCTGAGAT	N/A
**Chemicals, enzymes, and other reagents**		
LipidTox (1:400)	Thermo Fisher Scientific	H34477
Click-iT® Plus OPP (1:400)	Thermo Fisher Scientific	C10456
Shields and Sang M3 Insect Medium	Merck	S3652
Vectashield	Vector Laboratories	H-1200
Roswell Park Memorial Institute (RPMI) 1640 medium	Institute of Biotechnology Media Kitchen, HiLIFE, Helsinki	N/A
Dulbecco’s Phosphate Buffered Saline (DPBS)	Sigma-Aldrich	D8537
fetal bovine serum	Sigma-Aldrich	F7524
penicillin-streptomycin	Gibco	15140-122
GlutaMAX	Gibco	35050061
jetPRIME reagent	Sartorius	101000015
hygromycin B	Sigma-Aldrich	SBR00039
formaldehyde, methanol-free (4% diluted in DPBS)	Thermo Scientific	28908
sulforhodamine B sodium salt	Sigma-Aldrich	S9012
Tris base	Sigma-Aldrich	252859
Trypsin-EDTA (0.05% diluted in DPBS)	Gibco	15400054
RevertAid H Minus First Strand cDNA Synthesis Kit	Thermo Fisher Scientific	K1632
Maxima SYBR Green qPCR Master Mix	Thermo Fisher Scientific	K0221
TruSeq Stranded mRNA Library Prep Kit	Illumina	20020594
Nucleospin RNA kit	Macherey-Nagel	740984.50
**Software**		
Imaris	Oxford Instruments	https://imaris.oxinst.com/
Fiji/ImageJ	(Schindelin et al, 2012)	https://imagej.net/software/fiji/
R	R-Project	https://www.r-project.org/
GraphPad Prism 10	GraphPad Software	https://www.graphpad.com/
RStudio	RStudio	https://posit.co/download/rstudio-desktop
**Deposited data**		
Salr mRNA sequencing	This study	GEO: GSE123901
Salr ChIP-Seq	(The ENCODE Project Consortium, 2012)	GSE257049
FoxO ChIP-Seq	(The ENCODE Project Consortium, 2012)	GSE258971
SALL1 ChIP-Seq	(The ENCODE Project Consortium, 2012)	GSE231113
FOXO1 ChIP-Seq	(The ENCODE Project Consortium, 2012)	GSE170347

### *Drosophila* husbandry

Flies were maintained at 25 °C, on standard laboratory food containing agar 0.6% (w/v), malt 6.5% (w/v), semolina 3.2% (w/v), baker’s yeast 1.8% (w/v), nipagin 2.4%, and propionic acid 0.7%. To generate mosaic clones, larvae with the genotype HsFLP/+; UAS-YFP-Salr/UAS-GFP; Tub<Gal80, y^+^>Gal4/+ were heat shocked at 37 °C for 12 min at 24 h after egg laying.

### CRISPR/Cas12a-mediated tissue-specific mutagenesis

Four guide RNAs targeting different locations of the Salr coding region (Fig. EV1D) were designed and cloned into the pCFD8 plasmid as described in (Port et al, 2020). The Salr gRNA-pCFD8 plasmid was inserted into the ZH-51C (2 R 51C1) site. An empty pCFD8 plasmid was inserted at the same site as a control for CRISPR experiments. UAS-Cas12a plasmid was inserted into the attP40 (2 L 25C7) site, and the UAS-Cas12a and UAS-Salr-crRNA were recombined to be crossed with CG-Gal4 for fat body-specific knockout of Salr.

### Nutrient deprivation of larvae

For fasting experiments, age-matched L1 larvae were reared on standard laboratory food at equal density (30/vial) for 2 days. The larvae were then washed with PBS and transferred into a 48-well plate containing fasting medium of 0.5% (w/v) agar supplemented with 0.2% (v/v) erioglaucine dye for the times indicated in the figure panels. The plate was covered with film, and small holes were opened for air circulation.

### Immunohistochemistry, LipidTox, and OPP staining

For immunofluorescence staining, fat bodies were dissected from pre-wandering L3 larvae (96 h AEL) in PBS and fixed in 4% formaldehyde for 30 min. Tissues were washed with 0.3% Triton-X 100 in PBS and blocked in 2% NGS for 2 h. Subsequently, tissues were stained with primary antibodies overnight at +4 °C. LipidTox staining was performed for fat bodies from early third instar larvae according to the manufacturer’s instructions. Protein synthesis was analyzed using the Click-iT® Plus OPP Protein Synthesis Assay Kit (Thermo Fisher) as described in (Sanchez et al, 2016). Early third instar larvae were dissected in M3 insect culture medium, and fat bodies were stained in the Click-iT OPP Reagent for 30 min at +25 °C. Samples were mounted in Vectashield (Vector Laboratories) mounting media with DAPI and imaged using the Leica SP8 and Leica SP8-upright microscopes. Images were further processed by the Fiji/ImageJ and Imaris software.

### Image quantifications

For lipid droplet analysis, cell borders were defined across multiple z-sections using the Imaris custom surface tool. Lipid staining was confined to the rendered cell by masking the lipid staining within the rendered cell surfaces. The surface detection tool was then applied to the masked lipid staining to calculate total lipid volume within each cell, and this value was subsequently normalized to the corresponding cell volume. Nuclear size quantification was performed by using a deep-learning-based nuclear segmentation algorithm, Stardist (Weigert et al, 2020). Nucleolar size quantifications were performed by manually selecting the region of interest (ROI) from DAPI-stained images showing the maximum nucleus size. Localization of nucleoli in the DAPI-stained nuclei of fat body cells was confirmed by anti-Fibrillarin immunostaining (Hernandez-Verdun, 2006). For fluorescence intensity measurements, background intensity was subtracted from the mean intensity of ROIs.

### Cell culture

HepG2 cells were cultured at 37 °C, 5% CO_2_ in a humidified incubator, in RPMI 1640 medium supplemented with 10% (vol/vol) fetal bovine serum, 1% penicillin–streptomycin, and 1% GlutaMAX. Stable SALL1-overexpressing in HepG2 cells (HEPG2-SALL1) were generated by transfection with human SALL1 ORF plasmid using jetPRIME reagent (Sartorius), followed by hygromycin B selection (0.3 mg/mL). HepG2 cells stably transfected with pCMV3-untagged vector were used as a control.

### Cell proliferation

Cell proliferation was measured with the sulforhodamine B (SRB) assay (Vichai and Kirtikara, 2006). Cells were seeded in triplicate at density 0.03 × 10^6^ cells/well in 48-well plates. The assay was performed on days 3 and 5 after seeding. Cells were fixed with 4% formaldehyde for 15 min at room temperature (RT) and stained with 0.4% SRB in 1% acetic acid for 30 min at RT. Unbound dye was removed by washing with 1% acetic acid, and the cell-bound dye was subsequently solubilized in 10 mM Tris base (pH 11). Blank wells without cells were processed identically to determine background staining. SRB solutions from individual wells were transferred in triplicate to 96-well plates, and absorbance was measured at 570 nm using a microplate reader (Victor Nivo, PerkinElmer). Mean background values were subtracted from the measurements to obtain corrected absorbance values used for statistical analysis.

### RNA extraction, qPCR, and mRNA sequencing

For RNA extraction from *Drosophila* samples, age-matched L1 larvae were collected and grown on standard laboratory food at equal density (30/vial). Fat body tissues of early third instar larvae were dissected, and RNA extraction was done using the Nucleospin RNA kit (Macherey-Nagel) according to the manufacturer’s instructions. For HepG2 cell RNA extraction, the cells were cultured in 6-well plates at ~70% confluence, then detached with 0.05% Trypsin-EDTA and washed with Dulbecco’s Phosphate Buffered Saline. RNA extraction was done using the Nucleospin RNA kit (Macherey-Nagel) according to the manufacturer’s instructions.

For qPCR, cDNA was synthesized using a RevertAid H Minus First Strand cDNA Synthesis Kit (Thermo Fisher) following the manufacturer’s protocol. qPCR was performed with a LightCycler 480 Real-Time PCR System (Roche) using Maxima SYBR Green qPCR Master Mix (Thermo Fisher).

For mRNA sequencing, libraries were generated using the TruSeq Stranded mRNA Library Prep Kit (Illumina) and subsequently sequenced on an Illumina NextSeq 500 to an average depth of 20 M reads per sample. The reads were mapped with TopHat to the *D. melanogaster* reference genome (FlyBase R6.10). The differential expression was quantified with the limma package (v.3.28.8) (Ritchie et al, 2015) implemented in R/Bioconductor with Benjamini–Hochberg correction for adjusting *P* values. Genes with an adjusted *P* value < 0.05 were differentially expressed (DE). Genes with very low counts (cpm <1 across more than one replicate per condition) were filtered. The raw sequencing data are deposited into the GEO repository (GSE305957). Differentially expressed gene lists can be found in Dataset EV1. Genes identified as differentially expressed were subjected to over-representation analysis (ORA) to identify significantly enriched pathways. ORA was performed using the clusterProfiler package (v.4.12.6) (Xu et al, 2024) in R/Bioconductor. Pathways with an adjusted *P* value < 0.05 with Benjamini–Hochberg correction were considered significantly enriched.

### ChIP-Seq

Browser Extensible Data (BED) files and control-normalized bigWig files of Salr (GSE257049), FoxO (GSE258971), SALL1 (GSE231113), FOXO1 (GSE170347), and XRP1 (GSE257114) were obtained from the ENCODE portal (https://www.encodeproject.org/) (The ENCODE Project Consortium, 2012). Peaks and coverage tracks were visualized with karyoploteR (v1.30.0) (Gel and Serra, 2017). Promotor regions were defined as 500 bp and 1 kb upstream to the transcription start site for *D. melanogaster* and *H. sapiens* data, respectively. Peaks were obtained from the ENCODE ChIP-seq uniform processing pipeline, in which each ChIP-seq dataset was processed by ENCODE using its corresponding input control. The reported optimal IDR thresholded peaks represent genomic regions where the ChIP signal significantly exceeds the matched input background across replicates.

### Statistical analysis

Statistical analyses were performed in GraphPad Prism 10 (GraphPad Software). For survival and pupariation rate analysis, the Log-rank test was used. For parametric data, an unpaired *t* test with Welch correction or one-way ANOVA with Tukey’s test with multiple comparison correction was used. The exact test, sample numbers, and *P* values for each experiment are indicated in the figure legends. All quantitative data are shown as the mean ± SD. 

## Supplementary information

Table EV1

Peer Review File

Expanded View Figures

## Data Availability

Source data are provided with this manuscript: BioStudies S-BSST3067. The RNA-seq datasets generated in this study are available from the following database: Gene Expression Omnibus: GSE305957. The source data of this paper are collected in the following database record: biostudies:S-SCDT-10_1038-S44318-026-00858-1.

## References

[CR1] Al-Baradie R, Yamada K, St Hilaire C, Chan W-M, Andrews C, McIntosh N, Nakano M, Martonyi EJ, Raymond WR, Okumura S et al (2002) Duane radial ray syndrome (Okihiro syndrome) maps to 20q13 and results from mutations in SALL4, a new member of the SAL family. Am J Hum Genet 71:1195–1199 12395297 10.1086/343821 PMC385096

[CR2] Baillon L, Germani F, Rockel C, Hilchenbach J, Basler K (2018) Xrp1 is a transcription factor required for cell competition-driven elimination of loser cells. Sci Rep 8:17712 30531963 10.1038/s41598-018-36277-4 PMC6286310

[CR3] Barrio R, De Celis JF, Bolshakov S, Kafatos FC (1999) Identification of regulatory regions driving the expression of the Drosophila spalt complex at different developmental stages. Dev Biol 215:33–47 10525348 10.1006/dbio.1999.9434

[CR4] Bartok O, Teesalu M, Ashwall-Fluss R, Pandey V, Hanan M, Rovenko BM, Poukkula M, Havula E, Moussaieff A, Vodala S et al (2015) The transcription factor Cabut coordinates energy metabolism and the circadian clock in response to sugar sensing. EMBO J 34:1538–1553 25916830 10.15252/embj.201591385 PMC4474529

[CR5] Bejarano F, Luque CM, Herranz H, Sorrosal G, Rafel N, Pham TT, Milán M (2008) A gain-of-function suppressor screen for genes involved in dorsal-ventral boundary formation in the Drosophila wing. Genetics 178:307–323 18202376 10.1534/genetics.107.081869 PMC2206080

[CR56] Bischof J, Björklund M, Furger E, Schertel C, Taipale J, Basler K (2013) A versatile platform for creating a comprehensive UAS-ORFeome library in Drosophila. Development 140:2434–2442 10.1242/dev.088757 23637332

[CR6] Blanco J, Cooper JC, Baker NE (2020) Roles of C/EBP class bZip proteins in the growth and cell competition of Rp (‘Minute’) mutants in Drosophila. eLife 9:e50535 31909714 10.7554/eLife.50535 PMC6946401

[CR7] Brown B, Mitra S, Roach FD, Vasudevan D, Ryoo HD (2021) The transcription factor Xrp1 is required for PERK-mediated antioxidant gene induction in Drosophila. eLife 10:e74047 34605405 10.7554/eLife.74047 PMC8514241

[CR8] Brunet A, Bonni A, Zigmond MJ, Lin MZ, Juo P, Hu LS, Anderson MJ, Arden KC, Blenis J, Greenberg ME (1999) Akt promotes cell survival by phosphorylating and inhibiting a forkhead transcription factor. Cell 96:857–868 10102273 10.1016/s0092-8674(00)80595-4

[CR57] Casper AL, Baxter K, Van Doren M (2011) no child left behind encodes a novel chromatin factor required for germline stem cell maintenance in males but not females. Development 138:3357–3366 10.1242/dev.067942 PMC3143560 21752937

[CR9] Chen C-C, Jeon S-M, Bhaskar PT, Nogueira V, Sundararajan D, Tonic I, Park Y, Hay N (2010) FoxOs inhibit mTORC1 and activate Akt by inducing the expression of Sestrin3 and Rictor. Dev Cell 18:592–604 20412774 10.1016/j.devcel.2010.03.008 PMC3031984

[CR10] De Celis JF, Barrio R (2009) Regulation and function of Spalt proteins during animal development. Int J Dev Biol 53:1385–1398 19247946 10.1387/ijdb.072408jd

[CR11] De Celis JF, Barrio R, Kafatos FC (1996) A gene complex acting downstream of dpp in Drosophila wing morphogenesis. Nature 381:421–424 8632798 10.1038/381421a0

[CR13] Gel B, Serra E (2017) karyoploteR: an R/Bioconductor package to plot customizable genomes displaying arbitrary data. Bioinformatics 33:3088–3090 28575171 10.1093/bioinformatics/btx346 PMC5870550

[CR14] Goul C, Peruzzo R, Zoncu R (2023) The molecular basis of nutrient sensing and signalling by mTORC1 in metabolism regulation and disease. Nat Rev Mol Cell Biol 24:857–875 37612414 10.1038/s41580-023-00641-8

[CR15] Hasygar K, Deniz O, Liu Y, Gullmets J, Hynynen R, Ruhanen H, Kokki K, Käkelä R, Hietakangas V (2021) Coordinated control of adiposity and growth by anti-anabolic kinase ERK7. EMBO Rep 22:e49602 33369866 10.15252/embr.201949602 PMC7857433

[CR16] Hernandez-Verdun D (2006) The nucleolus: a model for the organization of nuclear functions. Histochem Cell Biol 126:135–148 16835752 10.1007/s00418-006-0212-3

[CR17] Hietakangas V, Cohen SM (2007) Re-evaluating AKT regulation: role of TOR complex 2 in tissue growth. Genes Dev 21:632–637 17369395 10.1101/gad.416307 PMC1820936

[CR18] Hopkins T, Ragsdale C, Seo J (2025) Elevated ambient temperature reduces fat storage through the FoxO-mediated insulin signaling pathway. PLOS ONE 20:e0317971 40009607 10.1371/journal.pone.0317971 PMC11864546

[CR19] Huang H, Tindall DJ (2007) Dynamic FoxO transcription factors. J Cell Sci 120:2479–2487 17646672 10.1242/jcs.001222

[CR20] Isotani S, Hara K, Tokunaga C, Inoue H, Avruch J, Yonezawa K (1999) Immunopurified mammalian target of rapamycin phosphorylates and activates p70 S6 kinase α in vitro. J Biol Chem 274:34493–34498 10567431 10.1074/jbc.274.48.34493

[CR21] Kaade E, Mausbach S, Erps N, Sylvester M, Shakeri F, Jachimowicz RD, Gieselmann V, Thelen M (2024) Starvation-induced metabolic rewiring affects mTORC1 composition in vivo. Sci Rep 14:28296 39550382 10.1038/s41598-024-78873-7 PMC11569187

[CR22] Kim D-H, Sarbassov DD, Ali SM, King JE, Latek RR, Erdjument-Bromage H, Tempst P, Sabatini DM (2002) mTOR interacts with raptor to form a nutrient-sensitive complex that signals to the cell growth machinery. Cell 110:163–175 12150925 10.1016/s0092-8674(02)00808-5

[CR23] Klein S, Gastaldelli A, Yki-Järvinen H, Scherer PE (2022) Why does obesity cause diabetes? Cell Metab 34:11–20 34986330 10.1016/j.cmet.2021.12.012 PMC8740746

[CR24] Kohlhase J, Wischermann A, Reichenbach H, Froster U, Engel W (1998) Mutations in the SALL1 putative transcription factor gene cause Townes-Brocks syndrome. Nat Genet 18:81–83 9425907 10.1038/ng0198-81

[CR25] Kudron MM, Victorsen A, Gevirtzman L, Hillier LW, Fisher WW, Vafeados D, Kirkey M, Hammonds AS, Gersch J, Ammouri H et al (2018) The ModERN resource: genome-wide binding profiles for hundreds of Drosophila and *Caenorhabditis elegans* transcription factors. Genetics 208:937–949 29284660 10.1534/genetics.117.300657 PMC5844342

[CR26] Lee C-H, Kiparaki M, Blanco J, Folgado V, Ji Z, Kumar A, Rimesso G, Baker NE (2018) A regulatory response to ribosomal protein mutations controls translation, growth, and cell competition. Dev Cell 46:456–469.e4 30078730 10.1016/j.devcel.2018.07.003 PMC6261318

[CR27] Lee JH, Budanov AV, Park EJ, Birse R, Kim TE, Perkins GA, Ocorr K, Ellisman MH, Bodmer R, Bier E et al (2010) Sestrin as a feedback inhibitor of TOR that prevents age-related pathologies. Science 327:1223–1228 20203043 10.1126/science.1182228 PMC2866632

[CR28] Liu Y, Mattila J, Ventelä S, Yadav L, Zhang W, Lamichane N, Sundström J, Kauko O, Grénman R, Varjosalo M et al (2017) PWP1 mediates nutrient-dependent growth control through nucleolar regulation of ribosomal gene expression. Dev Cell 43:240–252.e5 29065309 10.1016/j.devcel.2017.09.022

[CR29] Malzer E, Szajewska-Skuta M, Dalton LE, Thomas SE, Hu N, Skaer H, Lomas DA, Crowther DC, Marciniak SJ (2013) Coordinate regulation of eIF2α phosphorylation by dPPP1R15 and dGCN2 is required during development. J Cell Sci 126:1406–1415 10.1242/jcs.117614 PMC3644141 23418347

[CR30] Mattila J, Kallijärvi J, Puig O (2008) RNAi screening for kinases and phosphatases identifies FoxO regulators. Proc Natl Acad Sci USA 105:14873–14878 18815370 10.1073/pnas.0803022105 PMC2567460

[CR31] Parmigiani A, Nourbakhsh A, Ding B, Wang W, Kim YC, Akopiants K, Guan K-L, Karin M, Budanov AV (2014) Sestrins inhibit mTORC1 kinase activation through the GATOR complex. Cell Rep 9:1281–1291 25457612 10.1016/j.celrep.2014.10.019 PMC4303546

[CR32] Port F, Starostecka M, Boutros M (2020) Multiplexed conditional genome editing with Cas12a in *Drosophila*. Proc Natl Acad Sci USA 117:22890–22899 32843348 10.1073/pnas.2004655117 PMC7502738

[CR33] Priest C, Tontonoz P (2019) Inter-organ cross-talk in metabolic syndrome. Nat Metab 1:1177–1188 32694672 10.1038/s42255-019-0145-5

[CR34] Puig O, Marr MT, Ruhf ML, Tjian R (2003) Control of cell number by *Drosophila* FOXO: downstream and feedback regulation of the insulin receptor pathway. Genes Dev 17:2006–2020 12893776 10.1101/gad.1098703 PMC196255

[CR35] Puig O, Mattila J (2011) Understanding forkhead box class O function: lessons from *Drosophila melanogaster*. Antioxid Redox Signal 14:635–647 20618068 10.1089/ars.2010.3407

[CR36] Recasens-Alvarez C, Alexandre C, Kirkpatrick J, Nojima H, Huels DJ, Snijders AP, Vincent J-P (2021) Ribosomopathy-associated mutations cause proteotoxic stress that is alleviated by TOR inhibition. Nat Cell Biol 23:127–135 33495632 10.1038/s41556-020-00626-1 PMC7116740

[CR37] Ritchie ME, Phipson B, Wu D, Hu Y, Law CW, Shi W, Smyth GK (2015) limma powers differential expression analyses for RNA-sequencing and microarray studies. Nucleic Acids Res 43:e47 25605792 10.1093/nar/gkv007 PMC4402510

[CR38] Rodriguez-Colman MJ, Dansen TB, Burgering Boudewijn MT (2024) FOXO transcription factors as mediators of stress adaptation. Nat Rev Mol Cell Biol 25:46–64 37710009 10.1038/s41580-023-00649-0

[CR39] Ryoo HD (2024) The integrated stress response in metabolic adaptation. J Biol Chem 300:107151 38462161 10.1016/j.jbc.2024.107151 PMC10998230

[CR40] Saito Y, Miyakawa S, Ando S, Kasano-Camones CI, Sumardika IW, Obayashi A, Morimoto A, Kurosawa A, Sakaguchi M, Ninomiya K et al (2025) SALL1 functions as a tumor suppressor in hepatocellular carcinoma cells. Biochem Biophys Res Commun 778:152385 40706249 10.1016/j.bbrc.2025.152385

[CR41] Sanchez CG, Teixeira FK, Czech B, Preall JB, Zamparini AL, Seifert JRK, Malone CD, Hannon GJ, Lehmann R (2016) Regulation of ribosome biogenesis and protein synthesis controls germline stem cell differentiation. Cell Stem Cell 18:276–290 26669894 10.1016/j.stem.2015.11.004 PMC4744108

[CR55] Sánchez J, Talamillo A, Lopitz-Otsoa F, Pérez C, Hjerpe R, Sutherland JD, Herboso L, Rodríguez MS, Barrio R (2010) Sumoylation Modulates the Activity of Spalt-like Proteins during Wing Development in Drosophila. J Biol Chem 285:25841–25849 10.1074/jbc.M110.124024 PMC2919146 20562097

[CR58] Schindelin J, Arganda-Carreras I, Frise E, Kaynig V, Longair M, Pietzsch T, Preibisch S, Rueden C, Saalfeld S, Schmid B et al (2012) Fiji: an open-source platform for biological-image analysis. Nat Methods 9:676–682 10.1038/nmeth.2019 PMC3855844 22743772

[CR42] Shi F, Li T, Liu Z, Qu K, Shi C, Li Y, Qin Q, Cheng L, Jin X, Yu T et al (2018) FOXO1: another avenue for treating digestive malignancy? Semin Cancer Biol 50:124–131 28965871 10.1016/j.semcancer.2017.09.009 PMC5874167

[CR43] Sturtevant MA, Biehs B, Marin E, Bier E (1997) The *spalt* gene links the A/P compartment boundary to a linear adult structure in the *Drosophila* wing. Development 124:21–32 9006064 10.1242/dev.124.1.21

[CR44] Teleman AA, Hietakangas V, Sayadian AC, Cohen SM (2008) Nutritional control of protein biosynthetic capacity by insulin via Myc in Drosophila. Cell Metab 7:21–32 18177722 10.1016/j.cmet.2007.11.010

[CR45] Texada MJ, Koyama T, Rewitz K (2020) Regulation of body size and growth control. Genetics 216:269–313 33023929 10.1534/genetics.120.303095 PMC7536854

[CR46] The ENCODE Project Consortium (2012) An integrated encyclopedia of DNA elements in the human genome. Nature 489:57–74. 22955616 10.1038/nature11247 PMC3439153

[CR47] The modECODE Consortium, Roy S, Ernst J, Kharchenko PV, Kheradpour P, Negre N, Eaton ML, Landolin JM, Bristow CA, Ma L et al (2010) Identification of functional elements and regulatory circuits by *Drosophila* modENCODE. Science 330:1787–1797 10.1126/science.1198374 PMC3192495 21177974

[CR48] Vichai V, Kirtikara K (2006) Sulforhodamine B colorimetric assay for cytotoxicity screening. Nat Protoc 1:1112–1116 17406391 10.1038/nprot.2006.179

[CR49] Wang L, Harris TE, Roth RA, Lawrence JC (2007) PRAS40 regulates mTORC1 kinase activity by functioning as a direct inhibitor of substrate binding. J Biol Chem 282:20036–20044 17510057 10.1074/jbc.M702376200

[CR50] Weigert M, Schmidt U, Haase R, Sugawara K, Myers G (2020) Star-convex polyhedra for 3D object detection and segmentation in microscopy. In: 2020 IEEE Winter Conference on Applications of Computer Vision (WACV). IEEE, Snowmass Village, CO, USA, pp 3655–3662

[CR51] Wolfson RL, Chantranupong L, Saxton RA, Shen K, Scaria SM, Cantor JR, Sabatini DM (2016) Sestrin2 is a leucine sensor for the mTORC1 pathway. Science 351:43–48 26449471 10.1126/science.aab2674 PMC4698017

[CR52] Xu S, Hu E, Cai Y, Xie Z, Luo X, Zhan L, Tang W, Wang Q, Liu B, Wang R et al (2024) Using clusterProfiler to characterize multiomics data. Nat Protoc 19:3292–3320 39019974 10.1038/s41596-024-01020-z

[CR53] Xu X, Gopalacharyulu P, Seppänen-Laakso T, Ruskeepää A-L, Aye CC, Carson BP, Mora S, Orešič M, Teleman AA (2012) Insulin signaling regulates fatty acid catabolism at the level of CoA activation. PLoS Genet 8:e1002478 22275878 10.1371/journal.pgen.1002478 PMC3261918

[CR54] Yu X-N, Chen H, Liu T-T, Wu J, Zhu J-M, Shen X-Z (2019) Targeting the mTOR regulatory network in hepatocellular carcinoma: are we making headway? Biochim Biophys Acta BBA - Rev Cancer 1871:379–391 10.1016/j.bbcan.2019.03.001 30951815

