# Supplementary material for: Spalt-related is an inhibitor of mTORC1-mediated growth activated by the integrated stress response
Source: EMBO J. 2026 Jul 11;45(16):5919–42. doi: 10.1038/s44318-026-00858-1 (PMC13482101; doi:10.1038/s44318-026-00858-1)
Supplement: Supplementary file 1 [file 44318_2026_858_MOESM2_ESM.pdf]

THE EMBO JOURNAL

Spalt-related is an inhibitor of mTORC1-mediated growth activated by the integrated stress response

Onur Deniz, Ying Liu, Tuuli Kirkinen, Krista Kokki, Kateryna Gaertner, Pau Clavell-Revelles, Jaakko Mattila, and Ville Hietakangas

Corresponding authors: Ville Hietakangas (ville.hietakangas@helsinki.fi)

Review Timeline:

Submission Date:	3rd Oct 25
Editorial Decision:	3rd Nov 25
Revision Received:	28th May 26
Editorial Decision:	18th Jun 26
Revision Received:	23rd Jun 26
Accepted:	2nd Jul 26

Editor: Ieva Gailite

Transaction Report:

(Note: With the exception of the correction of typographical or spelling errors that could be a source of ambiguity, letters and reports are not edited. Depending on transfer agreements, referee reports obtained elsewhere may or may not be included in this compilation. Referee reports are anonymous unless the Referee chooses to sign their reports.)

1st Editorial Decision 3rd Nov 2025

Dear Dr. Hietakangas,

Thank you for submitting your manuscript for consideration by the EMBO Journal. We have now received comments from a full set of reviewers, which are included below for your information.

As you will see, all reviewers find the study per se of interest and appreciate its contribution to the research field on a conceptual level. At the same time, while reviewer #2 is more positive, reviewers #1 and #3 indicate a number of substantive concerns regarding the experimental approach and the depth of insight that would need to be addressed before they can support publication here.

Based on the general interest expressed in these reports, I invite you to revise the manuscript in response to the comments by all reviewers. I think that it would be helpful to discuss the revision in more detail via email or phone/videoconferencing - please let me know which option you prefer.

We generally allow three months as standard revision time, which can be extended to six months in the case of major revisions. Should you foresee a problem in meeting this deadline, please let us know in advance to discuss an extension.

As a matter of policy, competing manuscripts published during this period will not negatively impact on our assessment of the conceptual advance presented by your study. However, please contact me as soon as possible upon publication of any related work to discuss the appropriate course of action.

When preparing your letter of response to the referees' comments, please bear in mind that this will form part of the Review Process File and will therefore be available online to the community. For more details on our Transparent Editorial Process, please visit our website: https://www.embopress.org/page/journal/14602075/authorguide#transparentprocess. Please also see the attached instructions for further guidelines on preparation of the revised manuscript.

Please feel free to contact me if you have any further questions regarding the revision. Thank you for the opportunity to consider your work for publication. I look forward to discussing your revision.

With best wishes,

Ieva

Ieva Gailite, PhD
Senior Scientific Editor
The EMBO Journal
Meyerhofstrasse 1
D-69117 Heidelberg
Tel: +4962218891309
i.gailite@embojournal.org

Instructions for preparing your revised manuscript:

Please make sure you upload a letter of response to the referees' comments together with the revised manuscript.

Please also check that the title and abstract of the manuscript are brief, yet explicit, even to non-specialists.

When assembling figures, please refer to our figure preparation guideline in order to ensure proper formatting and readability in print as well as on screen:
https://bit.ly/EMBOPressFigurePreparationGuideline
See also guidelines for figure legends: https://www.embopress.org/page/journal/14602075/authorguide#figureformat

At EMBO Press we ask authors to provide source data for the main manuscript figures. You will receive a separate email with instructions for providing source data with your revised manuscript, including how to upload and organize the files.

IMPORTANT: When you send the revision we will require
- a point-by-point response to the referees' comments, with a detailed description of the changes made (as a word file).
- a word file of the manuscript text.
- individual production quality figure files (one file per figure)
- a complete author checklist, which you can download from our author guidelines

(https://www.embopress.org/page/journal/14602075/authorguide).
- Expanded View files (replacing Supplementary Information)
Please see out instructions to authors
https://www.embopress.org/page/journal/14602075/authorguide#expandedview
- a Reagents and Tools Table as part of the Methods section, which can be downloaded from our author guidelines (https://www.embopress.org/page/journal/14602075/authorguide#structuredmethods)

Please remember: Digital image enhancement is acceptable practice, as long as it accurately represents the original data and conforms to community standards. If a figure has been subjected to significant electronic manipulation, this must be noted in the figure legend or in the 'Materials and Methods' section. The editors reserve the right to request original versions of figures and the original images that were used to assemble the figure.

Further information is available in our Guide For Authors: https://www.embopress.org/page/journal/14602075/authorguide

We realize that it is difficult to revise to a specific deadline. In the interest of protecting the conceptual advance provided by the work, we recommend a revision within 3 months (1st Feb 2026). Please discuss the revision progress ahead of this time with the editor if you require more time to complete the revisions.

Referee #1:

In this manuscript, the authors report the role of the Zn finger transcription factor, Salr, in Drosophila growth control. They specifically show that Salr is induced in response to starvation and the Integrated Stress Response (ISR), suppresses FoxO function, and contributes to the maintenance of protein synthesis and lipid storage in the larval fat body.

Overall, the study's conclusions are potentially interesting, but the data presented do not sufficiently support those claims. Among others, the authors rely on single RNAi lines for many experiments, leaving open the possibility of off-target effects. There is also concern as to whether the results presented here are consistent with some published data on starvation-induced gene expression profile changes. Resolving the discrepancies with the published data would be important. In addition, there are a number of points that could improve the clarity of the manuscript. Below is a list of specific points along these lines for the authors' consideration.

Major concerns

1. Knockdown experiments are mostly based on single RNAi lines, which leaves open the possibility of off-target effects. Salr knockdowns are nicely validated with Salr knockouts. But results with the knockdown of Xrp1, Raptor, FoxO, Akt require independent validation, either with independent RNAi lines or with classical mutant alleles.
2. Line120-129, Fig.3E: The authors examined S6 (RpS6) phosphorylation, using anti-phospho S6 (Cell Signaling) staining, as a readout for the mTORC1 pathway activity. S6 is also an ERK substrate. Does the anti-phospho-S6 (Cell Signaling) antibody specifically respond to mTORC1, or does it also detect ERK-induced phosphorylation? The authors should provide the relevant information about this antibody. If this antibody also responds to ERK activity, they need to provide additional assays to assess mTORC1 activity.
3. In Fig.1C, D, the authors describe Salr-mediated changes nuclear and nucleolar size. A reader could recognize the nuclei based on the provided DAPI labeling images, but it is unclear how the authors marked the nucleoli. The methods section simply states that the region of interest (ROI) was manually selected. Please add details regarding the nucleoli measurement.
4. Line 75-76. The authors write that Salr overexpression reduces cell size, but the actual Figure 1C doesn't show any cell size data. If possible, it would be nice to at least show the outline of the cells in Figure 1C, as they had done in Figure 1G.
5. FoxO and Salr ChIP-seq results are shown in Figure 4 and S4, S5. The results are from a public database. What tissues were those results generated from? If not from the larval fat body, can we apply that information to understand the roles of FoxO and Salr in that tissue, given that there is significant tissue-specific gene expression control? This is a major concern that needs to be addressed through additional validations.
6. FoxO and Salr have very similar ChIP seq patterns. How could one be certain that these are specific peaks, and not some non-specific hot spots? Are there IgG control tracks as negative controls?
7. What is known about the preferred binding sequences of FoxO and Salr? Do they have similar position weight matrices? Is that why they have similar ChIP-seq patterns in vivo?
8. In describing Fig.4E, the authors write that Salr loss of function increased overall FoxO levels. The authors show FoxO immunohistochemistry results, which is insufficient to claim an increase in FoxO levels. The authors should be mindful that nuclear translocation of a given transcription factor often generates stronger immunohistochemistry signals. To conclude that FoxO levels increase, the authors need to provide FoxO Western blots.
9. Line 161-163, Fig.5B: The authors find that the two different transcription factors show different induction dynamics, correlating

with the antagonizing role of Salr against FoxO. The authors could go beyond correlation and establish a causal relationship by examining the time course of FoxO induction in the Salr loss-of-function background.
10. The authors examined the role of Xrp1 during larval starvation. Have authors ever checked the expression of Xrp1 in the starved larval fat body? Is the protein induced?
11. Figure 5D: The authors show that Xrp1 overexpression causes severe hypotrophy and larval lethality. Some of these phenotypes are likely due to excessive Xrp1 expression, which is known to kill cells in other tissues (e.g., PMID 31909714). If PPP1R15 RNAi induces endogenous Xrp1, any Xrp1 overexpression phenotype not seen with PPP1R15 RNAi could be an overexpression artifact.
12. Starvation-induced gene expression changes related to the ISR had been documented in a previous study (Kosakamoto et al., Nat Metabolism PMID 35879463). After a quick glance, I don't think I see Salr among the starvation-induced genes in that study. Are those published datasets consistent with the main conclusions of the current study (is Salr and its target genes induced by starvation)? If not, what is the reason? I am not an author of the Kosakamoto study, and I am agnostic about who is right or wrong here. But it would be important to reconcile any major differences with the existing published results.
13. Corroborating evidence for the ISR-Xrp1-Salr axis would strengthen this work. As it is, Xrp1 RNAi is the only condition that the authors use to block the ISR. How about blocking upstream eIF2a kinases (like Gcn2 or Perk).
Minor comments
1. The bar graphs do not indicate the individual data points. Please show individual data points as dots on top of the bar graphs.
2. Salr knockdown and knockout experiments are shown throughout the manuscript. It would be good to show the extent of Salr expression change through qPCR or Western Blot.
3. Fig.1E clearly indicates the color of the labeled protein, but most other panels do not indicate which color is which. Please indicate what the colors in each panel represent.
4. It would be nice to show whole fat body tissue images (perhaps as a Supplemental figure) to determine if DPP, lipid volume, Salr-GFP, p-S6 and FoxO signal changes are uniform or region specific.
5. Line 54: The authors refer to Townes Brocks Syndrome (SALL1). Is this the correct abbreviation?
6. Figure 1B shows delayed pupariation caused by Salr overexpression. What about Salr loss of function? Do the larvae show accelerated development?
7. Fig.2D,E: The datasets are currently arranged from high to low, but this makes it difficult to compare corresponding data points between the two sample sets due to the different order. It might be better to rearrange the data according to the analysis conditions.
8. Line 127: What is LOF?
9. Fig.1E, Fig.4F: What is F/O?
10. Fig.5A: The Salr-GFP signal is unclear in the Akt-I experiment because of a high background.
11. Line 238: The Methods section describes a UAS-Luciferase RNAi, but I could not find experiments using this in the main text.
12. Line 246-247: There is some sequence information here. Is this the guide RNA sequence? Why is there only one sequence when four gRNAs were designed?
13. Line 262: Correct "Foxo" spelling.

Referee #2:

This is a very interesting and solid study that identifies Salr as a transcription factor that acts similarly to FOXO - it is activated upon starvation and promotes expression of catabolic genes, thereby inhibiting mTORC1 activation and regulating animal growth. I found it exciting that the human homologs of Salr also bind the same genomic loci as human FOXO, indicating that the metabolic function of the human Salr homologs should be studied in the future. I think this study will be of interest to a wide range of scientists studying metabolism, stress, growth and physiology. One additional strength of this study is that it is very solid - congruent results are shown using independent manipulations (e.g. CRISPR/Cas and knockdowns) and converse results are obtained with gain-of-function versus loss-of-function experiments.

I have only minor comments:
1. It would be helpful to provide a supplemental table to the reader with the top up-regulated and down-regulated genes (with fold changes) upon Salr GOF and LOF in the fat body.

2. Typos / grammar:
line 62: "Salr targets include components of the mTORC1, such as..."
line 44: "and inhibit mTOR activity by promoting the expression its negative upstream regulator Sestrin"
line 49: "Despite these insights, we still lack in full understanding of how..."

3. Fig 1C - where is the nucleolus? Is it the large area in the center of the nucleus that stains poorly for DAPI? It would be good to describe it for the reader because it has an unusual morphology compared to other cells.

4. Fig 3E and Materials & Methods - the pS6 staining looks like a useful readout of mTORC1 activity. It would be helpful to indicate the catalog number of the pS6 antibody from Cell Signaling that was used in this study.

5. Fig 4A - from which study are the FOXO target genes taken from, which are used to compare to the Salr targets here? This should be cited in the results section. Same for the FOXO ChIP data (Fig. 4B).

6. Line 153, the authors conclude " In sum, Salr binds to common genomic regions WITH FoxO and functionally antagonizes its nuclear localization."
Do the authors mean
"In sum, Salr binds to common genomic regions AS FoxO and functionally antagonizes its nuclear localization" ? Because the ChIP data indicates they bind similar regions, but it does not mean that they bind together. Indeed, the fact that nuclear Salr causes FOXO to leave the nucleus would suggest they don't bind at the same time, but take turns binding the same regions?

7. Fig 5C - knockdown of PPP1R15 causes increased levels of Salr, however they are not nuclear as in the case of starvation (Fig. 5B). Does this mean that Salr levels and localization are independently regulated by different inputs?

8. One mechanism how starvation induces the ISR is via activation of GCN2. Does GCN2 knockdown reduce Salr activation upon starvation? What about PERK knockdown? This would delineate a bit better how Salr expression is activated downstream of nutrient stress. Also, is it sugar starvation or amino acid starvation that is inducing Salr expression?

Referee #3:

This manuscript presents a mechanistically intriguing study describing how the transcription factor Spalt-related (Salr) functions as an integrated stress response (ISR)-activated inhibitor of mTORC1-driven growth in Drosophila fat body. The authors also describe a potentially novel regulatory mechanism by which Salr antagonizes Foxo transcription factor activity to control expression of shared catabolic target genes during fasting. Through transcriptomics, genetics, and analysis of publicly available ChIP-seq data, the authors' show that Salr also directly induces Sestrin, PRAS40, and REPTOR to repress mTORC1, thus restraining cellular growth and metabolism. The ISR-Xrp1-Salr axis, coupled with Foxo activity, provides clues linking nutrient status and nutrient stress duration to transcriptional control of cell growth and metabolism.

The manuscript is conceptually (and broadly) interesting, is clearly written, well organized, and the data do support some of the over-arching conclusions presented in the text. However, several of the key mechanistic claims, especially related to temporal molecular integration of Salr-mTORC1 and Salr-Foxo interactions with nutrient status (fasting), are based on indirect or heterologous evidence (described below), dampening overall enthusiasm.

Major concerns:

1. The authors show that Salr can influence Foxo cellular localization. However, the molecular basis of this Salr-Foxo transcription factor antagonism remains unclear (is it through Salr-regulation of insulin/Akt and/or other pathways, through changes in promoter co-occupancy, through changes in physical interactions with other protein complexes that control gene expression, etc?). Foxo generally acts as an activator of gene expression during fasting, and the authors propose that Salr is subsequently activated through the integrated stress response and antagonizes this Foxo function, while also inducing (sustaining) gene expression of shared targets during prolonged fasting. Although this model conceptually links Salr, the integrated stress response, and Foxo, the manuscript lacks direct genetic or molecular evidence to demonstrate how these factors (especially the antagonism of two transcription factors) functionally coordinate to temporally regulate gene expression during fasting. This is a critical gap that is essential to support the central mechanistic framework of the study.

The discussion extends the Salr-Foxo paradigm to human SALL1/FOXO1 and tumor suppression in pathological states based primarily on ChIP-seq co-occupancy. However, without any functional data in mammalian cells, these conclusions should be tempered.

2. Similarly to above, the authors link Salr function to mTORC1 activity, but the relationship between these molecular events and the physiological state (nutrient status) of the animal remains unclear. In particular, it is not well explained how Salr activation during fasting is functionally connected to the suppression of mTORC1 activity. Clarifying this temporal and physiological relationship would significantly strengthen the biological relevance of the proposed model.

3. The statement "Through systematic analysis of transcription factors, we discovered a novel growth inhibitory role for Spalt-related (Salr) ..." is central to the paper. However, the screening strategy is not clearly described. More detail is needed to know how the transcription factors were selected, and how Salr was identified as a key hit.

4. In the sentence "In addition to systemic growth regulation, Salr overexpression in fat body clones (Tubulin [flipout] Gal4) strongly reduced cell size demonstrating cell autonomous function (Figure 1C).", the evidence for reduced cell size is not entirely clear from the presented image. While the Salr-overexpressing cells are visible, the size of the surrounding fat body cells is less well defined, making it difficult to fully appreciate the magnitude of the size difference. It would strengthen the conclusion if the

authors could provide clearer comparative visualization or quantitative analysis to more convincingly demonstrate this effect.

5. The authors show that fat body-specific Salr overexpression reduces whole larval TAG levels (Figure 1I). Given this clear phenotype, it would be important and informative to also examine whether Salr knockdown or knockout leads to increased TAG levels.

6. In Figure 2A the authors use a Salr-GFP reporter and show increased Salr expression after 5 h fasting, whereas in Figure 5 they extend fasting to 16 h to argue that Salr activation is delayed relative to Foxo. Given that Salr activation is already detectable at 5 h, could the authors clarify whether Foxo is antagonized by Salr at this earlier time point? It would strengthen the temporal model to include measurements of Salr levels and Foxo nuclear/cytoplasmic ratios at 5 h whether early Salr induction measurably reduces Foxo nuclear localization.

6. In Figures 2 and S2, the authors propose that Salr activation during fasting plays a key role in controlling catabolic gene expression during nutrient deprivation. However, there is no direct evidence demonstrating whether Salr knockdown during nutrient deprivation affects the fasting-dependent induction of these catabolic genes. Including such data would be critical to support the conclusion that Salr is required for catabolic gene activation in response to changes in nutrient status.

7. The authors conclude that Salr inhibits growth by restricting mTORC1 activity, largely based on changes in nuclear and nucleolar size. It would be helpful to compliment these measurements with other growth-related parameters (e.g., larval size, weight, or developmental timing) to substantiate the claim that Salr regulates larval growth through inhibiting mTORC1 activity.

Minor comments:

1. The authors used the CRISPR/Cas12a system to knock out Salr specifically in the fat body, which is an appropriate and effective strategy for generating targeted gene deletions. However, no direct evidence is provided to confirm that Salr was successfully knocked out in the targeted tissue.

2. The manuscript refers to Figure S1C, but this panel does not appear to be included in the Supplementary Figure 1.

3. In Figure 5A, the nuclei in AKT-i cells appear noticeably smaller than those in the control group. Please check whether the magnification or scaling between the two groups is consistent.

4. It appears the Y-axis is mislabeled in Figure 1C (third panel).

Referee #1:

In this manuscript, the authors report the role of the Zn finger transcription factor, Salr, in Drosophila growth control. They specifically show that Salr is induced in response to starvation and the Integrated Stress Response (ISR), suppresses FoxO function, and contributes to the maintenance of protein synthesis and lipid storage in the larval fat body.

Overall, the study's conclusions are potentially interesting, but the data presented do not sufficiently support those claims. Among others, the authors rely on single RNAi lines for many experiments, leaving open the possibility of off-target effects. There is also concern as to whether the results presented here are consistent with some published data on starvation-induced gene expression profile changes. Resolving the discrepancies with the published data would be important. In addition, there are a number of points that could improve the clarity of the manuscript. Below is a list of specific points along these lines for the authors' consideration.

Thank you for the thorough work and constructive suggestions to improve our study.

<u>Major concerns</u>

1. Knockdown experiments are mostly based on single RNAi lines, which leaves open the possibility of off-target effects. Salr knockdowns are nicely validated with Salr knockouts. But results with the knockdown of Xrp1, Raptor, FoxO, Akt require independent validation, either with independent RNAi lines or with classical mutant alleles.

The Reviewer raises the concern that the reported phenotypes may reflect off-target effects of RNAi. We have now performed additional experiments to mitigate these concerns.

1. *The finding that mTORC1 inhibition (Raptor RNAi) suppresses the overgrowth of Salr knockout is now confirmed by Sestrin overexpression. It suppresses mTORC1 activity (new data in **Figure EV3H**) and phenocopies the suppression of Raptor RNAi (new data in **Figure EV3I**).*
2. *The finding that inhibition of insulin-AKT signaling (AKT RNAi) activates FoxO, but not Salr, is now verified by knockdown of InR (new data in **Figure EV4F**).*
3. *Foxo RNAi was not included in the original manuscript, but was used in the revision. We have used two independent RNAi lines, along with overexpression (new data in **Figure EV4C**).*
4. *The finding that Xrp1 depletion prevents Salr upregulation during prolonged fasting has now been replicated by an independent Xrp1 RNAi line (new data in **Figure EV6D**).*

2. Line120-129, Fig.3E: The authors examined S6 (RpS6) phosphorylation, using anti-phospho S6 (Cell Signaling) staining, as a readout for the mTORC1 pathway activity. S6 is also an ERK substrate. Does the anti-phospho-S6 (Cell Signaling) antibody specifically respond to mTORC1, or does it also detect ERK-induced phosphorylation? The authors should provide the relevant information about this antibody. If this antibody also responds to ERK activity, they need to provide additional assays to assess mTORC1 activity.

The Reviewer raises the concern about the reliability of the conclusion of Salr-mediated regulation of mTORC1. To mitigate the concern, we have now done the following revisions:

1. *We provide the specific information about the antibody used to detect the phosphorylation of S6 (Ser235/236) in the reagents and tools table. The Reviewer is right that in mammalian HEK293 cells, this phosphorylation has been shown to display minor sensitivity to MEK inhibitor U0126, in addition the major inhibition by mTORC1 inhibitor Rapamycin (Roux et al., 2007). Therefore, additional controls are indeed warranted.*
2. *To test the pS6 reactivity to mTORC1 in the fat body, we now show that the pS6 signal reacts strongly to genetic manipulation of mTORC1, being strongly inhibited by Raptor knockdown and activated by TSC1 knockdown (new data in **Figure EV3E**).*
3. *We also use an independent readout for mTORC1 activity, PWP1 nucleolar localization,(Liu et al., 2017). Salr knockout tissue shows strong accumulation of nucleolar PWP1 (new data in **Figure EV3G**), similar to TSC1 knockdown (new data in **Figure EV3F**), known to activate mTORC1.*

*In addition to these points, we wish to highlight that our conclusions of Salr-mediated regulation of mTORC1, is also based on gene expression changes of known mTORC1 regulators (such as Sestrin), along with their genetic analysis (e.g. new **Figures EV3H** & **EV3I**). Collectively, we think that our conclusion on inhibition of mTORC1 activity by Salr is now sufficiently supported by experimental evidence.*

3. In Fig.1C, D, the authors describe Salr-mediated changes nuclear and nucleolar size. A reader could recognize the nuclei based on the provided DAPI labeling images, but it is unclear how the authors marked the nucleoli. The methods section simply states that the region of interest (ROI) was manually selected. Please add details regarding the nucleoli measurement.

*The Reviewer asks about the identification of the nucleolus in order to quantify its size. We have now added a control with immunofluorescence staining of Fibrillarin, a well-established nucleolar marker (Ochs et al., 1985) (new data in **Figure EV1B**). This data shows that the Fibrillarin-positive nucleolar area increases upon Salr knockdown, consistent with our conclusion. The Fibrillarin-positive area corresponds to the large DAPI-negative area in the*

middle of the DAPI-positive nucleus, supporting the conclusion that nucleolar size of fat body cells can be estimated based on the DAPI staining.

4. Line 75-76. The authors write that Salr overexpression reduces cell size, but the actual Figure 1C doesn't show any cell size data. If possible, it would be nice to at least show the outline of the cells in Figure 1C, as they had done in Figure 1G.

The Reviewer requests direct measurement of cell size, which we have now performed, confirming the original conclusion (new data in ***Figure EV1A****).*

5. FoxO and Salr ChIP-seq results are shown in Figure 4 and S4, S5. The results are from a public database. What tissues were those results generated from? If not from the larval fat body, can we apply that information to understand the roles of FoxO and Salr in that tissue, given that there is significant tissue-specific gene expression control? This is a major concern that needs to be addressed through additional validations.

The Reviewer raises a concern about the tissue specificity of the Salr and FoxO ChIPseq data. The data originates from a whole animal (Salr: prepupa; FoxO: wandering 3rd instar larva) and thus lacks cell type specificity. We acknowledge the concern that the direct target genes of Salr and FoxO may reflect tissue-specific binding preference. We have adjusted the Results section to more accurately align with the data: "Many of the genes in the Salr regulated pathways in the fat body, including autophagy, lipid catabolism and mitochondrial ribosome biogenesis, were indeed direct targets in the full body ChIP-Seq analysis."

*We wish to emphasize that our study also includes another set of ChIP-seq data, originating from cultured human HepG2 cells (derived from liver which most closely functionally corresponds insect fat body). This data supports the conclusion that the orthologs of Salr and FoxO bind to overlapping genomic loci in the same cell type. Finally, to further confirm the function of fat body FoxO on relevant gene regulation, we now show that FoxO activates sestrin expression in the fat body (****Figure EV4C****), similar to the observed regulation by Salr.*

6. FoxO and Salr have very similar ChIP seq patterns. How could one be certain that these are specific peaks, and not some non-specific hot spots? Are there IgG control tracks as negative controls?

The Reviewer raises the concern that the similarity of Salr and Foxo ChIPseq patterns reflects non-specific hot spots. The data displayed in the manuscript includes subtraction of the IgG control signal, thus reflecting the specific enrichments obtained with anti-Salr and anti-FoxO antibodies. We have now specified this in the Materials and Methods section (lines 368-371). As

further confirmation, we provide below an image including the IgG control lane together with FoxO and Salr, demonstrating the specificity.

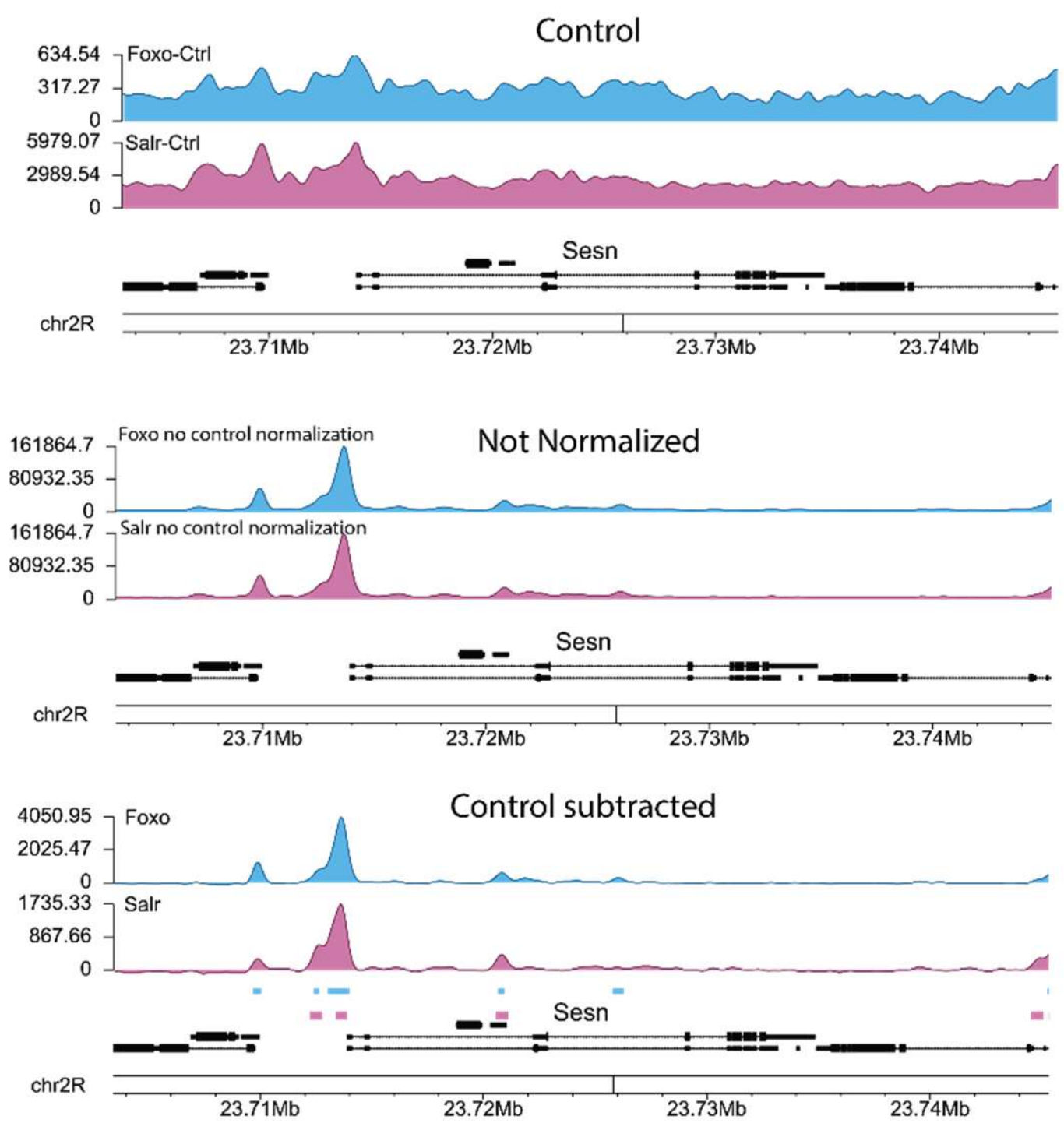

7. What is known about the preferred binding sequences of FoxO and Salr? Do they have similar position weight matrices? Is that why they have similar ChIP-seq patterns in vivo?

The Reviewer asks about the possible similarity of FoxO and Salr binding sequences. The binding motifs of FoxO and Salr have indeed previously been studied. FoxO genomic binding occurs at sites with repeats of T-rich DAF-16 family-member Binding Elements (DBEs). In Orengo et al. (Orengo et al., 2017) the analysis of 105 DBE sites experimentally detected in the five Drosophila species led to a Drosophila DBE consensus sequence: 5'-TTDTTKNB (D=A/G/T; K=G/T; N= any; B=C/G/T). For Drosophila Salr the best characterized example is the binding motif TATGAAAT in the s15 chorion gene (Barrio et al., 1996). This somewhat aligns with findings in mammals showing that mouse SALL1, SALL3 and SALL4 bind preferentially AT rich regions containing AATA tetranucleotides (Ru et al., 2022).

In conclusion, the known genomic binding sites of FoxO and Salr are not obviously similar. Properly addressing the precise binding specificities of FoxO and Salr in the overlapping genomic regions will require high-resolution binding-site determination (e.g. CUT&Tag) and in vitro follow-up experiments using EMSA. Therefore, it is beyond the scope of the current study.

8. In describing Fig.4E, the authors write that Salr loss of function increased overall FoxO levels.

The authors show FoxO immunohistochemistry results, which is insufficient to claim an increase in FoxO levels. The authors should be mindful that nuclear translocation of a given transcription factor often generates stronger immunohistochemistry signals. To conclude that FoxO levels increase, the authors need to provide FoxO Western blots.

The Reviewer points out the uncertainties related to immunofluorescence data in making conclusions about total protein levels. We share these concerns and as the potential regulation of total FoxO levels by Salr is not a central point of this study, we have opted to modify our conclusions, focusing our analyses on the nuclear-cytoplasmic ratios of FoxO.

9. Line 161-163, Fig.5B: The authors find that the two different transcription factors show different induction dynamics, correlating with the antagonizing role of Salr against FoxO. The authors could go beyond correlation and establish a causal relationship by examining the time course of FoxO induction in the Salr loss-of-function background.

*The Reviewer points out the importance of better understanding the interplay between FoxO and Salr and suggests an experiment to analyze FoxO activity kinetics in the presence vs. absence of Salr. We have now performed the suggested experiment. The experiment shows that the attenuation of nuclear FoxO localization upon prolonged fasting (16 h) is hampered in Salr loss-of-function tissue. This provides causal evidence for the role of Salr in the attenuation of FoxO upon prolonged fasting, strengthening the main conclusion of the study. The new data is now included as **Figure 5C**.*

10. The authors examined the role of Xrp1 during larval starvation. Have authors ever checked the expression of Xrp1 in the starved larval fat body? Is the protein induced?

*The Reviewer asks about the regulation of Xrp1 levels upon starvation. We have now analyzed this and observed that Xrp1 levels are indeed elevated upon fasting. Consistent with the model that Xrp1 serves as an upstream regulator of Salr, we observed that Xrp1 expression is rapidly elevated by fasting (up at 2 h and 5 h time points) and is attenuated upon prolonged fasting (16 h). This finding is consistent with a model that Xrp1 displays a transient activation, and activates Salr expression, which persists upon prolonged fasting. We have now added this new data into **Figure 6C** and **Figure EV6C** and revised the conclusions accordingly.*

11. Figure 5D: The authors show that Xrp1 overexpression causes severe hypotrophy and larval lethality. Some of these phenotypes are likely due to excessive Xrp1 expression, which is known to kill cells in other tissues (e.g., PMID 31909714). If PPP1R15 RNAi induces endogenous Xrp1,

any Xrp1 overexpression phenotype not seen with PPP1R15 RNAi could be an overexpression artifact.

The Reviewer points out the strong overexpression phenotype of Xrp1 and raises the concern of an "overexpression artifact". We share these concerns. Overexpression is a commonly used experimental manipulation, which has potential to reveal relevant information about the gene studied. Yet, it is certainly prone to producing phenotypes that are stronger than those observed at a physiological range. Our view is that overexpression can be used as a component of the experimental toolkit, as long as its limitations are understood and the interpretation of results is done in a thoughtful way that takes into account complementary evidence. In the case of Xrp1 overexpression, the key observation valuable for this study is the elevated expression Salr. This data fully aligns with the other evidence obtained, most notably the Xrp1 loss-of-function data (impaired activation of Salr). Therefore, we think that Xrp1 overexpression brings additional value to our study. To reduce the risk of readers over-interpreting the growth/lethality phenotypes of Xrp1 overexpression, we have added a cautionary note to the manuscript: "Overexpression of Xrp1 in the fat body led to a dramatic growth inhibition and larval lethality (not shown), possibly due to its known effects in cell survival (Blanco, Cooper and Baker, 2020). Even transient overexpression of Xrp1 led to substantial fat body hypotrophy, and markedly increased Salr expression (Figure 6B)."

12. Starvation-induced gene expression changes related to the ISR had been documented in a previous study (Kosakamoto et al., Nat Metabolism PMID 35879463). After a quick glance, I don't think I see Salr among the starvation-induced genes in that study. Are those published datasets consistent with the main conclusions of the current study (is Salr and its target genes induced by starvation)? If not, what is the reason? I am not an author of the Kosakamoto study, and I am agnostic about who is right or wrong here. But it would be important to reconcile any major differences with the existing published results.

The Reviewer brings up potential differences in gene expression data between our dataset and that of Kosakamoto et al. We have looked into this and noted that the experimental conditions between the studies are different. Most notably, we have used full nutrient deprivation, while Kosakamoto uses 4% yeast extract as the starvation condition. We have opted not to include comparisons between gene expression profiles under different starvation conditions, as it would unnecessarily expand the volume of the manuscript and deviate the focus from its primary scope. We have now specified our experimental conditions: "…of fully nutrient deprived larvae" (Line 296).

13. Corroborating evidence for the ISR-Xrp1-Salr axis would strengthen this work. As it is, Xrp1

RNAi is the only condition that the authors use to block the ISR. How about blocking upstream eIF2a kinases (like Gcn2 or Perk).

*The Reviewer suggests the possibility of knocking down Gcn2 and Perk as a parallel strategy to study the involvement of ISR in Salr regulation. We have performed these experiments and show that knockdown of both ISR kinases suppress Salr accumulation upon prolonged fasting (new data included as **Figure EV6A**). This data, together with the data on PPP1R15 knockdown is consistent with the conclusion that the ISR pathway regulates Salr expression.*

<u>Minor comments</u>

14. The bar graphs do not indicate the individual data points. Please show individual data points as dots on top of the bar graphs.

Corrected as suggested

15. Salr knockdown and knockout experiments are shown throughout the manuscript. It would be good to show the extent of Salr expression change through qPCR or Western Blot.

*We have confirmed the fat body-specific Salr knockout by monitoring the levels of endogenously tagged Salr-GFP fusion protein. These results are now in **Figure EV1E**.*

16. Fig.1E clearly indicates the color of the labeled protein, but most other panels do not indicate which color is which. Please indicate what the colors in each panel represent.

As suggested, we have now indicated the colors of labeled proteins in the figure panels.

17. It would be nice to show whole fat body tissue images (perhaps as a Supplemental figure) to determine if DPP, lipid volume, Salr-GFP, p-S6 and FoxO signal changes are uniform or region specific.

We have used close-up images of the fat body to achieve sufficient resolution, but always paid attention that the images recorded represent phenotypes broadly present in the tissue. Please note that our conclusions are based on quantifications of multiple images, not only the representative image displayed in the Figure. We opt not to include whole fat body images, given the extent of work required to replicate all of our experiments for this purpose. All the source data (including fat body images) is made available according to the Journal's policies.

18. Line 54: The authors refer to Townes Brocks Syndrome (SALL1). Is this the correct abbreviation?

In parentheses is the gene responsible for the syndrome. For the Okihiro Syndrome the linked gene was left out, hence the confusion. The full sentence is now corrected to: "Mutations in two

members of the human Sal-like (SALL) transcription factor family cause syndromes with impaired organogenesis, namely Townes Brocks Syndrome (SALL1) and Okihiro Syndrome (SALL4)".

19. Figure 1B shows delayed pupariation caused by Salr overexpression. What about Salr loss of function? Do the larvae show accelerated development?

*As requested, we have performed a pupariation kinetics experiment in the Salr LOF condition. Salr LOF does not cause noticeably accelerated development. However, the animals pupariate larger than controls, which is consistent with our other results demonstrating that Salr restricts fat body growth. These new data is included in **Figure 1E** and **Figure EV1F**.*

20. Fig.2D,E: The datasets are currently arranged from high to low, but this makes it difficult to compare corresponding data points between the two sample sets due to the different order. It might be better to rearrange the data according to the analysis conditions.

As suggested, we have rearranged the datasets in the plots to match the order of the analysis conditions.

21. Line 127: What is LOF?

We have explained the abbreviation (loss-of-function) where it first occurred in the text.

22. Fig.1E, Fig.4F: What is F/O?
F/O refers to the term 'flipout' clones. We have changed the abbreviation F/O in Figures 1C, G, I and 4F to 'flipout' to avoid confusion.

23. Fig.5A: The Salr-GFP signal is unclear in the Akt-I experiment because of a high background.
We have now changed the representative image to one with less background. The conclusion is based on the quantification.

24. Line 238: The Methods section describes a UAS-Luciferase RNAi, but I could not find experiments using this in the main text.

UAS-Luciferase RNAi refers to the Trip RNAi collection control line used throughout the study in RNAi experiments. We have clarified the use controls in the reagents and tools table.

25. Line 246-247: There is some sequence information here. Is this the guide RNA sequence? Why is there only one sequence when four gRNAs were designed?

The provided sequence contains all four gRNAs separated by spacer sequences. This is the sequence cloned into the pCFD8 plasmid, allowing expression of the guide RNAs. We have now included the gRNA sequence information in the methods and reagents table. The exact target sites of the gRNAs to the Salr gene are illustrated in Figure EV1D.

26. Line 262: Correct "Foxo" spelling.

Done

Referee #2:

This is a very interesting and solid study that identifies Salr as a transcription factor that acts similarly to FOXO - it is activated upon starvation and promotes expression of catabolic genes, thereby inhibiting mTORC1 activation and regulating animal growth. I found it exciting that the human homologs of Salr also bind the same genomic loci as human FOXO, indicating that the metabolic function of the human Salr homologs should be studied in the future. I think this study will be of interest to a wide range of scientists studying metabolism, stress, growth and physiology. One additional strength of this study is that it is very solid - congruent results are shown using independent manipulations (e.g. CRISPR/Cas and knockdowns) and converse results are obtained with gain-of-function versus loss-of-function experiments.
Thank you for your assessment and recognizing the potential for broad interest as well as the experimental rigor of our study.

I have only minor comments:
27. It would be helpful to provide a supplemental table to the reader with the top up-regulated and down-regulated genes (with fold changes) upon Salr GOF and LOF in the fat body.

As requested, we have added this data as ***Table S1****.*

28. Typos / grammar:
line 62: "Salr targets include components of the mTORC1, such as..."
line 44: "and inhibit mTOR activity by promoting the expression its negative upstream regulator Sestrin"
line 49: "Despite these insights, we still lack in full understanding of how..."

We have corrected these mistakes.

29. Fig 1C - where is the nucleolus? Is it the large area in the center of the nucleus that stains poorly for DAPI? It would be good to describe it for the reader because it has an unusual morphology compared to other cells.

To address this, we have added additional controls using anti-Fibrillarin staining, a well-established nucleolar marker (Ochs et al., 1985). This confirms that the DAPI deficient area in the fat body nucleus is indeed the nucleolus. This new data is included in ***Figure EV1B*** *and described in the Materials and Methods section.*

30. Fig 3E and Materials & Methods - the pS6 staining looks like a useful readout of mTORC1

activity. It would be helpful to indicate the catalog number of the pS6 antibody from Cell Signaling that was used in this study.

As requested, we have added this information to the methods and reagents table. We have also added more controls for the mTORC1 analysis (see response #2 to Reviewer 1).

31. Fig 4A - from which study are the FOXO target genes taken from, which are used to compare to the Salr targets here? This should be cited in the results section. Same for the FOXO ChIP data (Fig. 4B).

We have added the citations, as requested.

32. Line 153, the authors conclude " In sum, Salr binds to common genomic regions WITH FoxO and functionally antagonizes its nuclear localization."
Do the authors mean
"In sum, Salr binds to common genomic regions AS FoxO and functionally antagonizes its nuclear localization" ? Because the ChIP data indicates they bind similar regions, but it does not mean that they bind together. Indeed, the fact that nuclear Salr causes FOXO to leave the nucleus would suggest they don't bind at the same time, but take turns binding the same regions?

Thank you for pointing this out, indeed, we mean as the reviewer suggests. We have corrected it as: "…Salr binds to genomic regions that overlap with those of FoxO".

33. Fig 5C - knockdown of PPP1R15 causes increased levels of Salr, however they are not nuclear as in the case of starvation (Fig. 5B). Does this mean that Salr levels and localization are independently regulated by different inputs?

Our measurements of Salr levels have been performed from the nucleus, and knockdown of PPP1R15 leads to higher nuclear Salr intensity than in controls. The question of potential additional regulation of Salr subcellular localization is an intriguing question for future studies.

34. One mechanism how starvation induces the ISR is via activation of GCN2. Does GCN2 knockdown reduce Salr activation upon starvation? What about PERK knockdown? This would delineate a bit better how Salr expression is activated downstream of nutrient stress. Also, is it sugar starvation or amino acid starvation that is inducing Salr expression?

As requested, we have knocked down both GCN2 and PERK. Based on our data, knockdown of both of these ISR kinases inhibits Salr accumulation upon prolonged fasting (new data included as ***Figure EV6A****). This data strengthens the conclusion that the ISR pathway regulates Salr*

expression during fasting, but more detailed analyses on the activation mechanism need to be conducted in future studies.

Referee #3:

This manuscript presents a mechanistically intriguing study describing how the transcription factor Spalt-related (Salr) functions as an integrated stress response (ISR)-activated inhibitor of mTORC1-driven growth in Drosophila fat body. The authors also describe a potentially novel regulatory mechanism by which Salr antagonizes Foxo transcription factor activity to control expression of shared catabolic target genes during fasting. Through transcriptomics, genetics, and analysis of publicly available ChIP-seq data, the authors' show that Salr also directly induces Sestrin, PRAS40, and REPTOR to repress mTORC1, thus restraining cellular growth and metabolism. The ISR-Xrp1-Salr axis, coupled with Foxo activity, provides clues linking nutrient status and nutrient stress duration to transcriptional control of cell growth and metabolism.

The manuscript is conceptually (and broadly) interesting, is clearly written, well organized, and the data do support some of the over-arching conclusions presented in the text. However, several of the key mechanistic claims, especially related to temporal molecular integration of Salr-mTORC1 and Salr-Foxo interactions with nutrient status (fasting), are based on indirect or heterologous evidence (described below), dampening overall enthusiasm.

We are grateful for the overall positive reception of our study and the constructive critique. We have now addressed the questions of temporal nutrient regulation of Salr-mTORC1 and Salr-FoxO axes and believe the manuscript has been significantly improved. Please find our point-by-point rebuttal to these and other concerns below.

<u>Major concerns:</u>

35. The authors show that Salr can influence Foxo cellular localization. However, the molecular basis of this Salr-Foxo transcription factor antagonism remains unclear (is it through Salr-regulation of insulin/Akt and/or other pathways, through changes in promoter co-occupancy, through changes in physical interactions with other protein complexes that control gene expression, etc?). Foxo generally acts as an activator of gene expression during fasting, and the authors propose that Salr is subsequently activated through the integrated stress response and antagonizes this Foxo function, while also inducing (sustaining) gene expression of shared targets during prolonged fasting. Although this model conceptually links Salr, the integrated stress response, and Foxo, the manuscript lacks direct genetic or molecular evidence to demonstrate how these factors (especially the antagonism of two transcription factors) functionally coordinate to temporally regulate gene expression during fasting. This is a critical gap that is essential to support the central mechanistic framework of the study.

The Reviewer asks about the mechanistic basis of this Salr-FoxO antagonism. The most well-established mechanism to inhibit FoxO nuclear localization is through AKT mediated phosphorylation (Brunet et al., 1999). Therefore, we tested the possibility that Salr expression influences AKT activity. Despite the technical challenges to analyze subtle changes in AKT phosphorylation in the fat body tissue, we now provide evidence that highly elevated Salr activity (Salr overexpression) does indeed increase AKT S505 phosphorylation. As S505 phosphorylation is necessary for FoxO inhibition (Hietakangas and Cohen, 2007), our finding aligns with the observed inhibition of nuclear FoxO localization. While this data is consistent with the conclusion that regulation of AKT activity is among the mechanisms by which Salr controls nuclear localization of FoxO, we recognize that the current evidence is not particularly compelling and it certainly does not rule out other alternatives (e.g. changes in the chromatin landscape), which are beyond the scope of the current study. Despite these limitations, we believe that this data is a valid addition to the body of evidence and have included it as ***Figure 5D****.*

The Reviewer also asks for further evidence on the temporal coordination of the Salr-FoxO antagonism. To address this, we have conducted a time course experiment to analyze subcellular localization of FoxO during short (2 h), intermediate (5 h) and long (16 h) fasting. Our data shows that while FoxO nuclear localization attenuates during 16 h fasting in control animals, it remains sustained in Salr deficient fat bodies. This provides the requested causal evidence, linking the attenuation of nuclear FoxO to Salr activation during prolonged fasting. We have added the new data as ***Figure 5C*** *and* ***Figure EV5A*** *and amended the text accordingly.*

36. The discussion extends the Salr-Foxo paradigm to human SALL1/FOXO1 and tumor suppression in pathological states based primarily on ChIP-seq co-occupancy. However, without any functional data in mammalian cells, these conclusions should be tempered.

*The Reviewer raises the concern about too far-reaching conclusions regarding the possible tumor suppression function of the SALL1/FOXO1 axis. We agree that this was rather far-reaching speculation. To strengthen the argument, we have now conducted additional experiments and show that HepG2 hepatocellular carcinoma cells moderately overexpressing SALL1 display impaired proliferation (****Figure 1D****), providing an additional piece of evidence on the growth-inhibitory role of Salr. To avoid overinterpretation, we have rephrased the Discussion to be more forward-looking: "Considering that mTORC1 is a major driver in HCC, being deregulated in about 50% of cases (Yu et al. 2019), it will be interesting to explore whether SALL1 and FOXO1 functionally converge to restrict mTORC1 activity in HCC, thereby suppressing tumorigenesis."*

37. Similarly to above, the authors link Salr function to mTORC1 activity, but the relationship

between these molecular events and the physiological state (nutrient status) of the animal remains unclear. In particular, it is not well explained how Salr activation during fasting is functionally connected to the suppression of mTORC1 activity. Clarifying this temporal and physiological relationship would significantly strengthen the biological relevance of the proposed model.

The Reviewer asks about the molecular events linking Salr function to mTORC1 activity. We have earlier shown that Salr regulates the expression of Sestrin, a known inhibitor of mTORC1 activity. To test the functional importance of this regulation, we have tested genetic epistasis and now show that Sestrin overexpression suppresses mTORC1 activity in Salr deficient animals and fully suppresses the tissue overgrowth observed in Salr-deficient fat bodies. Collectively, this data brings more insight into the mechanistic basis Salr-mediated regulation of mTORC1. We have added the new data as ***Figure EV3H, I****.*

The Reviewer also brings up the question of how Salr activation during fasting is temporally connected to the regulation of mTORC1 activity. We have addressed this by performing a time course experiment with short (2 h) and long (16 h) fasting in the presence and absence of Salr. We observed that while in fed animal loss of Salr leads to elevated S6 phosphorylation it is overridden by 2 h fasting. Interestingly, however, during 16 h fasting, S6 phosphorylation is elevated even compared to fed state, and this elevation is significantly more pronounced in Salr knockout animals compared to controls. While the mTORC1 activation upon prolonged fasting appears counterintuitive, similar findings have been reported earlier in mammalian liver (Kaade et al., 2024). In sum, the role of Salr in suppressing mTORC1 activity is dynamically regulated during fasting. The new data has been included as ***Figure 3G*** *and writing has been edited accordingly.*

38. The statement "Through systematic analysis of transcription factors, we discovered a novel growth inhibitory role for Spalt-related (Salr) ..." is central to the paper. However, the screening strategy is not clearly described. More detail is needed to know how the transcription factors were selected, and how Salr was identified as a key hit.

The original in vitro screen, which eventually led to the discovery of fat body specific function Salr, was thematically unrelated to the manuscript. In order to keep the focus, we have opted not to include this data, but will report it separately. We have now eliminated the sentence about systematic analysis.

39. In the sentence "In addition to systemic growth regulation, Salr overexpression in fat body clones (Tubulin [flipout] Gal4) strongly reduced cell size demonstrating cell autonomous function (Figure 1C).", the evidence for reduced cell size is not entirely clear from the presented

image. While the Salr-overexpressing cells are visible, the size of the surrounding fat body cells is less well defined, making it difficult to fully appreciate the magnitude of the size difference. It would strengthen the conclusion if the authors could provide clearer comparative visualization or quantitative analysis to more convincingly demonstrate this effect.

The Reviewer asks for a comparative analysis of cell size in Salr overexpressing clones. We have now performed measurements of cell size, confirming the original conclusion (new data in ***Figure EV1A****).*

40. The authors show that fat body-specific Salr overexpression reduces whole larval TAG levels (Figure 1I). Given this clear phenotype, it would be important and informative to also examine whether Salr knockdown or knockout leads to increased TAG levels.

As requested, we conducted a TAG measurement from the whole animal after fat body specific Salr knockout. The TAG levels do not show statistically significant change (Figure below). This may be due to biological reasons (differential TAG distribution between fat body and midgut) or technical reasons (high variation). We have now opted to eliminate the Salr overexpression data regarding systemic TAG levels and thus only focus on fat body lipid droplet analysis. The main focus of our manuscript is on growth regulation and cross-talk between growth regulatory pathways. Thus, this elimination does not change the main conclusions of our study.

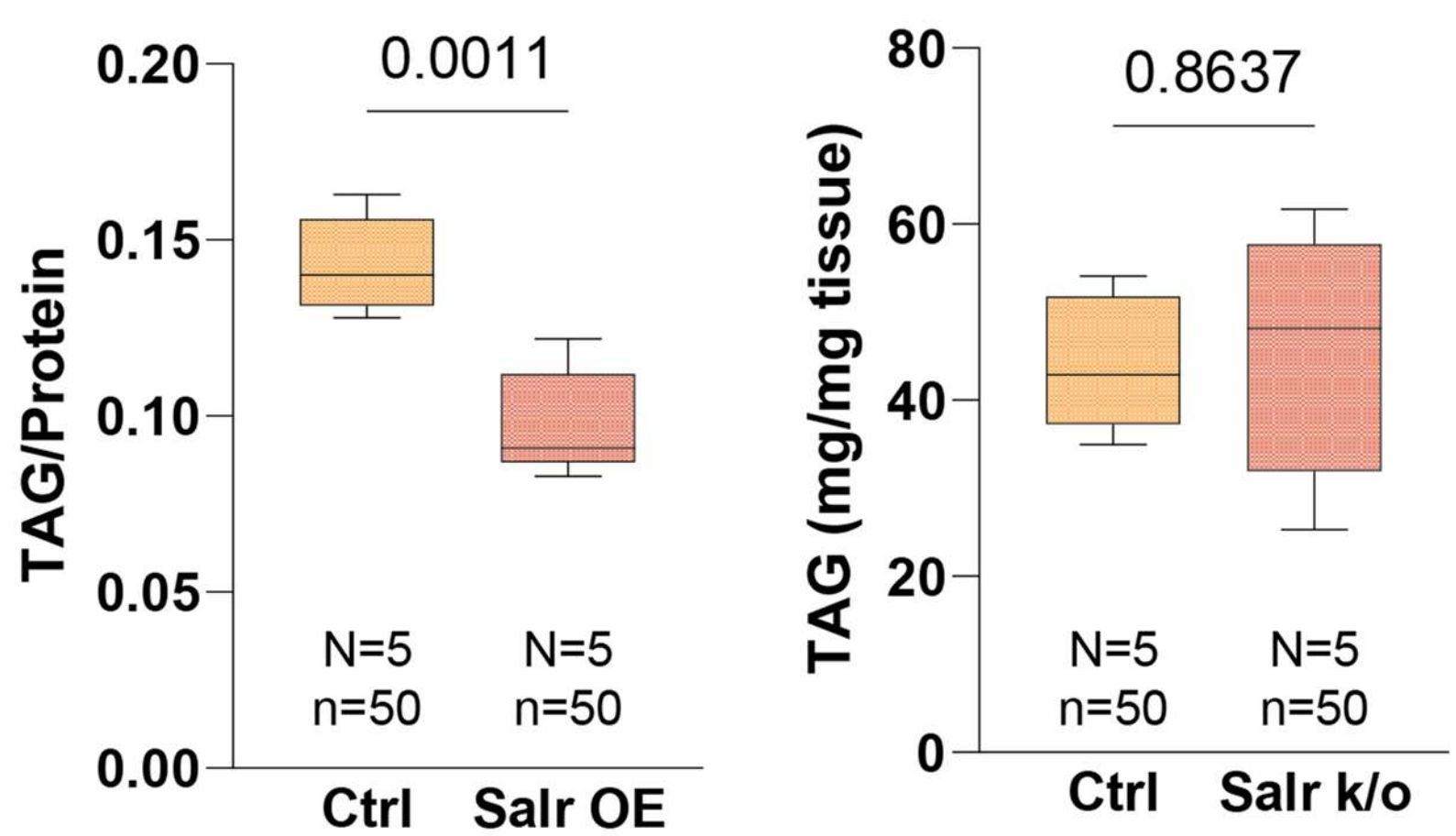

41. In Figure 2A the authors use a Salr-GFP reporter and show increased Salr expression after 5 h fasting, whereas in Figure 5 they extend fasting to 16 h to argue that Salr activation is delayed relative to Foxo. Given that Salr activation is already detectable at 5 h, could the authors clarify whether Foxo is antagonized by Salr at this earlier time point? It would strengthen the temporal model to include measurements of Salr levels and Foxo nuclear/cytoplasmic ratios at 5 h whether early Salr induction measurably reduces Foxo nuclear localization.

The Reviewer asks us to clarify whether Salr antagonizes FoxO at 5 h of fasting. We have now analyzed this by measuring FoxO nuclear/cytoplasmic ratio at different time points (fed, 2, 5, 16 h fast), in the presence and absence of Salr. Our result shows that, even though Salr expression starts to be increased after 5 h fasting (Figure 2A & B), it does not antagonize FoxO activity. This is in contrast to prolonged fasting (16 h) at which point FoxO nuclear localization is attenuated in control, but remains nuclear in the absence of Salr. The relevant data is now included as ***Figure 5C*** *(2 h and 16 h fasting) and* ***Figure EV5A*** *(5 h fasting).*

42. In Figures 2 and S2, the authors propose that Salr activation during fasting plays a key role in controlling catabolic gene expression during nutrient deprivation. However, there is no direct evidence demonstrating whether Salr knockdown during nutrient deprivation affects the fasting-dependent induction of these catabolic genes. Including such data would be critical to support the conclusion that Salr is required for catabolic gene activation in response to changes in nutrient status.

The Reviewer points out that the manuscript lacks direct evidence about catabolic gene regulation by Salr during fasting. We agree with this conclusion. We tried addressing this point, but it appeared to be challenging due to the small size of the fat body tissue during prolonged fasting, and we failed to achieve robust gene expression data. As the main point of the manuscript is the role of Salr as an integrated stress response regulated modulator of mTORC1 and FoxO activities during prolonged fasting (these aspects much strengthened during revision), we have opted to adjust our conclusions regarding catabolic gene expression (e.g. lines 99 and 109-115) to match with the current set of evidence to avoid too far-reaching conclusions.

43. The authors conclude that Salr inhibits growth by restricting mTORC1 activity, largely based on changes in nuclear and nucleolar size. It would be helpful to compliment these measurements with other growth-related parameters (e.g., larval size, weight, or developmental timing) to substantiate the claim that Salr regulates larval growth through inhibiting mTORC1 activity.

The Reviewer asks for further evidence on Salr function on organismal growth. The original manuscript included data on developmental timing and larval growth upon fat body specific overexpression of Salr. We have now complemented this analysis by Salr loss-of-function data. We observed that fat body-specific knockout of Salr does not influence developmental timing, but it increases the pupal volume, consistent with the growth inhibitory role of Salr. This phenocopies the effect of fat body FoxO RNAi (Delanoue et al., 2010). We have included this data as ***Figure 1E*** *and* ***Figure EV1F****.*

Minor comments:

44. The authors used the CRISPR/Cas12a system to knock out Salr specifically in the fat body, which is an appropriate and effective strategy for generating targeted gene deletions. However, no direct evidence is provided to confirm that Salr was successfully knocked out in the targeted tissue.

We have confirmed the fat body-specific Salr knockout by monitoring the levels of endogenously tagged Salr-GFP fusion protein. These results are now in ***Figure EV1E****.*

45. The manuscript refers to Figure S1C, but this panel does not appear to be included in the Supplementary Figure 1.

Corrected.

46. In Figure 5A, the nuclei in AKT-i cells appear noticeably smaller than those in the control group. Please check whether the magnification or scaling between the two groups is consistent.

We checked this and it is correct.

47. It appears the Y-axis is mislabeled in Figure 1C (third panel).

Corrected.

References

Barrio, R., Shea, M.J., Carulli, J., Lipkow, K., Gaul, U., Frommer, G., Schuh, R., Jäckle, H., Kafatos, F.C., 1996. The spalt-related gene of Drosophila melanogaster is a member of an ancient gene family, defined by the adjacent, region-specific homeotic gene spalt. Dev. Genes Evol. 206, 315–325. https://doi.org/10.1007/s004270050058

Brunet, A., Bonni, A., Zigmond, M.J., Lin, M.Z., Juo, P., Hu, L.S., Anderson, M.J., Arden, K.C., Blenis, J., Greenberg, M.E., 1999. Akt Promotes Cell Survival by Phosphorylating and Inhibiting a Forkhead Transcription Factor. Cell 96, 857–868. https://doi.org/10.1016/S0092-8674(00)80595-4

Delanoue, R., Slaidina, M., Léopold, P., 2010. The steroid hormone ecdysone controls systemic growth by repressing dMyc function in Drosophila fat cells. Dev. Cell 18, 1012–1021. https://doi.org/10.1016/j.devcel.2010.05.007

Hietakangas, V., Cohen, S.M., 2007. Re-evaluating AKT regulation: role of TOR complex 2 in tissue growth. Genes Dev. 21, 632–637. https://doi.org/10.1101/gad.416307

Kaade, E., Mausbach, S., Erps, N., Sylvester, M., Shakeri, F., Jachimowicz, R.D., Gieselmann, V., Thelen, M., 2024. Starvation-induced metabolic rewiring affects mTORC1 composition in vivo. Sci. Rep. 14, 28296. https://doi.org/10.1038/s41598-024-78873-7

Liu, Y., Mattila, J., Ventelä, S., Yadav, L., Zhang, W., Lamichane, N., Sundström, J., Kauko, O., Grénman, R., Varjosalo, M., Westermarck, J., Hietakangas, V., 2017. PWP1 Mediates Nutrient-Dependent Growth Control through Nucleolar Regulation of Ribosomal Gene Expression. Dev. Cell 43, 240-252.e5. https://doi.org/10.1016/j.devcel.2017.09.022

Ochs, R.L., Lischwe, M.A., Spohn, W.H., Busch, H., 1985. Fibrillarin: a new protein of the nucleolus identified by autoimmune sera. Biol. Cell 54, 123–133. https://doi.org/10.1111/j.1768-322x.1985.tb00387.x

Orengo, D.J., Aguadé, M., Juan, E., 2017. Characterization of dFOXO binding sites upstream of the Insulin Receptor P2 promoter across the Drosophila phylogeny. PloS One 12, e0188357. https://doi.org/10.1371/journal.pone.0188357

Roux, P.P., Shahbazian, D., Vu, H., Holz, M.K., Cohen, M.S., Taunton, J., Sonenberg, N., Blenis, J., 2007. RAS/ERK signaling promotes site-specific ribosomal protein S6 phosphorylation via RSK and stimulates cap-dependent translation. J. Biol. Chem. 282, 14056–14064. https://doi.org/10.1074/jbc.M700906200

Ru, W., Koga, T., Wang, X., Guo, Q., Gearhart, M.D., Zhao, S., Murphy, M., Kawakami, H., Corcoran, D., Zhang, J., Zhu, Z., Yao, X., Kawakami, Y., Xu, C., 2022. Structural studies of SALL family protein zinc finger cluster domains in complex with DNA reveal preferential binding to an AATA tetranucleotide motif. J. Biol. Chem. 298, 102607. https://doi.org/10.1016/j.jbc.2022.102607

1st Revision - Editorial Decision 18th Jun 2026

Dear Ville,

Thank you for submitting a revised version of your manuscript to The EMBO journal. Your study has now been seen by two of the original referees, who find that their previous concerns have been sufficiently addressed and now recommend publication of the manuscript after minor revisions.

In addition to the remaining reviewer requests, there are a few editorial points that need to be addressed before I can extend the official acceptance of the manuscript:

1. Please note that it is The EMBO Journal policy for the transcript of the editorial process (containing referee reports and your response letters) to be published as an online supplement to each paper. If you should prefer removal of any referee-only figures included in the point-by-point response(s), e.g. because they may still be used for future publication or because they have been reproduced from published work by others, please do let us know immediately via response email.
More information is available here: https://www.embopress.org/transparent-process#Review_Process
2. The headings and order of the sections in the manuscript text should be as follows: Abstract / Introduction / Results / Discussion / Methods / Data Availability / Acknowledgements / Disclosure and Competing Interests Statement / References / Figure Legends / Expanded View Figure Legends.
3. CRediT has replaced the traditional author contributions section because it offers a systematic, machine-readable author contributions format that allows for more effective research assessment. Please remove the Authors Contributions from the manuscript and use the free text boxes beneath each contributing author's name in our online submission system to add specific details on the author's contribution. More information is available in our guide to authors.
4. Please update references according to The EMBO Journal style - where there are more than 10 authors on a paper, the first 10 should be listed, followed by 'et al.' DOI should be only used for datasets or preprints that are not yet published.
5. There is a reference to "data not shown" in the line 201. According to our policy, which does not permit references to "data not shown", please include this information in the Appendix.
6. Please rename Appendix Table S1 into "Dataset EV1" and update the nomenclature accordingly throughout the manuscript and the files. Please add a legend to the file in a separate tab/worksheet, containing the heading and a short description of the file's contents.
7. Dataset EV1 (current Appendix Table S1) is not mentioned in the manuscript text, please check and correct.
8. During our standard image check, we noticed possible reuse between two figure panels Figure 3F and Figure EV3G. Please check - if this was intentional, please clearly state this in the corresponding figure legends.
9. Our data editors have flagged the following issues in figure legends that need correcting:
- Please provide the exact p values for values labelled as "ns".
- Please indicate the statistical test used for data analysis in the legends of figures EV-2B,C; EV-3C; EV-C.
- Please note that the box plots need to be defined in terms of minima, maxima, centre, bounds of box and whiskers, and percentile in the legends of figure 1C,E,F,G,H,I,J; 2AE,F; 3A,C,E,F,G; 4E,F; 5A-D; 6A-D; EV-1A,E,G; EV-2D-J; EV-3C,E,F,G,H,I; EV-4C; EV-5A,B; EV-6A,C,D.
- Please define the error bars in the legends of figures 1B,D; 2B,C; EV-1C,F; EV-2A; EV-4F.
10. Papers published in The EMBO Journal are accompanied online by a 'Synopsis' to enhance discoverability of the manuscript. It consists of A) a short (1-2 sentences) summary of the findings and their significance, B) 3-4 bullet points highlighting key results (the highlights currently included in the manuscript can be used here) and C) a synopsis image that is 550x300 pixels large (width x height, jpeg or png format). You can either show a model or key data in the synopsis image. Please note that the image size is rather small and that text needs to be readable at the final size.

Please let me know if you have any questions regarding any of these points. You can use the link below to upload the revised files.

Thank you again for giving us the chance to consider your manuscript for The EMBO Journal. I look forward to receiving the final version.

With best wishes,

Ieva

Ieva Gailite, PhD
Senior Scientific Editor
The EMBO Journal
Meyerhofstrasse 1
D-69117 Heidelberg
Tel: +4962218891309
i.gailite@embojournal.org

We realize that it is difficult to revise to a specific deadline. In the interest of protecting the conceptual advance provided by the work, we recommend a revision within 3 months (16th Sep 2026). Please discuss the revision progress ahead of this time with the editor if you require more time to complete the revisions.

Referee #1:

The authors' newly added data address all of my previous points. Below I list one minor point that could further enhance clarity.

1. The authors added additional RNAi data to address concerns about off-target effects. The reagent table describes multiple RNAi lines that target FoxO and XRP1, but the Figure legends do not specify which specific RNAi lines were used for the data presented. This information (the specific RNAi line used for targeting XRP1 or FoxO) should be added to either the figure legends or to the main text.

Referee #3:

The revised manuscript has been substantially improved. The authors have addressed several previous concerns by adding new experiments, including additional validation of the Salr loss-of-function phenotype, mTORC1-related assays, FoxO localization time-course analysis, and improved clarification of several figure-related issues. Overall, the revised manuscript and new data better support the proposed role of Salr as a nutrient-stress-responsive regulator of growth and mTORC1 activity in the Drosophila fat body. Congratulations to all the authors on an interesting study!

One minor concern that can be addressed editorially. The authors should further temper the conclusion that Salr controls catabolic gene expression during fasting. The current evidence supports the idea that Salr gain- and loss-of-function in fat bodies can alter a starvation-like transcriptional program, including genes involved in autophagy and lipid catabolism. However, the revised manuscript still does not directly demonstrate that Salr is required for the fasting-induced activation of these catabolic/ metabolic genes under nutrient deprivation.

Referee #1:

The authors' newly added data address all of my previous points. Below I list one minor point that could further enhance clarity.

1. The authors added additional RNAi data to address concerns about off-target effects. The reagent table describes multiple RNAi lines that target FoxO and XRP1, but the Figure legends do not specify which specific RNAi lines were used for the data presented. This information (the specific RNAi line used for targeting XRP1 or FoxO) should be added to either the figure legends or to the main text.

We have added the missing information to the figure legends.

Referee #3:

The revised manuscript has been substantially improved. The authors have addressed several previous concerns by adding new experiments, including additional validation of the Salr loss-of-function phenotype, mTORC1-related assays, FoxO localization time-course analysis, and improved clarification of several figure-related issues. Overall, the revised manuscript and new data better support the proposed role of Salr as a nutrient-stress-responsive regulator of growth and mTORC1 activity in the Drosophila fat body. Congratulations to all the authors on an

interesting study!

One minor concern that can be addressed editorially. The authors should further temper the conclusion that Salr controls catabolic gene expression during fasting. The current evidence supports the idea that Salr gain- and loss-of-function in fat bodies can alter a starvation-like transcriptional program, including genes involved in autophagy and lipid catabolism. However, the revised manuscript still does not directly demonstrate that Salr is required for the fasting-induced activation of these catabolic/metabolic genes under nutrient deprivation.

We have edited the Results section to explicitly state that "Salr is sufficient to activate a starvation-like transcriptional response in the fat bodies of fed larvae".

Dear Ville,

Thank you for submitting a revised version of your manuscript and for addressing the remaining editorial and reviewer requests. I sincerely apologise for the delay in the processing of your manuscript caused by conference travel. I am now pleased to inform you that your manuscript has been accepted for publication. Congratulations with a nice study!

Before we forward your manuscript to our publishers, we would like to propose some edits in the manuscript title, abstract and synopsis. We have also selected a short blurb that will accompany the title of your manuscript in our online table of contents. Please take a look at the proposed textual changes in the attached file - any corrections or edits from your side are very welcome.

Once I have received your input on these points, I will be happy to forward your manuscript to our publishers for typesetting.

Your manuscript will be processed for publication by EMBO Press. It will be copy edited and you will receive page proofs prior to publication. Please note that you will be contacted by Springer Nature Author Services to complete licensing and payment information.

You may qualify for financial assistance for your publication charges - either via a Springer Nature fully open access agreement or an EMBO initiative. Check your eligibility: https://link.springer.com/journal/44318/how-to-publish-with-us

Should you be planning a Press Release on your article, please notify the production team via this form in order to coordinate publication and release dates: https://forms.cloud.microsoft/e/eH4A8JVP6g

If you have any questions, please do not hesitate to contact the Editorial Office. Thank you for this interesting contribution to The EMBO Journal! I look forward to hearing from you soon regarding the final textual changes.

Best wishes,

Ieva

Ieva Gailite, PhD
Senior Scientific Editor
The EMBO Journal
Meyerhofstrasse 1
D-69117 Heidelberg
Tel: +4962218891309
i.gailite@embojournal.org

EMBO Press Author Checklist

Corresponding Author Name: Ville Hietakangas	
Journal Submitted to: EMBO Journal	
Manuscript Number: EMBOJ-2025-122623	

USEFUL LINKS FOR COMPLETING THIS FORM
The EMBO Journal - Author Guidelines
EMBO Reports - Author Guidelines
Molecular Systems Biology - Author Guidelines
EMBO Molecular Medicine - Author Guidelines

Reporting Checklist for Life Science Articles (updated January
This checklist is adapted from Materials Design Analysis Reporting (MDAR) Checklist for Authors. MDAR establishes a minimum set of requirements in transparent reporting in the life sciences (see Statement of Task: 10.31222/osf.io/9sm4x). Please follow the journal's guidelines in preparing your
Please note that a copy of this checklist will be published alongside your article.

Abridged guidelines for figures
1. Data
The data shown in figures should satisfy the following conditions:
- the data were obtained and processed according to the field's best practice and are presented to reflect the results of the experiments in an accurate and unbiased manner.
- ideally, figure panels should include only measurements that are directly comparable to each other and obtained with the same assay.
- plots include clearly labeled error bars for independent experiments and sample sizes. Unless justified, error bars should not be shown for technical
- if n<5, the individual data points from each experiment should be plotted. Any statistical test employed should be justified.
- Source Data should be included to report the data underlying figures according to the guidelines set out in the authorship guidelines on Data

2. Captions
Each figure caption should contain the following information, for each panel where they are relevant:
- a specification of the experimental system investigated (eg cell line, species name).
- the assay(s) and method(s) used to carry out the reported observations and measurements.
- an explicit mention of the biological and chemical entity(ies) that are being measured.
- an explicit mention of the biological and chemical entity(ies) that are altered/varied/perturbed in a controlled manner.
- the exact sample size (n) for each experimental group/condition, given as a number, not a range;
- a description of the sample collection allowing the reader to understand whether the samples represent technical or biological replicates (including how many animals, litters, cultures, etc.).
- a statement of how many times the experiment shown was independently replicated in the laboratory.
- definitions of statistical methods and measures:
  - common tests, such as t-test (please specify whether paired vs. unpaired), simple χ2 tests, Wilcoxon and Mann-Whitney tests, can be unambiguously identified by name only, but more complex techniques should be described in the methods section;
  - are tests one-sided or two-sided?
  - are there adjustments for multiple comparisons?
  - exact statistical test results, e.g., P values = x but not P values < x;
  - definition of 'center values' as median or average;
  - definition of error bars as s.d. or s.e.m.

Please complete ALL of the questions below.
Select "Not Applicable" only when the requested information is not relevant for your study.

Materials

Newly Created Materials	Information included in the manuscript?	In which section is the information available? (Reagents and Tools Table, Materials and Methods, Figures, Data Availability Section)
New materials and reagents need to be available; do any restrictions apply?	Yes	Reagents and Tools Table, Material and Methods

Antibodies	Information included in the manuscript?	In which section is the information available? (Reagents and Tools Table, Materials and Methods, Figures, Data Availability Section)
For **antibodies** provide the following information:<br>- Commercial antibodies: RRID (if possible) or supplier name, catalogue number and or/clone number<br>- Non-commercial: RRID or citation	Yes	Reagents and Tools Table, Material and Methods

DNA and RNA sequences	Information included in the manuscript?	In which section is the information available? (Reagents and Tools Table, Materials and Methods, Figures, Data Availability Section)
**Short novel DNA or RNA including primers, probes**: provide the sequences.	Yes	Reagents and Tools Table, Material and Methods

Cell materials	Information included in the manuscript?	In which section is the information available? (Reagents and Tools Table, Materials and Methods, Figures, Data Availability Section)
**Cell lines:** Provide species information, strain. Provide accession number in repository **OR** supplier name, catalog number, clone number, and/**OR** RRID.	Yes	Reagents and Tools Table, Material and Methods
**Primary cultures:** Provide species, strain, sex of origin, genetic modification status.	Yes	Reagents and Tools Table, Material and Methods
Report if the cell lines were recently **authenticated** (e.g., by STR profiling) and tested for mycoplasma contamination.	Yes	Cells were ordered from DSMZ, mentioned in the Reagents and Tools Table, Material and Methods

Experimental animals	Information included in the manuscript?	In which section is the information available? (Reagents and Tools Table, Materials and Methods, Figures, Data Availability Section)
**Laboratory animals or Model organisms:** Provide species, strain, sex, age, genetic modification status. Provide accession number in repository **OR** supplier name, catalog number, clone number, **OR** RRID.	Yes	Reagents and Tools Table, Material and Methods
**Animal observed in or captured from the field:** Provide species, sex, and age where possible.	Not Applicable	
Please detail **housing and husbandry conditions**.	Yes	Material and Methods

Plants and microbes	Information included in the manuscript?	In which section is the information available? (Reagents and Tools Table, Materials and Methods, Figures, Data Availability Section)
**Plants:** provide species and strain, ecotype and cultivar where relevant, unique accession number if available, and source (including location for collected wild specimens).	Not Applicable	
**Microbes:** provide species and strain, unique accession number if available, and source.	Not Applicable	

Human research participants	Information included in the manuscript?	In which section is the information available? (Reagents and Tools Table, Materials and Methods, Figures, Data Availability Section)
If collected and within the bounds of privacy constraints report on age, sex and gender or ethnicity for all study participants.	Not Applicable	

Core facilities	Information included in the manuscript?	In which section is the information available? (Reagents and Tools Table, Materials and Methods, Figures, Data Availability Section)
If your work benefited from core facilities, was their service mentioned in the acknowledgments section?	Yes	Acknowledgements

Design

Study protocol	Information included in the manuscript?	In which section is the information available? (Reagents and Tools Table, Materials and Methods, Figures, Data Availability Section)
If study protocol has been **pre-registered, provide DOI in the manuscript**. For clinical trials, provide the trial registration number **OR** cite DOI.	Not Applicable	
Report the **clinical trial registration number** (at ClinicalTrials.gov or equivalent), where applicable.	Not Applicable	

Laboratory protocol	Information included in the manuscript?	In which section is the information available? (Reagents and Tools Table, Materials and Methods, Figures, Data Availability Section)
Provide DOI OR other citation details if **external detailed step-by-step protocols** are available.	Not Applicable	

Experimental study design and statistics	Information included in the manuscript?	In which section is the information available? (Reagents and Tools Table, Materials and Methods, Figures, Data Availability Section)
Include a statement about **sample size** estimate even if no statistical methods were used.	Yes	On individual figure panels
Were any steps taken to minimize the effects of subjective bias when allocating animals/samples to treatment (e.g. **randomization procedure**)? If yes, have they been described?	Not Applicable	
Include a statement about **blinding** even if no blinding was done.	Yes	Materials and methods
Describe **inclusion/exclusion criteria** if samples or animals were excluded from the analysis. Were the criteria pre-established?<br>If sample or data points were omitted from analysis, report if this was due to attrition or intentional exclusion and provide justification.	Not Applicable	
For every figure, are **statistical tests** justified as appropriate? Do the data meet the assumptions of the tests (e.g., normal distribution)? Describe any methods used to assess it. Is there an estimate of variation within each group of data? Is the variance similar between the groups that are being statistically compared?	Yes	Figure legends, Materials and Methods

Sample definition and in-laboratory replication	Information included in the manuscript?	In which section is the information available? (Reagents and Tools Table, Materials and Methods, Figures, Data Availability Section)
In the figure legends: state number of times the experiment was **replicated** in laboratory.	Yes	Figure legends
In the figure legends: define whether data describe **technical or biological replicates**.	Yes	Figure legends

Ethics

Ethics	Information included in the manuscript?	In which section is the information available? (Reagents and Tools Table, Materials and Methods, Figures, Data Availability Section)
Studies involving **human participants**: State details of **authority granting ethics approval** (IRB or equivalent committee(s), provide reference number for approval.	Not Applicable	
Studies involving **human participants**: Include a statement confirming that **informed consent** was obtained from all subjects and that the experiments conformed to the principles set out in the WMA Declaration of Helsinki and the Department of Health and Human Services Belmont Report.	Not Applicable	
Studies involving **human participants: For publication of patient photos**, include a statement confirming that consent to publish was obtained.	Not Applicable	
Studies involving experimental **animals**: State details of **authority granting ethics approval** (IRB or equivalent committee(s), provide reference number for approval. Include a statement of compliance with ethical regulations.	Not Applicable	
Studies involving **specimen and field samples:** State if relevant **permits** obtained, provide details of authority approving study; if none were required, explain why.	Not Applicable	

Dual Use Research of Concern (DURC)	Information included in the manuscript?	In which section is the information available? (Reagents and Tools Table, Materials and Methods, Figures, Data Availability Section)
Could your study fall under dual use research restrictions? Please check biosecurity documents and list of **select agents and toxins** (CDC): https://www.selectagents.gov/sat/list.htm	Not Applicable	
If you used a select agent, is the security level of the lab appropriate and reported in the manuscript?	Not Applicable	
If a study is subject to dual use research of concern regulations, is the name of the **authority granting approval and reference number** for the regulatory approval provided in the manuscript?	Not Applicable	

Reporting

The MDAR framework recommends adoption of discipline-specific guidelines, established and endorsed through community initiatives. Journals have their own policy about requiring specific guidelines and recommendations to complement MDAR.

Adherence to community standards	Information included in the manuscript?	In which section is the information available? (Reagents and Tools Table, Materials and Methods, Figures, Data Availability Section)
State if relevant guidelines or checklists (e.g., **ICMJE, MIBBI, ARRIVE, PRISMA**) have been followed or provided.	Not Applicable	
For **tumor marker prognostic studies,** we recommend that you follow the **REMARK** reporting guidelines (see link list at top right). See author guidelines, under 'Reporting Guidelines'. Please confirm you have followed these guidelines.	Not Applicable	
For **phase II and III randomized controlled trials**, please refer to the **CONSORT** flow diagram (see link list at top right) and submit the CONSORT checklist (see link list at top right) with your submission. See author guidelines, under 'Reporting Guidelines'. Please confirm you have submitted this list.	Not Applicable	

Data Availability

Data availability	Information included in the manuscript?	In which section is the information available? (Reagents and Tools Table, Materials and Methods, Figures, Data Availability Section)
Have **primary datasets** been deposited according to the journal's guidelines (see 'Data Deposition' section) and the respective accession numbers provided in the Data Availability Section?	Yes	Data Availability
Were **human clinical and genomic datasets** deposited in a public access-controlled repository in accordance to ethical obligations to the patients and to the applicable consent agreement?	Not Applicable	
Are **computational models** that are central and integral to a study available without restrictions in a machine-readable form? Were the relevant accession numbers or links provided?	Not Applicable	
If publicly available data were reused, provide the respective **data citations in the reference list**.	Yes	Materials and Methods, References
