# Supplementary material for: Spalt-related is an inhibitor of mTORC1-mediated growth activated by the integrated stress response
Source: EMBO J. 2026 Jul 11;45(16):5919–42. doi: 10.1038/s44318-026-00858-1 (PMC13482101; doi:10.1038/s44318-026-00858-1)
Supplement: Supplementary file 2 [file 44318_2026_858_MOESM3_ESM.pdf]

Expanded View Figures

Figure EV1. Salr inhibits cell growth in the fat body, related to Fig. 1.

(**A**) Salr regulates cell size in the fat body. Representative images of volume quantification of Salr overexpressing clones (Tubulin flipout) and control cells. Quantification of cell volume upon gain- and loss-of-function of Salr in the fat body. (**B**) Representative images of anti-fibrillarin staining of Salr CRISPR/Cas12a knockout and control fat body cells. Fibrillarin localizes to the nucleolus and colocalizes with the DAPI-deficient area. (**C**) qPCR analysis of *SALL1* mRNA levels from SALL1 stably transfected HepG2 cells. (**D**) Salr CRISPR/Cas12a target sites on wild-type Salr protein. (**E**) Confirmation of Salr CRISPR/Cas12a knockout using GFP-tagged Salr reporter. (**F**) Fat body-specific Salr CRISPR/Cas12a knockout does not lead to accelerated pupariation. (**G**) Salr knockdown with Dicer2 increases nucleus size. Representative image of fat body cells that are Salr knockdown with Dicer2, control, and Dicer2, stained with DAPI. Quantification of nucleus size. *P* values in (**A–C**, **G**) were calculated with unpaired *t* test with Welch correction. *P* values in (**E**) were obtained by one-way ANOVA followed by Tukey's test with multiple comparison correction. *P* value and chi-square in (**F**) were calculated by the log-rank test for trend. N = biological replicates, n = technical replicates. In box plots, the center line represents the median, the box boundaries represent the 25th and 75th percentiles, and the whiskers represent the minimum and maximum observed values. Error bars display standard deviations (SD).

 © The Author(s)

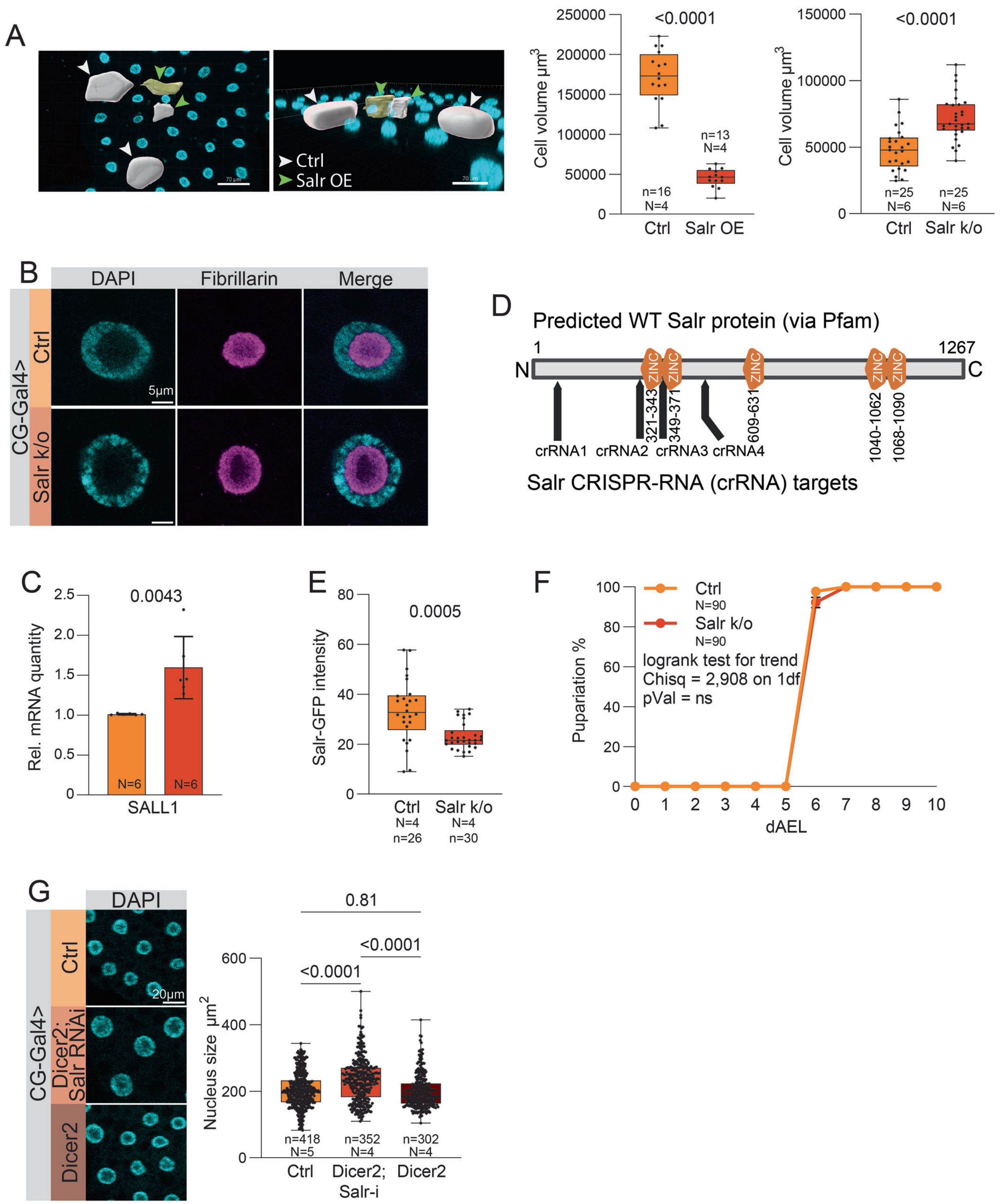
A
Ctrl
Salr OE
Cell volume µm3
<0.0001
n=16 N=4
n=13 N=4
Ctrl Salr OE
<0.0001
n=25 N=6
n=25 N=6
Ctrl Salr k/o
B
DAPI Fibrillarin Merge
CG-Gal4>
Ctrl
Salr k/o
5µm
C
Rel. mRNA quantity
0.0043
N=6 N=6
SALL1
D
Predicted WT Salr protein (via Pfam)
N 1 1267 C
ZINC
321-343 349-371 609-631 1040-1062 1068-1090
crRNA1 crRNA2 crRNA3 crRNA4
Salr CRISPR-RNA (crRNA) targets
E
Salr-GFP intensity
0.0005
Ctrl N=4 n=26
Salr k/o N=4 n=30
F
Pupariation %
Ctrl N=90
Salr k/o N=90
logrank test for trend
Chisq = 2,908 on 1df
pVal = ns
dAEL
G
DAPI
CG-Gal4>
Ctrl
Dicer2; Salr RNAi
Dicer2
20µm
Nucleus size µm2
0.81
<0.0001
<0.0001
n=418 N=5
n=352 N=4
n=302 N=4
Ctrl Dicer2; Salr-i Dicer2

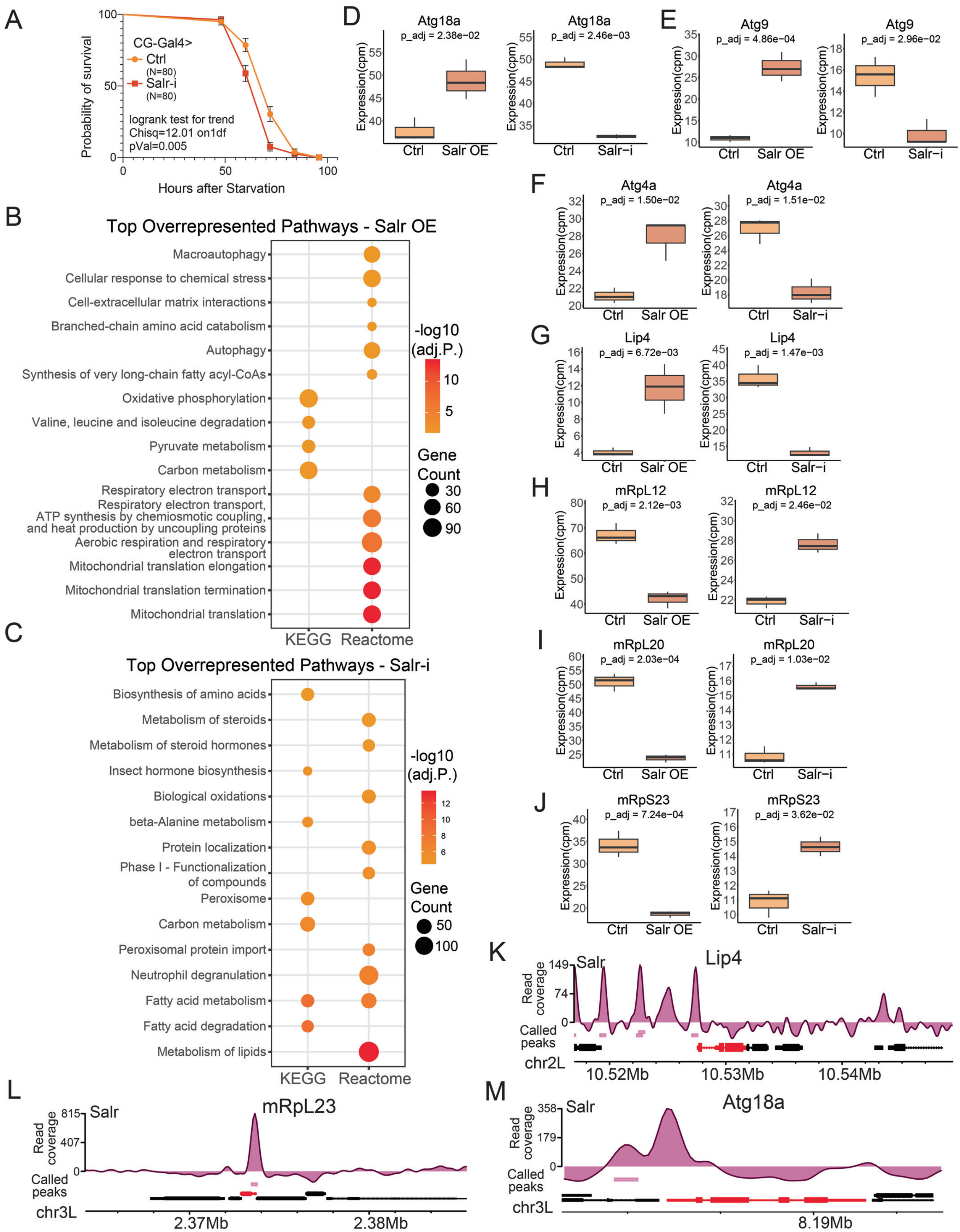
A
CG-Gal4>
Ctrl (N=80)
Salr-i (N=80)
logrank test for trend
Chisq=12.01 on1df
pVal=0.005
Probability of survival
Hours after Starvation
B
Top Overrepresented Pathways - Salr OE
Macroautophagy
Cellular response to chemical stress
Cell-extracellular matrix interactions
Branched-chain amino acid catabolism
Autophagy
Synthesis of very long-chain fatty acyl-CoAs
Oxidative phosphorylation
Valine, leucine and isoleucine degradation
Pyruvate metabolism
Carbon metabolism
Respiratory electron transport
Respiratory electron transport, ATP synthesis by chemiosmotic coupling, and heat production by uncoupling proteins
Aerobic respiration and respiratory electron transport
Mitochondrial translation elongation
Mitochondrial translation termination
Mitochondrial translation
KEGG Reactome
-log10 (adj.P.)
Gene Count
C
Top Overrepresented Pathways - Salr-i
Biosynthesis of amino acids
Metabolism of steroids
Metabolism of steroid hormones
Insect hormone biosynthesis
Biological oxidations
beta-Alanine metabolism
Protein localization
Phase I - Functionalization of compounds
Peroxisome
Carbon metabolism
Peroxisomal protein import
Neutrophil degranulation
Fatty acid metabolism
Fatty acid degradation
Metabolism of lipids
KEGG Reactome
D
Atg18a p_adj = 2.38e−02
Atg18a p_adj = 2.46e−03
E
Atg9 p_adj = 4.86e−04
Atg9 p_adj = 2.96e−02
F
Atg4a p_adj = 1.50e−02
Atg4a p_adj = 1.51e−02
G
Lip4 p_adj = 6.72e−03
Lip4 p_adj = 1.47e−03
H
mRpL12 p_adj = 2.12e−03
mRpL12 p_adj = 2.46e−02
I
mRpL20 p_adj = 2.03e−04
mRpL20 p_adj = 1.03e−02
J
mRpS23 p_adj = 7.24e−04
mRpS23 p_adj = 3.62e−02
Expression(cpm)
Ctrl Salr OE
Ctrl Salr−i
K
Salr Lip4
Read coverage
Called peaks
chr2L
10.52Mb 10.53Mb 10.54Mb
L
Salr mRpL23
chr3L
2.37Mb 2.38Mb
M
Salr Atg18a
chr3L
8.19Mb

 © The Author(s)

Figure EV2. Salr regulates catabolic gene expression, related to Fig. 2.

(**A**) Salr knockdown in the fat body impairs survival upon fasting. (**B**, **C**) Unselected top overrepresented pathways from fat body-specific Salr overexpression and knockdown RNA-Seq datasets, ranked based on their adjusted *P* values. (**D–M**) Salr overexpression and knockdown in the fat body significantly regulate genes involved in lipid metabolism, autophagy, mitochondrial ribosome biogenesis, some of which are direct targets of Salr. (**D–J**) *Atg18a*, *Atg9*, *Atg4a*, *Lip4*, *mRpL12*, *mRpL20*, *mRpL23* expression from RNA-Seq datasets upon fat body-specific Salr overexpression and knockdown. (**K–M**) Called peaks and track coverage of Salr ChIP-Seq on *Lip4, Atg18a*, and *mRpL23* genes highlighted in red. *P* value and chi-square in (**A**) were calculated by the log-rank test for trend. Adjusted *P* values in (**B**, **C**) were calculated by a one-sided hypergeometric test for over-representation analysis and adjusted using the Benjamini–Hochberg method. Adjusted *P* values in (**D–J**) were obtained in differential expression analysis of RNA-Seq with Benjamini–Hochberg correction. $N =$ biological replicates. In box plots, the center line represents the median, the box boundaries represent the 25th and 75th percentiles, and the whiskers represent the minimum and maximum observed values. Error bars display standard deviations (SD).

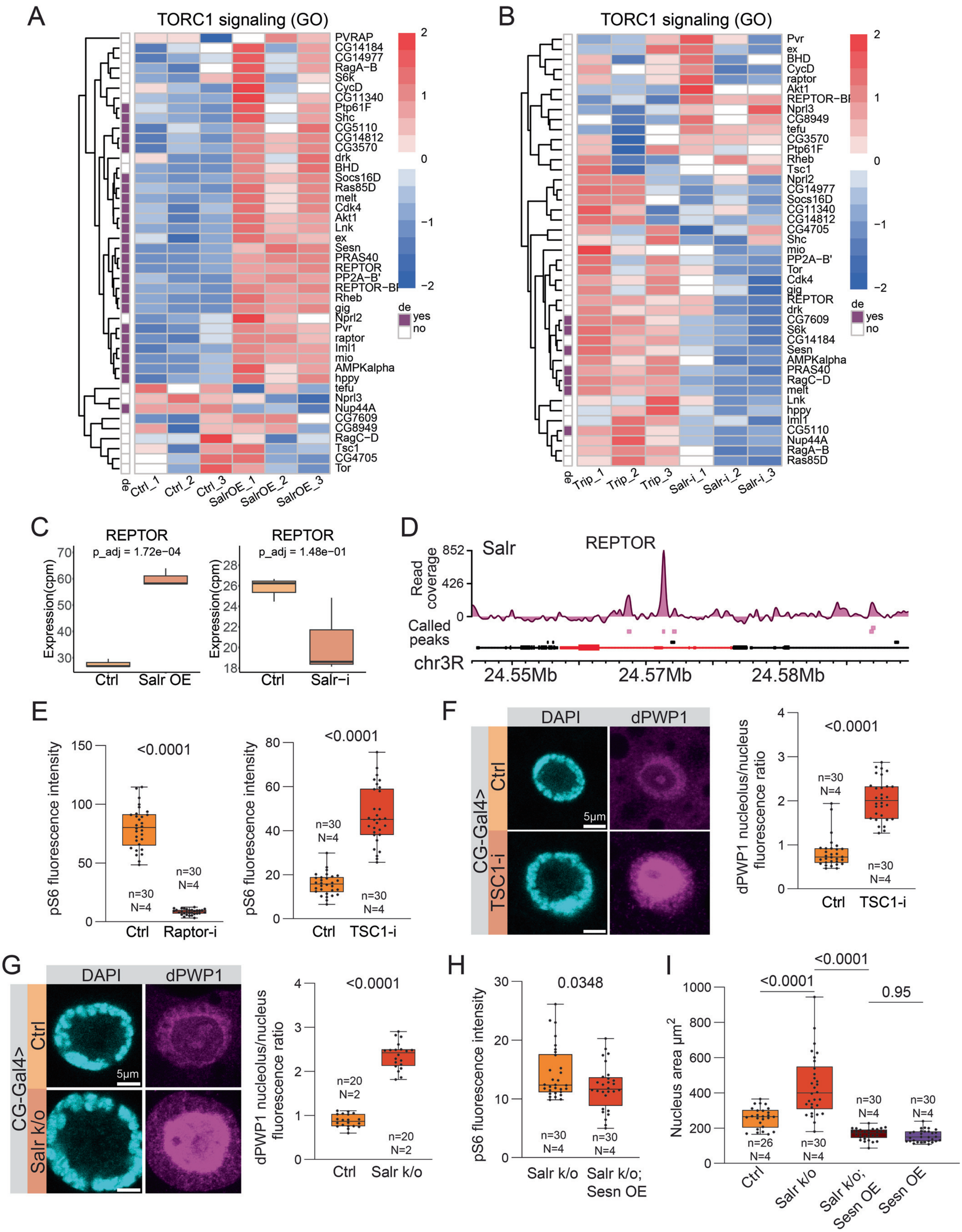
A
TORC1 signaling (GO)
de
yes
no
Ctrl_1 Ctrl_2 Ctrl_3 SalrOE_1 SalrOE_2 SalrOE_3
B
TORC1 signaling (GO)
Trip_1 Trip_2 Trip_3 Salr-i_1 Salr-i_2 Salr-i_3
C
REPTOR
p_adj = 1.72e-04
Expression(cpm)
Ctrl Salr OE
REPTOR
p_adj = 1.48e-01
Ctrl Salr-i
D
Salr
REPTOR
Read coverage
Called peaks
chr3R
24.55Mb 24.57Mb 24.58Mb
E
pS6 fluorescence intensity
<0.0001
Ctrl Raptor-i
Ctrl TSC1-i
F
DAPI dPWP1
CG-Gal4>
Ctrl
TSC1-i
5μm
dPWP1 nucleolus/nucleus fluorescence ratio
G
DAPI dPWP1
Ctrl
Salr k/o
Ctrl Salr k/o
H
0.0348
Salr k/o Salr k/o; Sesn OE
I
Nucleus area μm²
0.95
Ctrl Salr k/o Salr k/o; Sesn OE Sesn OE

Figure EV3. Salr regulates components of the mTORC1 pathway, related to Fig. 3.

(**A**, **B**) Salr regulates the mTORC1 signaling pathway. Heatmaps of the GO database-defined mTORC1 signaling pathway from Salr overexpression and knockdown RNA-Seq datasets. (**C**, **D**) Salr targets and regulates *REPTOR* expression. (**C**) *REPTOR* expression from RNA-Seq showing up- and downregulation upon fat body-specific Salr overexpression and knockdown, respectively. (**D**) Called peaks and track coverage of Salr ChIP-Seq on *REPTOR* gene highlighted in red. (**E**) phospho-S6 (p-S6) antibody responds to mTORC1 genetic manipulation. Quantification of p-S6 signal of fat body-specific Raptor and TSC1 knockdown for inhibition and promotion of mTORC1, respectively. (**F**) PWP1 localizes to the nucleolus upon constitutive mTORC1 activation by fat body-specific TSC1 knockdown. Representative images of fat body-specific TSC1 knockdown and control fat body cells stained with PWP1 antibody. Quantification of nucleolar versus nucleoplasmic PWP1 ratio. (**G**) Fat body-specific Salr CRISPR/Cas12a knockout leads to relocalization of PWP1 into the nucleolus. Representative images of fat body-specific Salr CRISPR/Cas12a knockout and control fat body cells stained with PWP1 antibody. Quantification of nucleolar versus nucleoplasmic PWP1 ratio. (**H**, **I**) Fat body-specific overexpression of Sesn rescues Salr. (**H**) Quantification of phospho-S6 of fat body-specific Salr CRISPR/Cas12a knockout and in combination with Sesn overexpression. (**I**) Quantification of the nucleus size of fat body-specific Salr CRISPR/Cas12a knockout and in combination with Sesn overexpression. Adjusted *P* values in (**C**) were obtained in differential expression analysis of RNA-Seq with Benjamini–Hochberg correction. *P* values in (**E–H**) were calculated with unpaired *t* test with Welch correction. *P* values in (**I**) were obtained by one-way ANOVA followed by Tukey's test with multiple comparison correction. $N =$ biological replicates, $n =$ technical replicates. In box plots, the center line represents the median, the box boundaries represent the 25th and 75th percentiles, and the whiskers represent the minimum and maximum observed values. Error bars display standard deviations (SD).

© The Author(s)

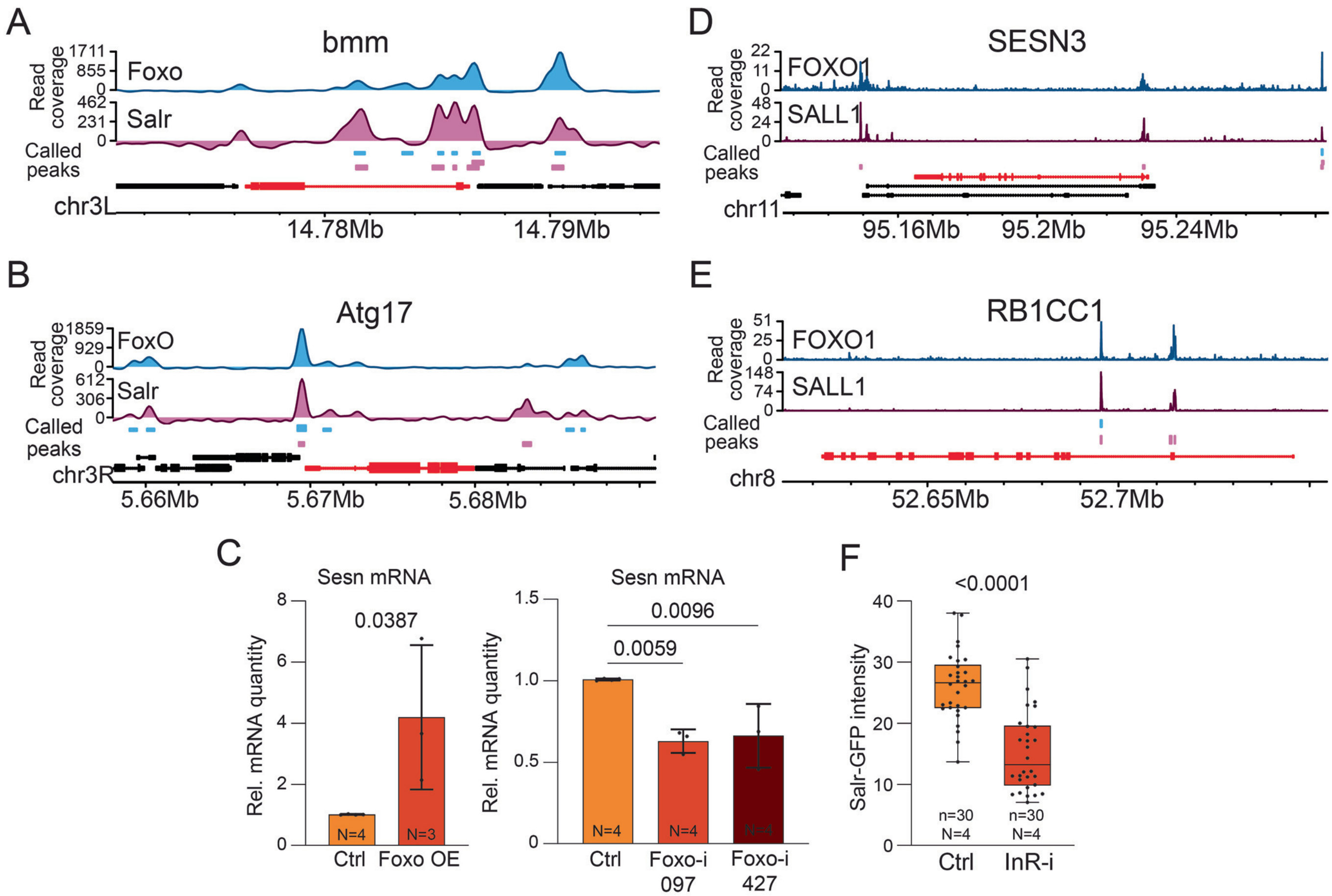

Figure EV4. Salr shares common targets with FoxO, related to Fig. 4.

(**A**) Called peaks and track coverage of Salr and FoxO ChIP-Seq on *bmm*. (**B**) Called peaks and track coverage of Salr and FoxO ChIP-Seq on *Atg17*. (**C**) FoxO positively regulates Sesn. qPCR of Sesn mRNA in FoxO gain- and loss-of-function fat bodies. Foxo-i 097: VDRC 106097, Foxo-i 427: BDSC 32427. (**D**) Called peaks and track coverage of SALL1 and FOXO1 ChIP-Seq on *SESN3* (one of the human homologs of *Drosophila* Sesn). (**E**) Called peaks and track coverage of SALL1 and FOXO1 ChIP-Seq on *RB1CC1* (human ortholog of Atg17). (**F**) InR knockdown does not activate Salr. Quantification of Salr-GFP intensity of fat body-specific InR knockdown and control. *P* values in (**C**) (left panel) and (**F**) were calculated with unpaired *t* test with Welch correction. *P* values in (**C**) (right panel) were obtained by one-way ANOVA followed by Tukey's test with multiple comparison correction. *N* = biological replicates, *n* = technical replicates. In box plots, the center line represents the median, the box boundaries represent the 25th and 75th percentiles, and the whiskers represent the minimum and maximum observed values. Error bars display standard deviations (SD).

 © The Author(s)

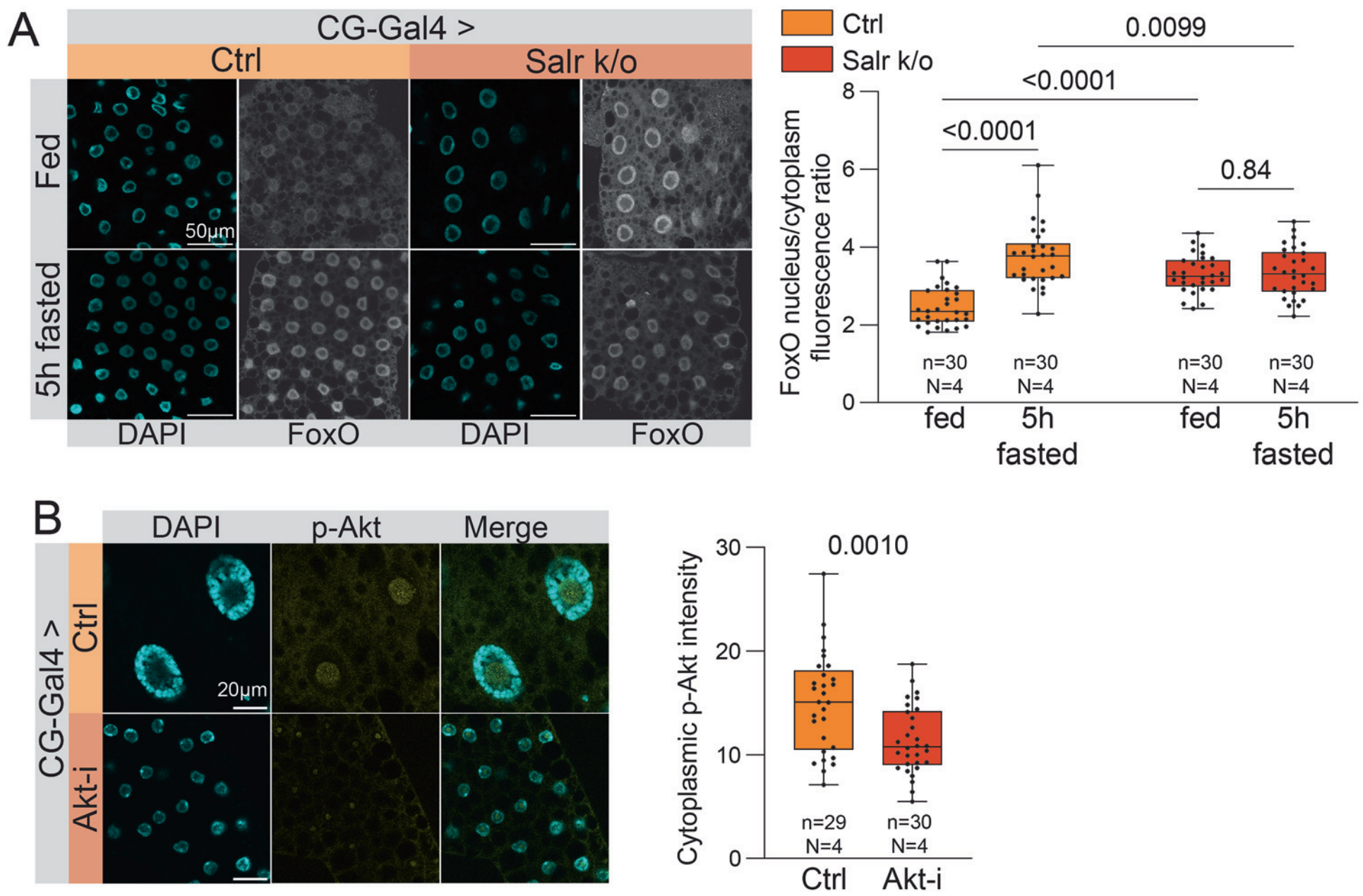

Figure EV5. Salr and FoxO show different activation dynamics upon fasting, related to Fig. 5.

(**A**) Salr CRISPR/Cas12a knockout does not activate FoxO compared to control at 5 h fasting. Representative images of Salr CRISPR/Cas12a knockout and control stained with FoxO in fat body-specific cells. Quantification of nuclear versus cytoplasmic FoxO signal. (**B**) Cytoplasmic p-Akt signal is reduced, unlike the nucleolar signal, upon Akt knockdown in the fat body. Representative images of fat body-specific Akt knockdown and control stained with p-Akt. Quantification of cytoplasmic p-Akt signal. *P* values in (**A**) were calculated with unpaired *t* test with Welch correction. *P* values in (**B**) were obtained by one-way ANOVA followed by Tukey's test with multiple comparison correction. *N* = biological replicates, *n* = technical replicates. In box plots, the center line represents the median, the box boundaries represent the 25th and 75th percentiles, and the whiskers represent the minimum and maximum observed values. Error bars display standard deviations (SD).

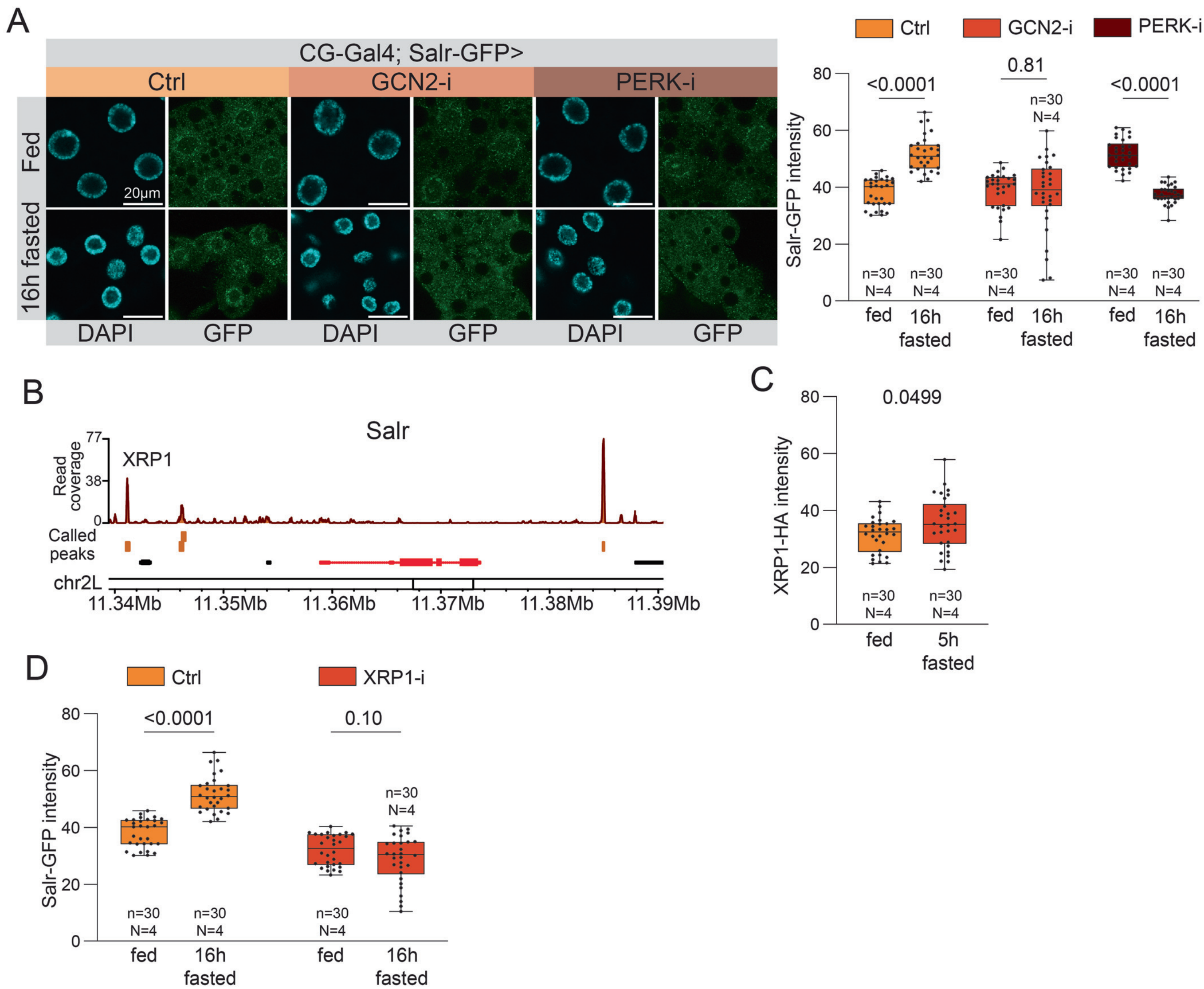

Figure EV6. Prolonged fasting activates Salr through the integrated stress response pathway, related to Fig. 6.

(**A**) Fat body-specific knockdown of GCN2 and PERK prevents Salr activation upon 16 h fasting. Representative images of GCN2 and PERK RNAi, and control fat bodies stained with GFP for Salr-GFP. Quantification of nuclear Salr-GFP. (**B**) XRP1 targets Salr. Called peaks and track coverage of XRP1 ChIP-Seq on *Salr* gene which is highlighted in red. (**C**) XRP1 level is moderately elevated upon 5 h fasting. Quantification of nuclear XRP1-HA. (**D**) XRP1 is necessary for Salr activation upon prolonged fasting. Validation of the data presented in Fig. 6D by an independent RNAi line (BDSC 51054). Quantification of Salr-GFP. P values in (**A–D**) were calculated with an unpaired *t* test with Welch correction. In box plots, the center line represents the median, the box boundaries represent the 25th and 75th percentiles, and the whiskers represent the minimum and maximum observed values. Error bars display standard deviations (SD).

 © The Author(s)
